# Inactivation of *Atp7b* Copper Transporter in Intestinal Epithelial Cells Is Associated with Altered Lipid Processing and Cell Growth Machinery Independent from Hepatic Copper Accumulation and Severity of Liver Histology

**DOI:** 10.1016/j.ajpath.2025.09.015

**Published:** 2025-10-16

**Authors:** Amanda Caceres, Noreene M. Shibata, Christian D. Davalos-Gutierrez, Gaurav V. Sarode, Hisham Hussan, Margarida Bettencourt, Adriana Fontes, Hans Zischka, Svetlana Lutsenko, Marie C. Heffern, Valentina Medici

**Affiliations:** ∗ Department of Chemistry, University of California Davis, Davis, California; † Division of Gastroenterology and Hepatology, Department of Internal Medicine, University of California Davis, Davis, California; ‡ Institute of Molecular Toxicology and Pharmacology, Helmholtz Center Munich, German Research Center for Environmental Health, Neuherberg, Germany; § Institute of Toxicology and Environmental Hygiene, Technical University Munich, Munich, Germany; ¶ Department of Physiology, Johns Hopkins Medical Institutes, Baltimore, Maryland

## Abstract

The clinical manifestations of Wilson disease (WD) are related to copper accumulation in the liver and brain, but little is known about the role of other organs expressing the ATP7B copper transporter on metabolic and ultrastructural changes characterizing WD. To examine the consequences of intestinal *Atp7b* inactivation in the absence of hepatic copper accumulation, a new mouse model (*Atp7b*^ΔIEC^) characterized by enterocyte-specific *Atp7b* inactivation was generated. *Atp7b*^ΔIEC^ mice were compared with wild-type mice with the same genetic background (iWT). The *Atp7b* global knockout (*Atp7b*^–/–^) model of WD on a C57Bl/6 background was previously generated and compared with its respective wild type (WT). Hepatic copper, lipid metabolism, liver and intestine histology, and electron microscopy were assessed over time up to 30 weeks of age. Although there was no evidence of intestine copper accumulation in *Atp7b*^ΔIEC^ mice, transcriptome analysis in *Atp7b*^ΔIEC^ mice revealed changes in genes involved in AMP-activated protein kinase signaling, fatty acid metabolism, and cell cycle both with partial overlap between the intestinal epithelial cells and the liver. Mitochondrial and other ultrastructural changes were observed in the intestinal epithelial cells of both *Atp7b*^–/–^ and *Atp7b*^ΔIEC^ mice. Intestine-specific *Atp7b* deficit affects systemic metabolic pathways and intestine morphology, and hepatic metabolic perturbations are associated with intestinal dysfunction, independently from hepatic copper accumulation, providing evidence that the WD phenotype is at least partially influenced by organ-specific ATP7B variants.

## Graphical abstract

**Figure:**
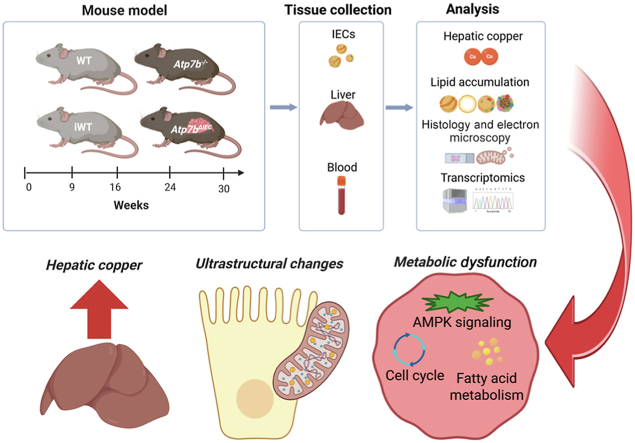

Copper metabolism and trafficking are highly regulated because of its essential role in multiple key metabolic pathways, and to avoid copper accumulation with ensuing mitochondrial dysfunction, decreased glutathione levels, promutagenic DNA adducts, and oxidative stress.^1^^,^^2^ Copper is absorbed through the duodenum and proximal jejunum by the action of high affinity copper uptake protein 1 (CTR1).^3^^,^^4^ The copper-transporting ATPase 1 (ATP7A) transporter is localized in the basolateral membrane and is responsible for the release of copper in the portal circulation, where copper is loosely bound to soluble chaperones, including albumin, histidines, and macroglobulines.^5^^,^^6^ From the portal vein, copper reaches the liver, where it is chaperoned into hepatocyte subcellular compartments, in particular mitochondria, trans-Golgi network, and the nucleus. Ultimately, copper will be tightly bound to ceruloplasmin in a process facilitated by transmembrane copper transporter ATP7B. ATP7B is also responsible for excreting excess copper via the biliary tract. ATP7B is expressed in multiple organs, most importantly the liver and the brain, but it is also highly expressed in the intestinal epithelial cells (IECs) of the duodenum and jejunum.^7^ ATP7B pathogenic variants and related transporter dysfunction are the cause of Wilson disease (WD), a rare autosomal recessive disease due to copper accumulation in the liver and in the brain. WD is a complex systemic metabolic disease where the clinical presentation is varied, often with unpredictable outcomes and response to treatment. The clinical presentation commonly includes hepatic steatosis^8^^,^^9^ as well as overlooked signs and symptoms derived from the gastrointestinal tract, including changes in bowel habits and abdominal pain.^10^^,^^11^ In addition, given the common hepatic and neurologic presentations, it is plausible the gut-liver-brain axis plays a role in the development of neuropsychiatric presentations. There is evidence of reduced and altered gut microbiota diversity in patients with WD compared with healthy subjects.^12^ These findings were confirmed and correlated with the lipid profile in the liver and plasma of genetic mouse models of hepatic copper accumulation.^13^ A study on organoids derived from IECs isolated from *Atp7b* global knockout mice identified lipid droplet accumulation in the IECs and evidence of aberrant processing of lipids in the same cells, findings that could be attributed to ATP7B dysfunction in the intestinal epithelium.^7^ Gut microbiota also affects the lipid profile with consequences on liver pathology and regeneration.^14^ An extensive study on mouse and rat models of WD and *in vitro* on human Caco-2 cells demonstrated increased lymphocyte infiltration in the small intestine and increased intestinal permeability with altered energy and lipid metabolism in intestinal homogenates.^15^ However, it is unknown if metabolic changes in WD are related directly to intestinal ATP7B deficiency or if they are a consequence of hepatic copper accumulation and liver disease. To understand the effects of IEC ATP7B dysfunction on the intestine and liver in the absence of hepatic copper accumulation, a mouse model with a targeted intestine-specific ATP7B knockout (*Atp7b*^ΔIEC^) was generated. This is important because copper is absorbed in the intestine; signs and symptoms of WD are also relevant to the gastrointestinal tract; and established anti-copper medications exert their action at the intestine level. In particular, zinc salts, which are frequently prescribed in the treatment of WD, act by inducing the synthesis of metallothioneins in the IECs, which, in turn, block the intestinal absorption of dietary copper.^16^ Recent studies on copper chelators in humans demonstrated an unexpected mechanism of action with trientine tetrahydrochloride on inhibiting copper intestinal absorption.^17^ With these premises, we hypothesized that intestinal ATP7B plays a role in modulating WD metabolic manifestations and liver disease. In the present study, *Atp7b*^ΔIEC^ mice were compared with *Atp7b* global knockout mice on a C57Bl/6 background (*Atp7b*^–/–^) in a time-course study from 9 to 30 weeks of age. By exploring copper quantification, liver and intestine morphology, and liver and intestine RNA sequencing, we were ultimately able to identify pathways specifically affected by extrahepatic ATP7B dysfunction.

## Materials and Methods

### Animal Models

All protocols were approved by the University of California Davis Institutional Animal Care and Use Committee and followed the National Research Council's *Guide for the Care and Use of Laboratory Animals*.^18^

#### *Atp7b*^ΔIEC^ Model Generation

As previously described,^13^ the UC Davis Mouse Biology Program generated *Atp7b*^ΔIEC^ mice using B6.Cg-Tg(Vil1-cre)997Gum/J mice from the Jackson Laboratory (Bar Harbor, ME) and *Atp7b*^Lox/Lox^ mice,^19^ kindly provided by S.L. (Johns Hopkins University, Baltimore, MD). Vil1-cre mice express Cre recombinase in villus and crypt epithelial cells of the intestine under a villin-1 promoter. *Atp7b* gene inactivation occurs by Cre-mediated removal of a 1.6-kb fragment in exon 2. Mice heterozygous for *Atp7b* Lox and hemizygous for Vil1-cre (Lox^+/–^/Cre^+^) were produced and subsequently bred to generate Lox^+/+^/Cre^+^, or *Atp7b*^ΔIEC^, mice. IEC-specific *Atp7b* inactivation was confirmed by gene and protein expression analysis (Supplemental Figure S1).

#### Animal Husbandry and Model Characterization

The *Atp7b*^–/–^ global knockout on a C57Bl/6 background was generated as previously described^19^ and kindly provided by S.L. *Atp7b*^–/–^ and *Atp7b*^ΔIEC^ colonies were bred and maintained on the UC Davis campus in standard, open-top, plastic shoebox cages with Teklad TEK-Fresh bedding (Envigo, Madison, WI) and nesting enrichment material under the following conditions: 20°C to 23°C, 40% to 65% relative humidity, 14 hours light/10 hours dark/light cycle, and *ad libitum* LabDiet 5001 (PMI, St. Louis, MO) and deionized water. Mice were housed two to four per cage. Colonies were maintained by hemizygous (*Atp7b*^ΔIEC^) or heterozygous (*Atp7b*^–/–^) breeding. *Atp7b*^–/–^ and *Atp7b*^ΔIEC^ mice were co-housed with their respective littermate controls and segregated by sex at weaning.

At 9, 16, 24, and 30 weeks of age, male and female *Atp7b*^–/–^ and *Atp7b*^ΔIEC^ mice, and their respective controls, had body weights measured before being anesthetized with isoflurane. Mice were then exsanguinated retro-orbitally into K2EDTA collection tubes (Sarstedt, Nümbrecht, Germany), euthanized by cervical dislocation, and their livers and mesenteric white adipose tissue were weighed and flash-frozen in liquid nitrogen. All samples were collected between 9 am and 11:30 am. Blood samples were centrifuged at 6000 × *g* for 10 minutes, and the plasma was aliquoted. All samples were stored at –80°C until further analysis.

### IEC Isolation

IECs were isolated as previously described.^13^ Briefly, mice were anesthetized with isoflurane, then euthanized by cervical dislocation. A ventral incision site was made in the abdominal cavity, and the small intestine was removed and unfurled. The proximal half of the full small intestine length plus 2 cm was taken and further divided into four segments that were placed in a glass petri dish with ice-cold dissection buffer composed of Hanks’ balanced salt solution without calcium/magnesium (Gibco, Bleiswijk, the Netherlands), 10 mmol/L HEPES (Sigma, St. Louis, MO), and 5% fetal bovine serum (Genesee Scientific, El Cajon, CA). Each segment was flushed with ice-cold dissection buffer, and any remaining pancreas and mesenteric fat were trimmed. Each segment was then cut open longitudinally, cut into approximately 0.5-cm pieces, placed in 40 mL dissociation buffer [Hanks’ balanced salt solution without calcium/magnesium, 10 mmol/L HEPES, 12.5 mmol/L EDTA (Sigma), 5% fetal bovine serum, and 1 mmol/L dithiothreitol (Sigma)], and dissociated with an orbital shaker for 30 minutes at 300 rpm and 37°C. After passing the dissociated suspension through a 70-μm cell strainer and centrifuging, the supernatant was removed and the cell pellet was washed with three rounds of 25-mL ice-cold phosphate-buffered saline (pH 7.2) with 0.5% fetal bovine serum and centrifugation. Live cells were counted and the cell suspension was aliquoted. Aliquots were centrifuged, supernatant was removed, and cell pellets were flash-frozen and stored at –80°C. All centrifugations were performed for 5 minutes at 300 × *g* and 4°C.

### Liver and Serum Triglycerides and Total Cholesterol

For liver, 110 ± 10 mg of tissue was weighed and homogenized with sodium sulfate, then transferred into a glass tube. Methanol (4 mL) and chloroform (8 mL) were added, and the samples were incubated overnight at 4°C. The next day, 2.4 mL of 0.7% NaCl solution was added to separate the chloroform phase, and samples were incubated overnight at 4°C. The supernatant layer was aspirated, and a 5-mL sample of the chloroform was evaporated under nitrogen gas, then reconstituted with 0.4 mL of isopropanol. The reconstituted sample was assayed with Infinity reagents TR22421 and TR13421 from Fisher Diagnostics (Middletown, VA). For serum, 5 μL of sample was assayed in duplicate directly with the Fisher Diagnostics reagents. All chemicals were from Fisher Scientific (Hampton, NH).

### ALT and Ceruloplasmin Activity Levels in Plasma

Samples for alanine transaminase (ALT) were processed by the UC Davis Comparative Pathology Laboratory for analysis. Liver and kidney chemistries were analyzed on a Roche Integra 400+ (Roche Diagnostics, Indianapolis, IN) using Cobas assays by Roche Diagnostics for enzymes, substrates, and specific proteins. Ceruloplasmin ferroxidase activity was assayed using the Ceruloplasmin Colorimetric Activity Kit (Invitrogen, Carlsbad, CA), according to the manufacturer's protocol. Samples were diluted 1:20 in the provided assay buffer, and a ceruloplasmin substrate was added. Plates were incubated at 30°C for 60 minutes, followed by absorbance reading at 560 nm with a Synergy H1 microplate reader (Bio Tek, Winooski, VT).

### RNA Extraction and Gene Expression Analysis

RNA isolation, cDNA generation, and real-time quantitative PCR were performed as previously described.^20^ Briefly, RNA was isolated from 25 mg liver and 1 × 10^7^ IECs using the AllPrep RNA/DNA Mini kit (Qiagen, Valencia, CA), according to the manufacturer's instructions. Qiagen QIAshredder columns were used to homogenize IEC samples; liver samples were homogenized using a pellet pestle motor for 20 seconds (Fisher Scientific). RNA purity, concentration, and integrity were assessed by SpectraDrop Micro-Volume Microplate and Spectramax i3x plate reader (Molecular Devices, San Jose, CA) and agarose gel electrophoresis. The Superscript III First-strand Synthesis System (Invitrogen, Carlsbad, CA) was used to reverse transcribe 5 μg RNA into cDNA. Quantitative PCR was run on a ViiA 7 Real-Time PCR System (Applied Biosystems, Foster City, CA) using SYBR Green master mix (Applied Biosystems) and a 1:25 cDNA dilution plated in triplicate along with a no-template control. *Gapdh* was used as a reference gene in IECs and *Ndufs3* in liver for *Atp7b* model validation.

### RNA-Sequencing Analysis

RNA-sequencing library production, sequencing, and analysis were performed by Novogene Corp., Inc. (Sacramento, CA), as previously described.^21^ Briefly, the RNA Nano 6000 Assay Kit and Agilent Bioanalyzer 2100 system (Agilent Technologies, Inc., Santa Clara, CA) were used to assess RNA integrity and quantity. Sequencing libraries were generated using the NEBNext Ultra RNA Library Prep Kit for Illumina (New England Biolabs, Ipswich, MA), followed by size selection, amplification, and purification. Library preparations were sequenced on a NovaSeq 6000 S4 platform (Illumina, Inc., San Diego, CA), generating 150-bp paired-end reads. Raw reads were cleaned using fastp version 0.20.0 (*https://github.com/OpenGene/fastp*), then aligned to GRCm38 mouse reference genome with Spliced Transcripts Alignment to a Reference software version 2.6.1d (*https://github.com/alexdobin/STAR*). Mapped reads were counted for each gene with FeatureCounts version 1.5.0-p3 (*https://bioconductor.org/packages/release/bioc/html/Rsubread.html*), and differential expression analysis of two conditions was performed using edgeR version 3.22.5 (*https://bioconductor.org/packages/release/bioc/html/edgeR.html*). *P* values were adjusted using the Benjamini and Hochberg method, and ClusterProfiler R version 4.6.2 (*https://bioconductor.org/packages/release/bioc/html/clusterProfiler.html*) was used to test the statistical enrichment of differentially expressed genes in Kyoto Encyclopedia of Genes and Genomes and Reactome pathways. Pathways with *P* < 0.05 were considered significant. All sequencing data have been deposited in the National Center for Biotechnology Information's BioSample database with BioProject identifier PRJNA961737 (*https://www.ncbi.nlm.nih.gov/bioproject/961737*, last accessed September 10, 2025) and Gene Expression Omnibus submission GSE279388 (*https://www.ncbi.nlm.nih.gov/geo/query/acc.cgi?acc=GSE279388*, last accessed September 10, 2025).

### Lysate Preparation and Western Blot Analysis

Frozen tissue was homogenized in radioimmunoprecipitation assay buffer (150 mmol/L NaCl, 1% Nonidet P-40, 0.5% sodium deoxycholate, 0.1% SDS, and 50 mmol/L Tris-Cl, pH 7.4) with Pierce EDTA-free Protease Inhibitor Tablets (ThermoFisher Scientific, Waltham, MA) and PhosStop phosphatase inhibitor (Roche, Mannheim, Germany) using a pellet pestle motor for 30 seconds (Fisher Scientific). Lysates were cleared by centrifugation at 15,000 × *g*, 4°C, for 30 minutes. Lysates were frozen at –20°C before protein quantification using a Pierce BCA Protein Assay Kit (ThermoFisher Scientific). Protein (40 μg) was prepared with NuPage LDS sample buffer (Invitrogen, Waltham, MA), according to the manufacturer's protocols, without heating and was loaded into a 4% to 12% Bolt Bis-Tris Plus protein gel (Invitrogen, Waltham, MA). Gels were run in 2-(N-morpholino)ethanesulfonic acid (MES) buffer at 100 V for 1 hour and transferred on a low-fluorescence polyvinylidene difluoride membrane using a Trans-Blot Turbo RTA Mini 0.45-μm LF PVDF Transfer Kit (Bio-Rad, Hercules, CA). Membranes were blocked with 5% Blotting-grade Blocker (Bio-Rad) in Tris-buffered saline with Tween 20 (TBST) for 1 hour at room temperature. Membranes were washed 3 × 5 minutes with TBST at room temperature and incubated with primary antibody in 5% bovine serum albumin + TBST overnight at 4°C. Membranes were washed 3 × 5 minutes with TBST at room temperature and blotted with secondary antibody in 5% Blotting-grade Blocker in TBST for 1 hour at room temperature. Membranes were washed 3 × 5 minutes with TBST at room temperature before imaging on a ChemiDoc MP Imager (Bio-Rad). Primary antibodies used include anti-ATP7B (1:1000; Abcam, Waltham, MA) and anti–β-actin (1:5000; Santa Cruz Biotechnology, Dallas, TX). Secondary antibodies used were goat anti-rabbit IgG Alexa Fluor Plus 800 (1:2500; Invitrogen, Waltham, MA) and goat anti-mouse IgG Alexa Fluor Plus 555 (1:2500; Invitrogen, Waltham, MA). Images were processed, and densitometry was analyzed using Image Lab software version 6.1.0 build 7 (Bio-Rad).

### Copper Quantification

Liver samples were digested in 4:1 trace metals grade concentrated nitric acid (Fisher Scientific) and hydrogen peroxide in a 65°C water bath, then diluted to 10 mL with milli-Q water. Quantitative standards were made using a custom standard (Inorganic Ventures, Christiansburg, VA) diluted to generate a 100- and a 2000-ng/g mixed element standard in 10.0% nitric acid (v/v). A calibration blank of 10.0% nitric acid (v/v) was used. Inductively coupled plasma mass spectrometry was performed on a Thermo iCapQ ICP-MS (Thermo Fisher Scientific) equipped with an ESI SC-2DX PrepFAST autosampler (Elemental Scientific Inc., Omaha, NE). A mixed element solution (IV-ICPMS-71D; Inorganic Ventures) was used as an internal standard and added inline using the PrepFAST system. The isotope selected for analysis was 63,65Cu, as well as 45Sc, 89Y, and 115In (chosen as internal standards for data interpolation and machine stability). Data were calculated as μg copper per mg wet liver weight. Instrument performance is optimized daily through autotuning followed by verification via a performance report. Elemental analysis was performed at the Northwestern University Quantitative Bio-element Imaging Center, generously supported by the NIH under grant S10OD020118.

### Histology

All liver and intestine samples were fixed in 10% zinc formalin, then paraffin embedded, sectioned, and stained with hematoxylin and eosin. Slide images were scanned with an Aperio AT2 slide scanner (Leica Biosystems, Deer Park, IL). Slide images were stored and annotated on the UC Davis Aperio eSlide Manager online database hosted by Leica Biosystems. Slide section snapshots were acquired with Aperio ImageScope version 12.4.3.5008 (Leica Biosystems). Sample processing and pathologist evaluation were performed by the UC Davis Center for Genomic Pathology Laboratory.

### Transmission Electron Microscopy

After fixation in 2.5% glutaraldehyde and 2% paraformaldehyde in 0.1 mol/L sodium phosphate buffer, tissues were rinsed twice in 0.1 mol/L sodium phosphate buffer for a total of 30 minutes, then placed in 1% osmium tetroxide in 0.1 mol/L sodium phosphate buffer for 1 hour. Tissues were rinsed 2 × 15 minutes in 0.1 mol/L sodium phosphate buffer, then dehydrated in 50%, 75%, and 95% ethanol (at least 30 minutes each) and finally in 100% ethanol (2 × 20 minutes). Tissues were then placed in propylene oxide (2 × 15 minutes). Samples were pre-infiltrated in half resin/half propylene oxide overnight. The next day, tissues were infiltrated in 100% resin (450 mL dodecenyl succinic anhydride, 250 mL Araldite 6005 epoxy resin, 82.5 mL Epon 812 embedding resin, 12.5 mL dibutyl phthalate, and 450 μL benzyldimethylamine) for 5 hours. Tissues were then embedded with fresh resin and polymerized at 65°C overnight. Embedded tissues were sectioned with a Leica EM UC6 ultramicrotome at a thickness of 90 nm and collected on copper mesh grids. Sections were stained with 4% aqueous uranyl acetate for 20 minutes and for 2 minutes in 0.2% lead citrate in 0.1 N sodium hydroxide. Transmission electron microscopy imaging was done on an FEI Talos L120C at 80 kV using a Thermo Scientific Ceta 16MP camera (Thermo Fisher Scientific). All samples were processed and imaged by the UC Davis Biological Electron Microscopy Facility.

## Results

### Body Weights, Tissue Weight Ratios, and ALT

Male and female *Atp7b*^–/–^ mice showed normal body weight compared with respective wild-type (WT) mice until 30 weeks of age, when both male and female *Atp7b*^–/–^ mice started to present lower body weight compared with WT (Table 1). At 9, 16, 24, and 30 weeks of age, both WT and *Atp7b*^–/–^ female mice weighed less than their male counterparts, as expected. Tissue weights for both liver and mesenteric white adipose tissue (MWAT) (Supplemental Table S1) were normalized by body weight. *Atp7b*^–/–^ female mice had higher liver weight ratios at 16, 24, and 30 weeks of age, whereas the increase did not manifest in male *Atp7b*^–/–^ mice until 24 weeks. A significant decrease in MWAT ratio was observed in *Atp7b*^–/–^ male mice at 30 weeks. Female *Atp7b*^–/–^ mice had a significant decrease in MWAT ratio at 24 weeks. Interestingly, sex differences in MWAT ratio were only seen at 30 weeks, with female WT mice having significantly lower MWAT ratio relative to male WT mice. This difference in 30-week–old WT females may explain the lack of significant MWAT difference between 30-week *Atp7b*^–/–^ and WT female mice. Significant increases in ALT levels were seen in both male and female *Atp7b*^–/–^ mice starting at 16 weeks, relative to WT, and persisted in both sexes through 30 weeks of age (Table 2). Sex-dependent differences in ALT levels were only seen between male and female mice at 24 weeks. Ceruloplasmin activity levels did not show consistent or significant differences except for a significant reduction in 16-week–old *Atp7b*^–/–^ females compared with WT.

**Table 1:** Body Weights and Normalized Liver Weights

Genotype	*n*		Body weight, g		Liver/body weight	
	M	F	Male	Female	Male	Female
9 Weeks						
WT	5	4	23.9 ± 1.6	20.4 ± 0.5^†††^	0.056 ± 0.002	0.049 ± 0.010
*Atp7b*^–/–^	4	5	24.3 ± 1.8	20.2 ± 1.6^†††^	0.053 ± 0.002	0.050 ± 0.007
iWT	4	3	28.0 ± 0.4	20.2 ± 1.1^†††^	0.046 ± 0.003	0.053 ± 0.003
*Atp7b*^ΔIEC^	4	4	23.9 ± 1.4∗∗	19.8 ± 0.1^†††^	0.055 ± 0.007∗	0.057 ± 0.004
16 Weeks						
WT	12	13	29.2 ± 1.8	23.6 ± 1.7^†††^	0.049 ± 0.006	0.045 ± 0.007
*Atp7b*^–/–^	10	11	27.7 ± 1.7	23.0 ± 1.1^†††^	0.053 ± 0.003	0.056 ± 0.011∗
iWT	11	12	29.5 ± 1.7	22.8 ± 1.3^†††^	0.050 ± 0.005	0.048 ± 0.004
*Atp7b*^ΔIEC^	10	14	28.5 ± 1.9	22.7 ± 1.1^†††^	0.048 ± 0.004	0.049 ± 0.004
24 Weeks						
WT	12	10	29.6 ± 2.5	24.3 ± 2.2^†††^	0.049 ± 0.005	0.046 ± 0.007
*Atp7b*^–/–^	12	9	28.1 ± 2.1	23.7 ± 0.8^†††^	0.062 ± 0.007∗∗	0.062 ± 0.017∗
iWT	11	10	32.0 ± 2.4	23.7 ± 1.5^†††^	0.048 ± 0.005	0.043 ± 0.005^††^
*Atp7b*^ΔIEC^	12	10	31.8 ± 1.6	23.5 ± 0.8^†††^	0.048 ± 0.004	0.048 ± 0.010
30 Weeks						
WT	12	13	32.1 ± 2.5	25.4 ± 1.8^†††^	0.045 ± 0.006	0.046 ± 0.005
*Atp7b*^–/–^	10	14	27.2 ± 2.0∗∗∗	22.8 ± 1.5^†††^∗∗∗	0.064 ± 0.009∗∗∗	0.066 ± 0.011∗∗∗
iWT	11	11	32.6 ± 2.8	26.1 ± 2.3^†††^	0.045 ± 0.005	0.004 ± 0.005
*Atp7b*^ΔIEC^	10	11	32.9 ± 2.0	25.9 ± 2.6^†††^	0.044 ± 0.005	0.004 ± 0.004

Values are means ± SD and statistical significance was determined by *t*-test. An asterisk (∗) indicates values are significantly different between a Wilson disease model (*Atp7b*^–/–^ or *Atp7b*^ΔIEC^) and its respective control (WT or iWT) within the same sex (∗*P* < 0.05, ∗∗*P* < 0.01, and ∗∗∗*P* < 0.001). A dagger (†) indicates values are significantly different between sexes within the same genotype (^††^*P* < 0.01, ^†††^*P* < 0.001).

F, female; M, male; *Atp7b*^–/–^, *Atp7b* null global knockout on C57Bl/6 background; *Atp7b*^ΔIEC^, intestine epithelial cell–specific knockout on C57Bl/6 background; iWT, wild-type controls (Lox^+/+^:Cre^−^) for *Atp7b*^ΔIEC^; WT, wild-type controls (*Atp7b*^+/+^) for *Atp7b*^–/–^.

**Table 2:** Alanine Transaminase and Ceruloplasmin Activity Levels

Genotype	*n*		Alanine transaminase, U/L		*n*		Ceruloplasmin activity, mU/mL	
	M	F	M	F	M	F	M	F
9 Weeks								
WT	5	4	40.2 ± 22.7	31.9 ± 10.9				
*Atp7b*^–/–^	4	5	28.5 ± 5.0	35.1 ± 14.9				
iWT	4	3	41.3 ± 5.4	30.6 ± 9.2				
*Atp7b*^ΔIEC^	4	4	31.4 ± 9.4	29.7 ± 11.3				
16 Weeks								
WT	12	13	80.2 ± 69.7	68.2 ± 60.8	4	4	8256 ± 1406	9500 ± 2418
*Atp7b*^–/–^	10	11	216.0 ± 83.7∗	164.6 ± 51.2∗	4	4	9658 ± 2353	6342 ± 1660∗
iWT	11	12	67.9 ± 36.7	95.7 ± 44.2	4	4	9593 ± 2864	10,059 ± 1689
*Atp7b*^ΔIEC^	10	14	63.3 ± 11.0	46.9 ± 26.8	4	4	8795 ± 2471	10,242 ± 3075
24 Weeks								
WT	12	10	131.0 ± 51.3	50.8 ± 19.9^†^				
*Atp7b*^–/–^	12	9	348.9 ± 139.4∗	268.5 ± 147.7∗				
iWT	11	10	83.9 ± 34.2	58.9 ± 44.4				
*Atp7b*^ΔIEC^	12	10	106.5 ± 44.0	68.5 ± 37.9				
30 Weeks								
WT	12	13	62.6 ± 25.3	80.3 ± 18.0	4	4	9307 ± 3230	10,035 ± 2902
*Atp7b*^–/–^	10	14	421.4 ± 93.9∗∗	363.4 ± 126.9∗∗	4	4	9356 ± 2963	11,450 ± 980
iWT	11	11	43.1 ± 24.1	29.2 ± 7.9	4	4	8584 ± 1955	7721 ± 2148
*Atp7b*^ΔIEC^	10	11	58.6 ± 49.8	29.5 ± 4.5	4	4	9848 ± 3515	9644 ± 2512

Values are means ± SD and statistical significance was determined by *t*-test. An asterisk (∗) indicates values are significantly different between a Wilson disease model (*Atp7b*^–/–^ or *Atp7b*^ΔIEC^) and its respective control (WT or iWT) within the same sex (∗*P* < 0.05, ∗∗*P* < 0.01). A dagger (†) indicates values are significantly different between sexes within the same genotype (^†^*P* < 0.05).

F, female; M, male; *Atp7b*^–/–^, *Atp7b* global knockout on C57Bl/6 background; *Atp7b*^ΔIEC^, intestine epithelial cell–specific knockout on C57Bl/6 background; iWT, wild-type controls (Lox^+/+^:Cre^−^) for *Atp7b*^ΔIEC^; WT, wild-type controls (*Atp7b*^+/+^) for *Atp7b*^–/–^.

In contrast to the *Atp7b*^–/–^ mice, *Atp7b*^ΔIEC^ mice did not show significant changes in body weight or liver weight compared with their respective iWT [wild-type controls (Lox^+/+^:Cre^−^) for *Atp7b*^ΔIEC^] (Supplemental Table S1). Male *Atp7b*^ΔIEC^ mice presented a significantly lower body weight only at 9 weeks, compared with iWT mice. This difference was likely due to higher-than-average body weight in iWT mice rather than a failure for *Atp7b*^ΔIEC^ mice to gain weight, which was further confirmed by a lack of difference in weight at later time points. As expected, *Atp7b*^ΔIEC^ and iWT female mice had lower body weight than males at 9, 16, 24, and 30 weeks. Differences in MWAT ratio were sex dependent; female iWT mice had significantly decreased values at 16 weeks compared with male iWT mice, as did female *Atp7b*^ΔIEC^ and iWT mice at 24 weeks compared with their respective male counterparts. Additionally, the increase in serum ALT levels seen in *Atp7b*^–/–^ mice did not occur in *Atp7b*^ΔIEC^ mice.

### Copper Quantification

**Figure 1:**
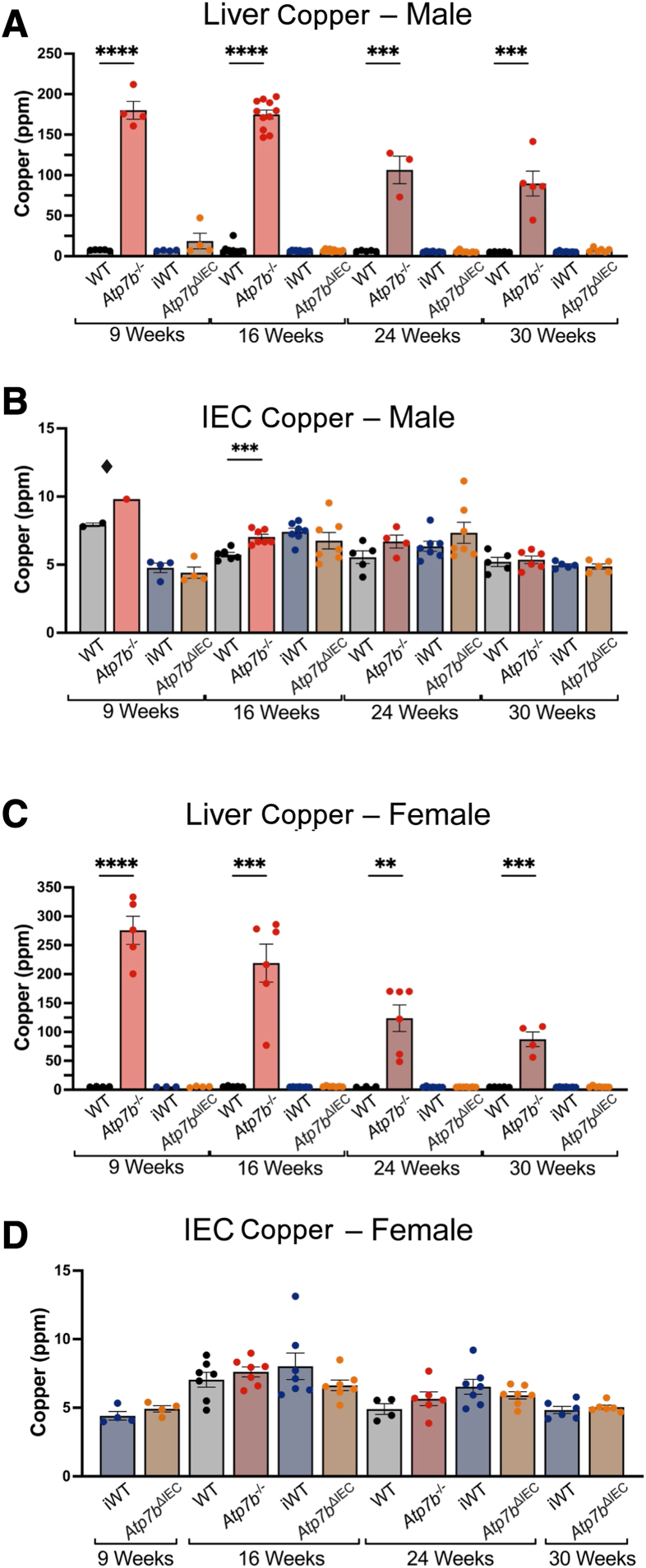
Copper quantification. Liver and intestinal epithelial cell (IEC) copper in male (**A** and **B**) and female (**C** and **D**) *Atp7b*^–/–^ and *Atp7b*^ΔIEC^ mice at 9, 16, 24, and 30 weeks of age. Statistical significance was determined by *t*-test. ^◆^Because of the small sample size, 9-week *Atp7b*^–/–^ and WT IEC copper are presented with sexes combined. Values are means ± SEM (**A**–**D**). (*n*): Liver male *Atp7b*^–/–^ 9 weeks (w) (4), 16 w (11), 24 w (3), 30 w (5); liver male WT 9 w (5), 16 w (12), 24 w (5), 30 w (5); liver male *Atp7b*^ΔIEC^ 9 w (4), 16 w (10), 24 w (7), 30 w (6); liver male iWT 9 w (4), 16 w (11), 24 w (7), 30 w (7); liver female *Atp7b*^–/–^ 9 w (5), 16 w (6), 24 w (6), 30 w (4); liver female WT 9 w (5), 16 w (5), 24 w (3), 30 w (5); liver female *Atp7b*^ΔIEC^ 9 w (3), 16 w (7), 24 w (7), 30 w (7); liver female iWT 9 w (7), 16 w (7), 24 w (7), 30 w (7); IECs male *Atp7b*^–/–^ 9 w (1), 16 w (7), 24 w (4), 30 w (6); IECs male WT 9 w (2), 16 w (6), 24 w (5), 30 w (5); IECs male *Atp7b*^ΔIEC^ 9 w (4), 16 w (7), 24 w (7), 30 w (5); IECs male iWT 9 w (5), 16 w (7), 24 w (7), 30 w (5); IECs female *Atp7b*^–/–^ 16 w (7), 24 w (6); IECs female WT 16 w (7), 24 w (4); IECs female *Atp7b*^ΔIEC^ 9 w (4), 16 w (7), 24 w (7), 30 w (6); IECs female iWT 9 w (4), 16 w (7), 24 w (7), 30 w (6). ∗∗*P* < 0.01, ∗∗∗*P* < 0.001, and ∗∗∗∗*P* < 0.0001. *Atp7b*^–/–^, *Atp7b* global knockout on C57Bl/6 background; *Atp7b*^ΔIEC^, intestine epithelial cell–specific knockout on C57Bl/6 background; iWT, wild-type controls (Lox^+/+^:Cre^−^) for *Atp7b*^ΔIEC^; WT, wild-type controls (*Atp7b*^+/+^) for *Atp7b*^–/–^.

Inductively coupled plasma mass spectrometry was used for copper analysis in both liver and IECs of both mouse models. In agreement with previous studies,^22^ hepatic copper accumulation was seen in both male and female *Atp7b*^–/–^ mice relative to WT at all times points, with copper levels 15- to 25-fold higher in *Atp7b*^–/–^ mice (Figure 1, A and C). Hepatic copper accumulation was highest at 9 weeks and remained at least 15 times higher in *Atp7b*^–/–^ mice compared with WT, despite a progressive reduction over time. Copper levels were not different in the liver of *Atp7b*^ΔIEC^ mice or the IECs of either *Atp7b*^–/–^ or *Atp7b*^ΔIEC^ mice, relative to their respective controls (Figure 1, B and D).

### Liver and Serum Total Cholesterol and Triglycerides

**Figure 2:**
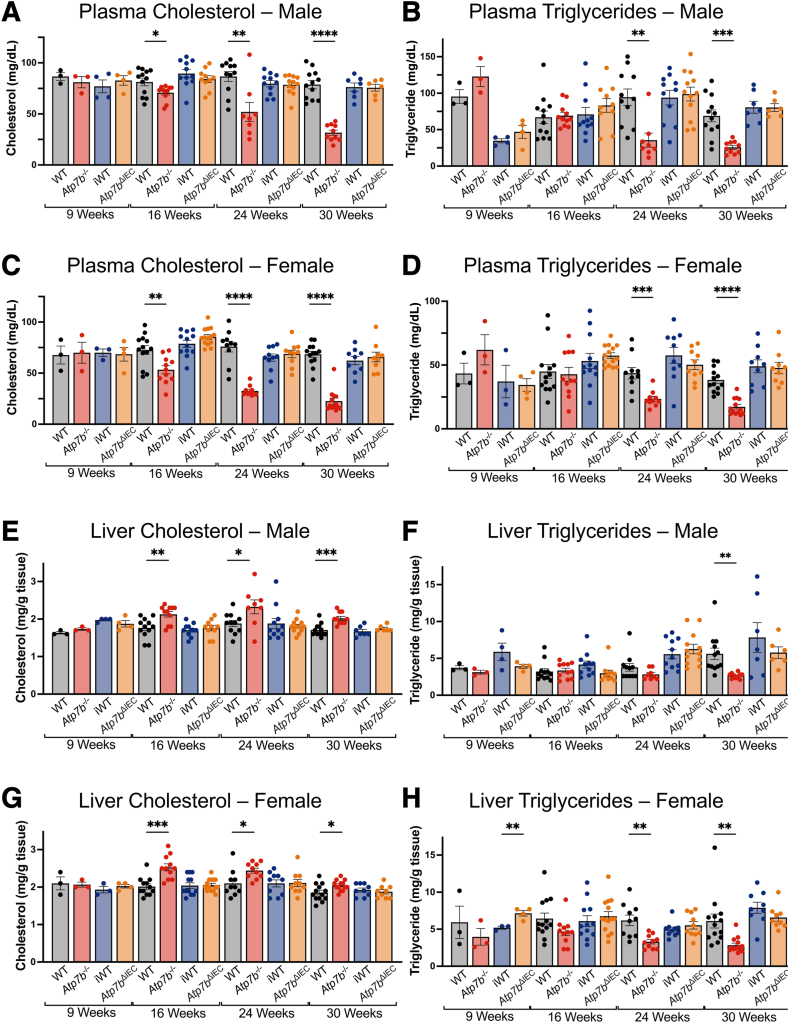
Cholesterol and triglycerides. Plasma cholesterol (**A** and **C**), plasma triglycerides (**B** and **D**), liver cholesterol (**E** and **G**), and liver triglycerides (**F** and **H**) in *Atp7b*^–/–^ and *Atp7b*^ΔIEC^ mice. Statistical significance was determined by *t*-test. Values are means ± SEM (**A**–**H**). (*n*): Plasma male *Atp7b*^–/–^ 9 weeks (w) (3), 16 w (11), 24 w (8), 30 w (10); plasma male WT 9 w (3), 16 w (12), 24 w (11), 30 w (12); plasma male *Atp7b*^ΔIEC^ 9 w (4), 16 w (10), 24 w (12), 30 w (6); plasma male iWT 9 w (4), 16 w (11), 24 w (11), 30 w (7); plasma female *Atp7b*^–/–^ 9 w (3), 16 w (11), 24 w (10), 30 w (13); plasma female WT 9 w (3), 16 w (13), 24 w (10), 30 w (13); plasma female *Atp7b*^ΔIEC^: 9 w (4), 16 w (14), 24 w (10), 30 w (9); plasma female iWT 9 w (3), 16 w (12), 24 w (10), 30 w (9); liver male *Atp7b*^–/–^ 9 w (3), 16 w (11), 24 w (8), 30 w (10); liver male WT 9 w (3), 16 w (12), 24 w (10), 30 w (13); liver male *Atp7b*^ΔIEC^ 9 w (4), 16 w (10), 24 w (12), 30 w (6); liver male iWT 9 w (4), 16 w (11), 24 w (11), 30 w (7); liver female *Atp7b*^–/–^ 9 w (3), 16 w (11), 24 w (10), 30 w (13); liver female WT 9 w (3), 16 w (12), 24 w (10), 30 w (13); liver female *Atp7b*^ΔIEC^ 9 w (4), 16 w (14), 24 w (10), 30 w (9); liver female iWT 9 w (3), 16 w (12), 24 w (10), 30 w (9). ∗*P* < 0.05, ∗∗*P* < 0.01, ∗∗∗*P* < 0.001, and ∗∗∗∗*P* < 0.0001. *Atp7b*^–/–^, *Atp7b* global knockout on C57Bl/6 background; *Atp7b*^ΔIEC^, intestine epithelial cell–specific knockout on C57Bl/6 background; iWT, wild-type controls (Lox^+/+^:Cre^−^) for *Atp7b*^ΔIEC^; WT, wild-type controls (*Atp7b*^+/+^) for *Atp7b*^–/–^.

Given evidence of altered lipid processing in the intestine of *Atp7b*^–/–^ mice,^7^^,^^13^^,^^15^ serum and liver cholesterol and triglyceride (TG) levels were evaluated. Both serum cholesterol and serum TG levels trended downward over time in *Atp7b*^–/–^ male and female mice (Figure 2, A–D). At 24 and 30 weeks, serum cholesterol and TG levels were significantly decreased in both male and female *Atp7b*^–/–^ mice relative to WT. Serum cholesterol showed up to a threefold decrease between *Atp7b*^–/–^ and WT mice, whereas serum TG showed up to a 2.7-fold decrease. Liver cholesterol levels were statistically increased at 16 and 24 weeks in male and female *Atp7b*^–/–^ mice (Figure 2, E and G). In contrast, liver TG levels were decreased at all time points, with more significant decreases occurring with age progression (Figure 2, F and H). By 30 weeks of age, there was a twofold decrease in liver TG levels in both male and female *Atp7b*^–/–^ mice compared with WT mice.

*Atp7b*^ΔIEC^ mice did not show any differences in serum or liver cholesterol and TG levels in either male or female mice relative to iWT at all time points, except for increased TG at 9 weeks in female *Atp7b*^ΔIEC^ relative to iWT. The increase in TG did not persist at later time points and was not seen in male mice (Figure 2).

### Liver Histology

**Figure 3:**
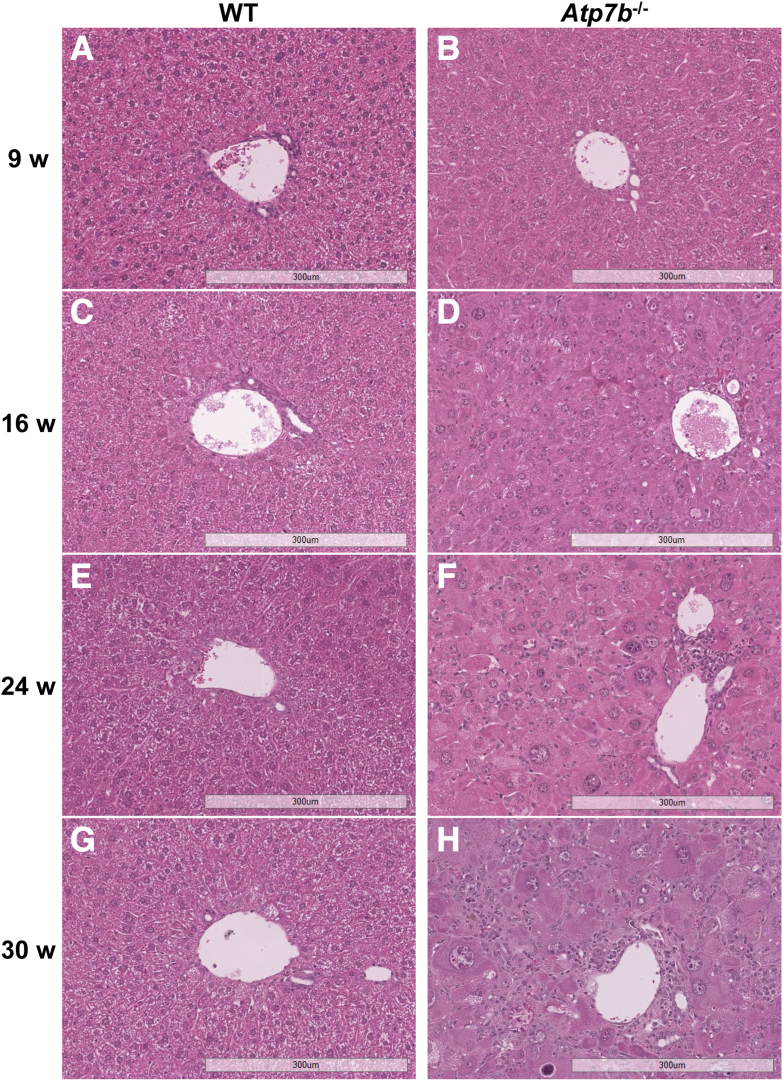
*Atp7b*^–/–^ liver histology. Representative hematoxylin and eosin–stained liver sections from *Atp7b*^–/–^ mice and WT controls at 9 (**A** and **B**), 16 (**C** and **D**), 24 (**E** and **F**), and 30 (**G** and **H**) weeks (w) of age. Liver histology is normal in WT control mice. *Atp7b*^–/–^ mouse livers present progressively enlarged hepatocytes with enlarged and glycogenated hepatocyte nuclei as well as both portal and lobular lymphocytic infiltrates. (*n*): *Atp7b*^–/–-^ 9 w (9), 16 w (27), 24 w (21), 30 w (23); WT 9 w (9), 16 w (30), 24 w (25), 30 w (25). Scale bar = 300 μm (**A**–**H**). Original magnification, ×10 (**A**–**H**). *Atp7b*^–/–^, *Atp7b* global knockout on C57Bl/6 background; WT, wild-type controls (*Atp7b*^+/+^) for *Atp7b*^–/–^.

From 9 to 30 weeks of age, hematoxylin and eosin staining of WT mice showed hepatocytes with a relatively uniform nuclear and hepatocellular size and shape with no inflammation and only mild cytoplasmic glycogenosis by 30 weeks (Figure 3, A, C, E, and G). In contrast, nuclear and hepatocyte enlargement and nuclear glycogenosis were already evident in *Atp7b*^–/–^ mice at 9 weeks of age and continued to increase with age (Figure 3, B, D, F, and H). Although not present at 9 weeks, lobular inflammation began to manifest by 16 weeks. By 30 weeks of age, *Atp7b*^–/–^ livers, on average, presented with moderate lobular and periportal inflammatory infiltrate.

**Figure 4:**
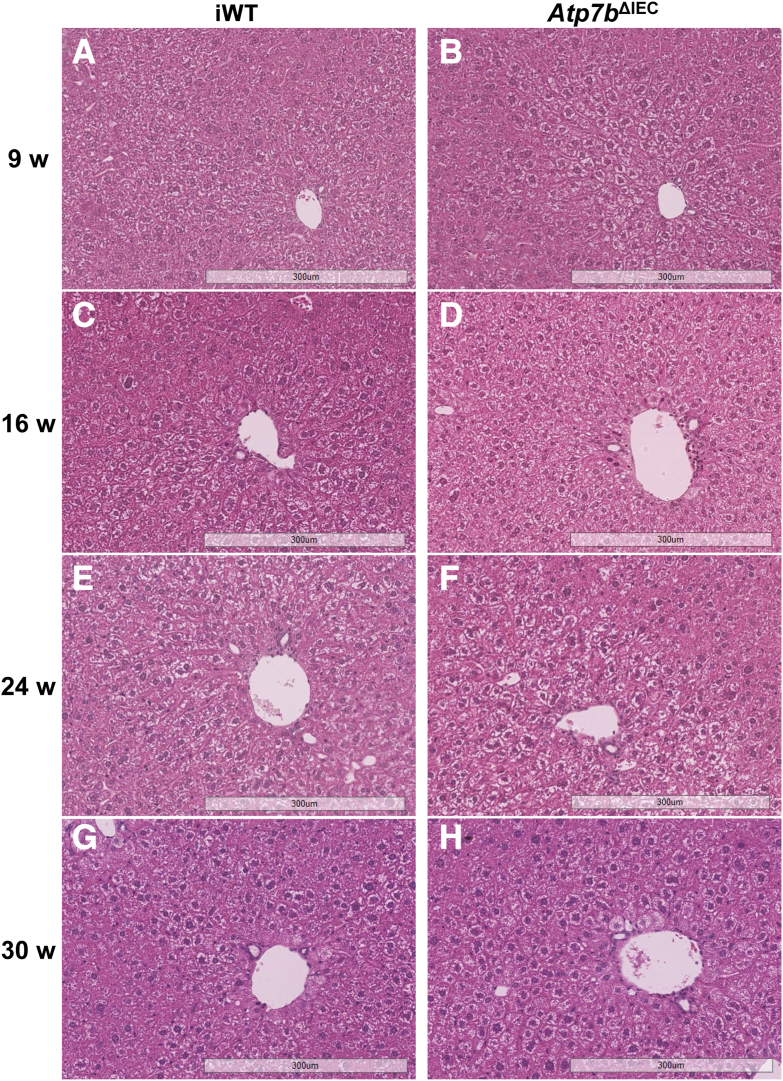
*Atp7b*^ΔIEC^ liver histology. Representative hematoxylin and eosin–stained liver sections from *Atp7b*^ΔIEC^ mice and iWT controls at 9 (**A** and **B**), 16 (**C** and **D**), 24 (**E** and **F**), and 30 (**G** and **H**) weeks (w) of age. Liver histology in iWT control mice and *Atp7b*^ΔIEC^ mouse livers is similar with development of mild glycogenosis over time. (*n*): *Atp7b*^ΔIEC^ 9 w (8), 16 w (28), 24 w (28), 30 w (21); iWT 9 w (7), 16 w (27), 24 w (27), 30 w (22). Scale bar = 300 μm (**A**–**H**). Original magnification, ×10 (**A**–**H**). *Atp7b*^ΔIEC^, intestine epithelial cell–specific knockout on C57Bl/6 background; iWT, wild-type controls (Lox^+/+^:Cre^−^) for *Atp7b*^ΔIEC^.

Unlike the global *Atp7b*^–/–^ model with liver disease, iWT (Figure 4, A, C, E, and G) and *Atp7b*^ΔIEC^ (Figure 4, B, D, F, and H) livers displayed a phenotype similar to WT controls from 9 to 30 weeks of age, regarding nuclear and hepatocellular morphology. However, both *Atp7b*^ΔIEC^ and iWT hepatocytes exhibited a greater degree of cytoplasmic glycogenosis. At 9 weeks, mild glycogenosis was already present, peaking at approximately 24 weeks of age, and then appeared attenuated by 30 weeks.

### Intestine Histology

**Figure 5:**
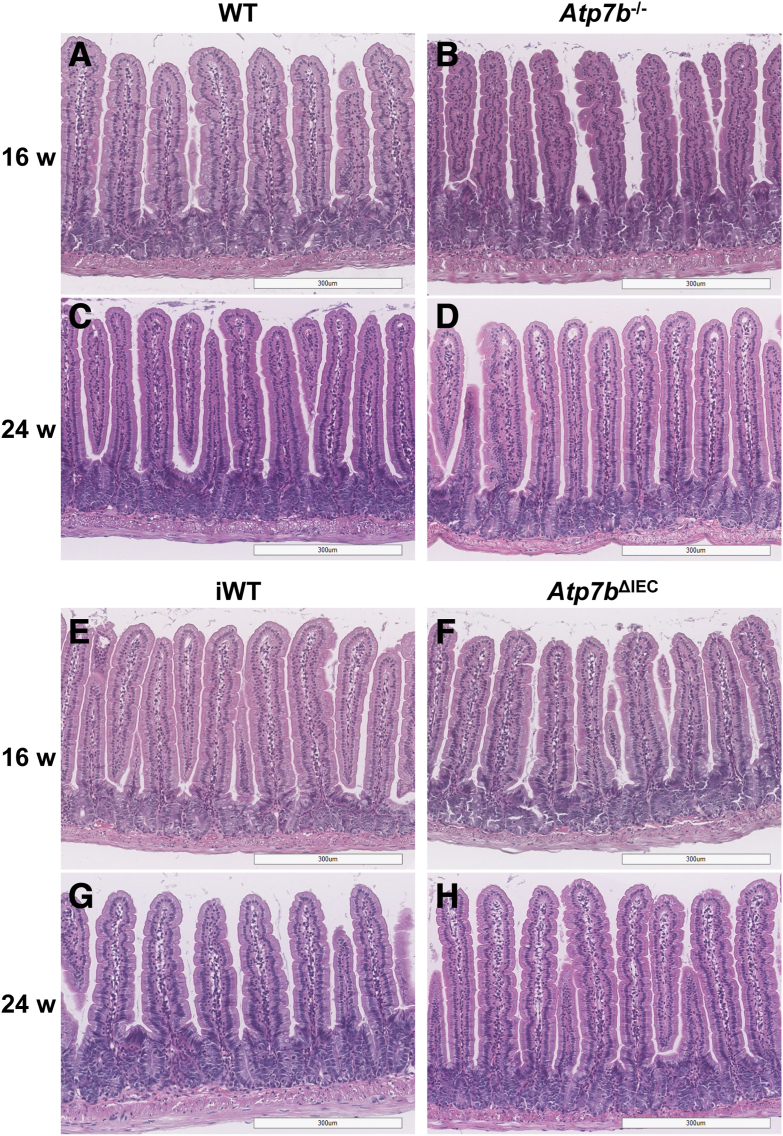
Intestine histology. Representative small intestine hematoxylin and eosin–stained sections from *Atp7b*^–/–^ and *Atp7b*^ΔIEC^ mice. At 16 and 24 weeks (w) of age, intestine histology in *Atp7b*^–/–^ (**B** and **D**) and *Atp7b*^ΔIEC^ (**F** and **H**) mice appears similar to their respective controls, WT (**A** and **C**) and iWT (**E** and **G**). (*n*): *Atp7b*^–/–^ 16 w (4), 24 w (4); WT 16 w (1), 24 w (1); *Atp7b*^ΔIEC^ 16 w (8), 24 w (11); iWT 16 w (4), 24 w (8). Scale bar = 300 μm (**A**–**H**). Original magnification, ×10 (**A**–**H**). *Atp7b*^–/–^, *Atp7b* global knockout on C57Bl/6 background; *Atp7b*^ΔIEC^, intestine epithelial cell–specific knockout on C57Bl/6 background; iWT, wild-type controls (Lox^+/+^:Cre^−^) for *Atp7b*^ΔIEC^; WT, wild-type controls (*Atp7b*^+/+^) for *Atp7b*^–/–^.

Hematoxylin and eosin staining of global *Atp7b*^–/–^ (Figure 5B) and *Atp7b*^ΔIEC^ (Figure 5F) proximal intestine revealed normal pathology at 16 weeks of age compared with respective controls, WT (Figure 5A) and iWT (Figure 5E). The villi were normal, with no signs of intra-epithelial lymphocytes, lamina propria inflammation, or crypt hyperplasia. The epithelial barrier remained intact and the enterocytes and microvilli were well preserved. At 24 weeks of age, slight to mild multifocal lamina propria inflammation with mild distortion of the villous base was observed in all genotypes, WT (Figure 5C) versus *Atp7b*^–/–^ (Figure 5D) and iWT (Figure 5G) versus *Atp7b*^ΔIEC^ (Figure 5H).

#### Transmission Electron Microscopy IEC Mitochondria

**Figure 6:**
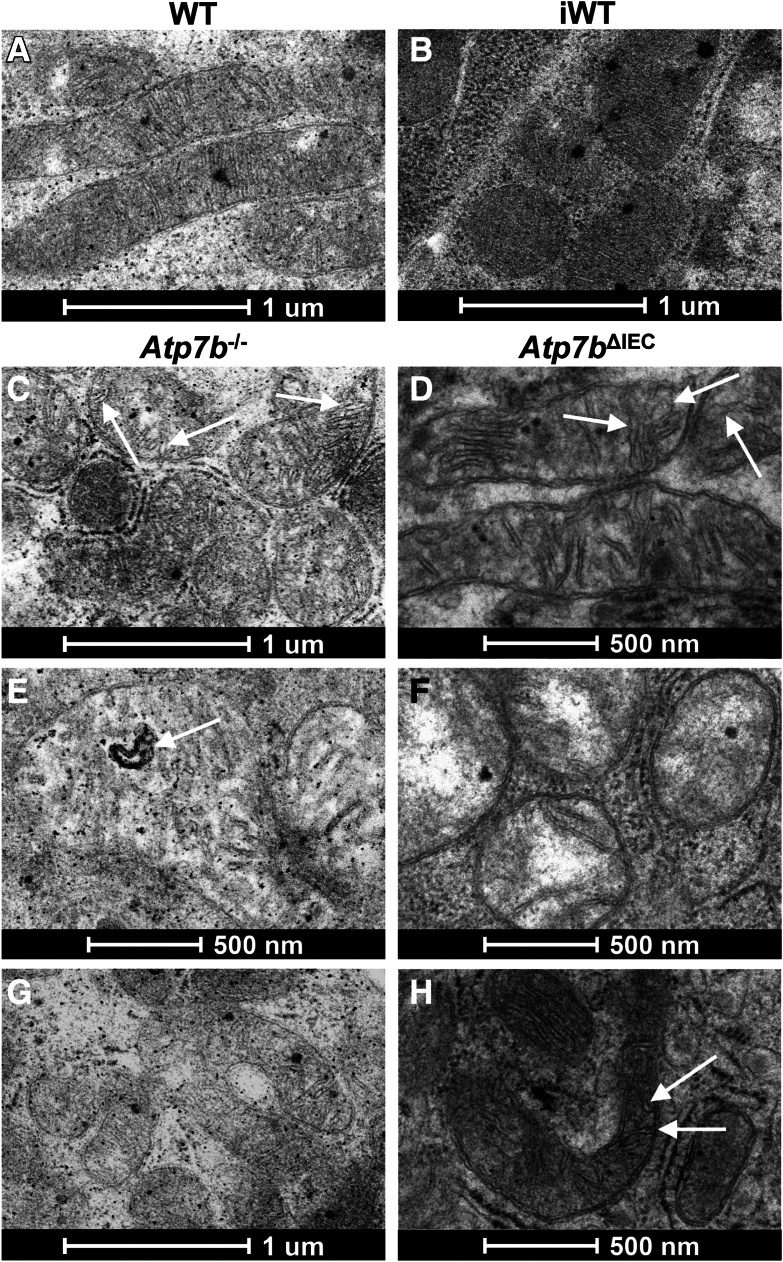
Mitochondrial alterations in *Atp7b*^–/–^ and *Atp7b*^ΔIEC^ mouse intestinal epithelial cells. **A** and **B:** Transmission electron microscopy of normal mitochondria in WT and iWT intestine with electron-dense matrices and organized cristae. **C**–**F:***Atp7b*^–/–^ and *Atp7b*^ΔIEC^ mice frequently exhibit several mitochondrial abnormalities, such as unorganized and depleted cristae, distorted inner and outer membrane, ballooning, and electrolucent matrices. **C**, **D**, **E**, and **H:***Atp7b*^ΔIEC^ also display a significant number of mitochondria with dilated cristae (**white arrows**). **G** and **H:** Occasionally, mitochondria with U-shaped morphology also appear. **A**–**D** and **F**–**H:** At 16 weeks. **E:** At 24 weeks. (*n*): WT = 2; *Atp7b*^–/–^ = 4; iWT = 2; *Atp7b*^ΔIEC^ = 8 (30 to 40 images per *n*). Scale bars: 1 μm (**A**–**C** and **G**); 500 nm (**D**–**F** and **H**). *Atp7b*^–/–^, *Atp7b* global knockout on C57Bl/6 background; *Atp7b*^ΔIEC^, intestine epithelial cell–specific knockout on C57Bl/6 background; iWT, wild-type controls (Lox^+/+^:Cre^−^) for *Atp7b*^ΔIEC^; WT, wild-type controls (*Atp7b*^+/+^) for *Atp7b*^–/–^.

A preliminary survey of duodenal intestine from both *Atp7b*^–/–^ and *Atp7b*^ΔIEC^ mice at 16 and 24 weeks of age showed alterations in mitochondria structure and matrix content. Compared with WT (Figure 6A), *Atp7b*^–/–^ mice had a high frequency of intestinal mitochondria with unorganized or depleted cristae, electron-translucent matrices, and ballooning (Figure 6, C and E). *Atp7b*^–/–^ mice also had occasional mitochondria with vesicular cristae (Figure 6, C and E) and U-shaped morphology (Figure 6G), in contrast to WT mice, which had none.

*Atp7b*^ΔIEC^ mice had similar intestinal mitochondria alterations (Figure 6, D and F) as the *Atp7b*^–/–^ mice when compared with their iWT counterparts (Figure 6B), regarding unorganized or depleted cristae, electron-translucent matrices, and ballooning; however, compromised outer membrane integrity appeared more exacerbated in *Atp7b*^ΔIEC^ mice. Despite ballooning, likely due to outer membrane damage/leakage, *Atp7b*^–/–^ mitochondria maintained a relatively smooth border, whereas *Atp7b*^ΔIEC^ mitochondria, particularly at 16 weeks, had a more distorted border, generating irregular gaps between the inner and outer membranes (Figure 6D). At 16 weeks, *Atp7b*^ΔIEC^ mitochondria also frequently presented with dilated cristae that sometimes appeared vesicular; dilated cristae were also observed at 24 weeks, but dilation was much milder and significantly less frequent (Figure 6, D and H).

#### Transmission Electron Microscopy IEC Barrier

**Figure 7:**
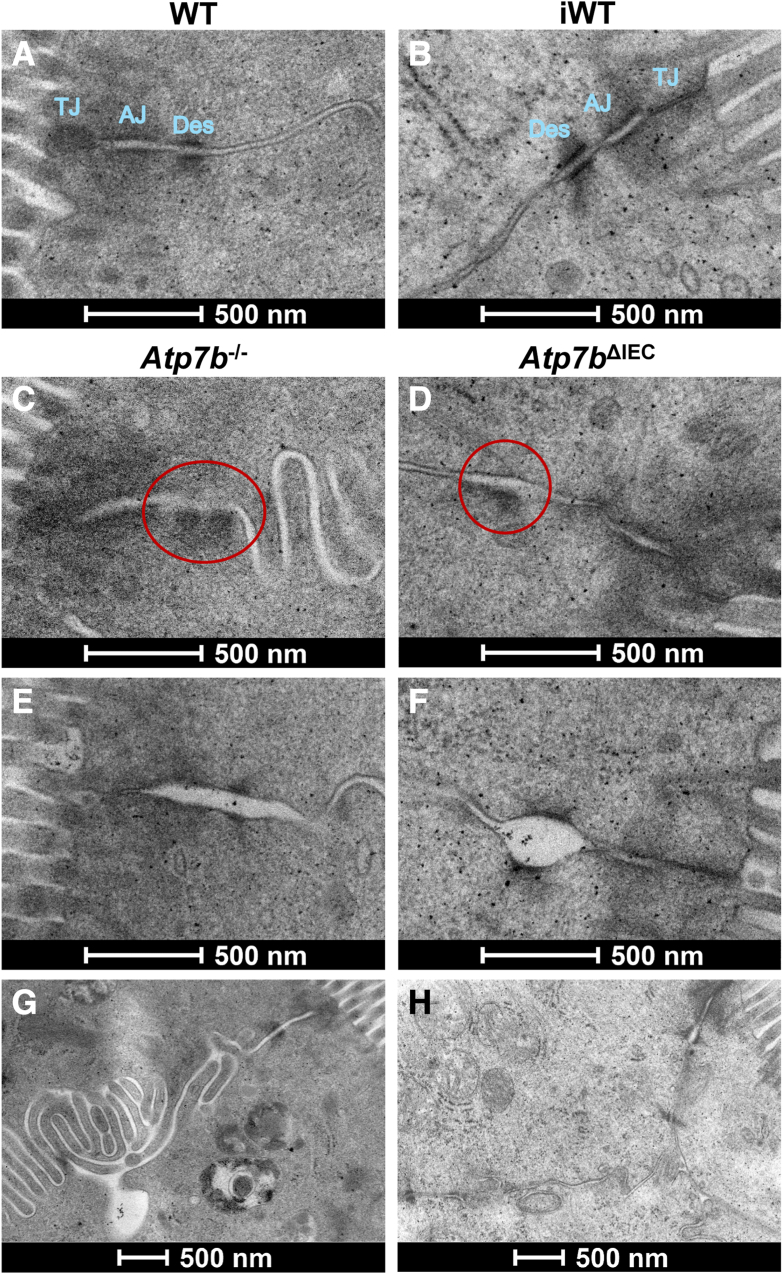
Alterations in the *Atp7b*^–/–^ and *Atp7b*^ΔIEC^ mouse intestinal epithelial cell (IEC) barrier. **A** and **B:** Transmission electron microscopy of normal junctional complexes at the apical surface in WT and iWT IECs. *Atp7b*^–/–^ and *Atp7b*^ΔIEC^ IECs frequently exhibit alterations to parts of the junctional complex and lateral membranes. **C** and **D:** One-sided desmosomes (Des) at the junctional complex (circled) and elsewhere along the lateral membranes are common occurrences in both strains. **E** and **F:** The gap at the adherens junction (AJ) and desmosomes is also often abnormally wide. **G** and **H:***Atp7b*^–/–^ IECs, in particular, form lateral membranes that loop back and forth excessively (**G**) compared with controls and *Atp7b*^ΔIEC^ IECs (**H**). **A**, **C**, and **E:** At 16 weeks. **B**, **D**, and **F**–**H:** At 24 weeks. (*n*): WT = 2; *Atp7b*^–/–^ = 4; iWT = 2; *Atp7b*^ΔIEC^ = 8 (30 to 40 images per *n*). Scale bar = 500 nm (**A**–**H**). *Atp7b*^–/–^, *Atp7b* global knockout on C57Bl/6 background; *Atp7b*^ΔIEC^, intestine epithelial cell–specific knockout on C57Bl/6 background; iWT, wild-type controls (Lox^+/+^:Cre^−^) for *Atp7b*^ΔIEC^; TJ, tight junction; WT, wild-type controls (*Atp7b*^+/+^) for *Atp7b*^–/–^.

In addition to mitochondria, alterations to the IEC apical junctional complex (tight junction, adherens junction, and desmosome) and intercellular membrane adhesion were also examined. Compared with their WT (Figure 7A) or iWT (Figure 7B) controls, both *Atp7b*^–/–^ and *Atp7b*^ΔIEC^ mice exhibited multiple instances of single-sided desmosomes^15^ (Figure 7, C and D), both as part of the junctional complex and other desmosomes along the lateral membranes; in some instances of the junctional complex, there appeared to be no desmosome following the adherens junction. Even when double sided, desmosome proteins are frequently less electrodense and/or are diffuse, appearing like an unorganized protein cloud instead of the more electrodense plaque. At 16 weeks of age, *Atp7b*^–/–^ mice also frequently displayed adherens junctions and desmosomes that were abnormally wide (Figure 7E); this also occurred in *Atp7b*^ΔIEC^ mice, although mostly with just desmosomes and not until 24 weeks (Figure 7F). In *Atp7b*^–/–^ mice, the lateral membranes often looped back and forth excessively with irregular and abnormally wide intercellular adhesion spaces (Figure 7G), compared with WT. *Atp7b*^ΔIEC^ mice, however, rarely had the excessively looping lateral membranes and the intercellular adhesion space between cells was consistent with their iWT counterpart (Figure 7H).

### RNA-Sequencing and Pathway Analysis

RNA sequencing was performed in liver and isolated IECs of both mouse strains at 16 and 30 weeks of age. The two time points were selected on the basis of significant hepatic copper accumulation in *Atp7b*^–/–^ mice and liver histology, which was expected to progress from lack of fibrosis at week 16 to stage 3 to 4 fibrosis with regenerative nodules at week 30.

#### Kyoto Encyclopedia of Genes and Genomes Pathway Analysis

Kyoto Encyclopedia of Genes and Genomes pathway analyses were performed on IECs of *Atp7b*^–/–^ and *Atp7b*^ΔIEC^ mice relative to their respective controls at both 16 and 30 weeks of age. At 16 weeks of age, *Atp7b*^–/–^ mice revealed several differentially regulated pathways, with nucleotide-binding oligomerization domain (NOD)–like receptor signaling being the pathway represented by the highest number of genes (Supplemental Figure S2A and Supplemental Table S2). Genes related to lipid metabolism and mineral absorption, including fat digestion and absorption, were also differentially regulated. Similar to *Atp7b*^–/–^ mice, *Atp7b*^ΔIEC^ mice showed involvement of NOD-like receptor signaling pathway and changes in fat digestion; however, the specific pathways and genes affected were different (Supplemental Figure S2B and Supplemental Table S3). The number of differentially expressed genes was significantly lower in *Atp7b*^ΔIEC^ compared with *Atp7b*^–/–^ mice, with 347 differentially expressed genes in *Atp7b*^ΔIEC^ mice compared with 1576 in *Atp7b*^–/–^ mice. At 30 weeks, the differentially expressed pathways in both *Atp7b*^–/–^ and *Atp7b*^ΔIEC^ mice included nonalcoholic fatty liver disease [now named metabolism-associated steatotic liver disease (MASLD)], oxidative phosphorylation, chemical carcinogenesis via reactive oxygen species, and prion disease pathways (Supplemental Figure S3 and Supplemental Tables S4 and S5). Two genes that appeared as a common thread between these pathways and others listed were mechanistically relevant mitochondrially encoded cytochrome *c* oxidase III (*mt-Co3*) and cytochrome *b* (*mt-Cytb*), both related to mitochondria electron transport.

For Kyoto Encyclopedia of Genes and Genomes pathway analyses in the liver at 16 weeks, changes in cell cycle, fatty acid metabolism, oxidative phosphorylation, and AMP-activated protein kinase (AMPK) signaling were observed in *Atp7b*^–/–^ mice (Supplemental Figure S4A and Supplemental Table S6) and were in agreement with previous studies.^23^ Interestingly, despite normal hepatic *Atp7b* expression levels and lack of hepatic copper accumulation, *Atp7b*^ΔIEC^ mouse livers still presented with changes in gene expression profiles (Supplemental Figure S4B and Supplemental Table S7), although with fewer dysregulated genes and affected pathways compared with *Atp7b*^–/–^ mice. Specifically, affected pathways included DNA replication and AMPK, mitogen-activated protein kinase, and peroxisome proliferator-activated receptor α signaling. By 30 weeks of age, *Atp7b*^–/–^ mice still showed changes in pathways differentially regulated at 16 weeks; however, there was a greater number of differentially expressed genes in those same pathways, likely due to the progression of liver disease (Supplemental Figure S5A and Supplemental Table S8). In contrast, although the dysregulated pathways at 16 weeks remained differentially expressed over time, *Atp7b*^ΔIEC^ mice did not show increases in the numbers of genes affected (Supplemental Figure S5B and Supplemental Table S9).

#### Lipid Metabolism in the IECs of *Atp7b*^–/–^ and *Atp7b*^ΔIEC^ Mice

IEC lipid metabolism pathways were affected at 16 and 30 weeks of age in both *Atp7b*^–/–^ and *Atp7b*^ΔIEC^ mice. Fatty acid digestion and metabolism and linoleic acid metabolism pathways were differentially expressed in both knockout models at 16 weeks of age (Supplemental Figure S6, A and B). Although there was partial overlap in the affected pathways, the specific dysregulated genes were mostly different between the two strains. Compared with respective WT mice, lipid metabolism pathways in *Atp7b*^–/–^ mice showed increased expression of diacylglycerol O-acyltransferase (*Dgat2*), acyl-CoA dehydrogenase long chain (*Acadl*), and fatty acid desaturase 6 (*Fads6)*, all involved in the synthesis of fatty acids (Supplemental Figure S6, C–E). Additionally, *Atp7b*^–/–^ mice exhibited a decrease in acetyl-CoA acetyltransferase 1 (*Acat1*), which encodes for the protein involved in the last step of ketolysis during fat processing. In *Atp7b*^ΔIEC^ mice, the genes driving altered lipid metabolism included down-regulated aldehyde dehydrogenase 7 family member A1 (*Aldh7a1*), which plays a major role in the detoxification of aldehydes, and up-regulated solute carrier family 27 member 2 (*Slc27a2)*, which converts long-chain free fatty acids into fatty acyl-CoA esters (Supplemental Figure S6, F and G). At 30 weeks of age, the trends persisted, with *Atp7b*^–/–^ and *Atp7b*^ΔIEC^ mice showing alterations in lipid metabolism pathways but, again, with different specific genes identified (Supplemental Figure S7, A and B). Increased expression of *Acadl*, stearoyl-CoA desaturase 2 (*Scd2*), and acetyl-CoA acyltransferase 1 (*Acaa1*) was shown in *Atp7b*^–/–^ mice, all of which play a role in lipid biosynthesis (Supplemental Figure S7, C–E). *Atp7b*^ΔIEC^ mice instead presented increased expression of microsomal triglyceride transfer protein (*Mttp*) and monoacylglycerol O-acyltransferase 2 (*Mogat2*), both central in TG synthesis (Supplemental Figure S7, F and G). At 30 weeks, IECs from *Atp7b*^–/–^ mice were compared with heterozygous mice instead of WT mice because of lack of WT mice in the breeding colony.

#### AMPK Signaling in the Liver of *Atp7b*^–/–^ and *Atp7b*^ΔIEC^ Mice

**Figure 8:**
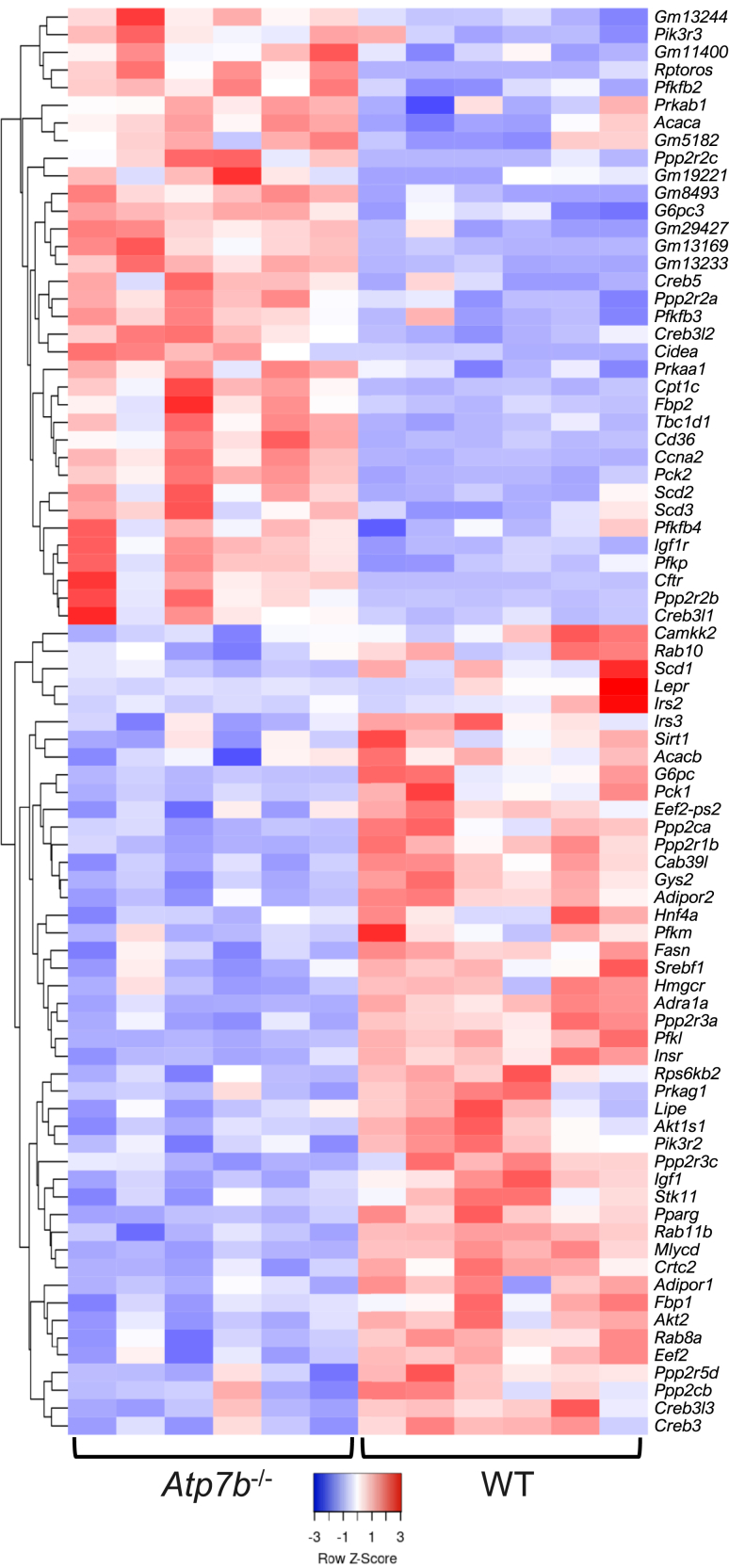
Hepatic AMP-activated protein kinase (AMPK) signaling heat map in *Atp7b*^–/–^ mice at 16 weeks of age. Hierarchical heat map clustering of samples based on Pearson correlation coefficient for genes in the AMPK signaling pathway determined by Kyoto Encyclopedia of Genes and Genomes pathway analysis in the livers of *Atp7b*^–/–^ mice at 16 weeks of age. (*n*): *Atp7b*^–/–^ (6), WT (6). *Atp7b*^–/–^, *Atp7b* null global knockout on C57Bl/6 background; WT, wild-type controls (*Atp7b*^+/+^) for *Atp7b*^–/–^.

**Figure 9:**
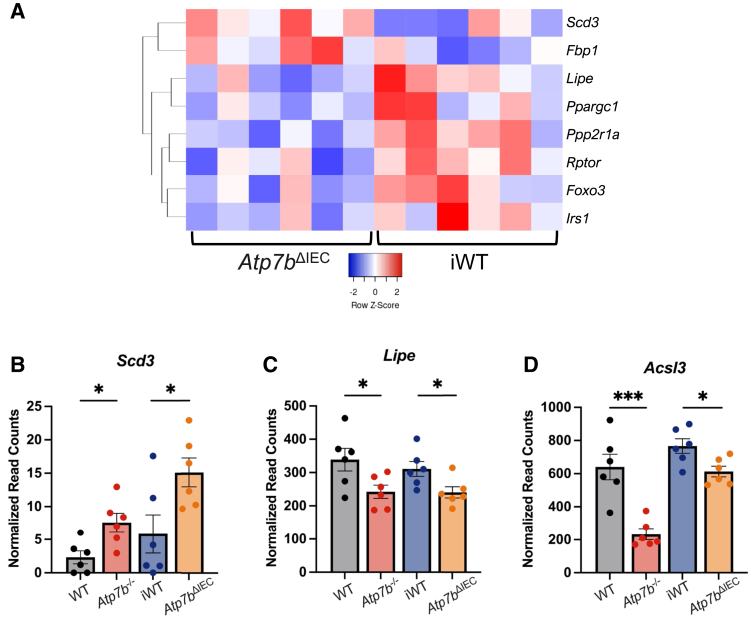
Hepatic AMP-activated protein kinase (AMPK) signaling at 16 weeks of age in *Atp7b*^–/–^ and *Atp7b*^ΔIEC^ mice. **A:** Hierarchical heat map clustering of samples based on Pearson correlation coefficient for genes in the AMPK signaling pathway determined by Kyoto Encyclopedia of Genes and Genomes pathway analysis in the livers of *Atp7b*^ΔIEC^ mice at 16 weeks of age. **B**–**D:** Relative expression of genes for both *Atp7b*^–/–^ and *Atp7b*^ΔIEC^ mice in the AMPK signaling pathway—stearoyl-CoA desaturase 3 (*Scd3*; **B**), lipase E (*Lipe*; **C**), and acyl-CoA synthase long-chain family member 3 (*Acsl3*; **D**). Statistical significance was determined by *t*-test. Values are means ± SEM (**B**–**D**). (*n*): *Atp7b*^–/–^ (6), WT (6), *Atp7b*^ΔIEC^ (6), iWT (6). ∗*P* < 0.05, ∗∗∗*P* < 0.001. *Atp7b*^–/–^, *Atp7b* null global knockout on C57Bl/6 background; *Atp7b*^ΔIEC^, intestine epithelial cell–specific knockout on C57Bl/6 background; iWT, wild-type controls (Lox^+/+^:Cre^−^) for *Atp7b*^ΔIEC^; WT, wild-type controls (*Atp7b*^+/+^) for *Atp7b*^–/–^.

**Figure 10:**
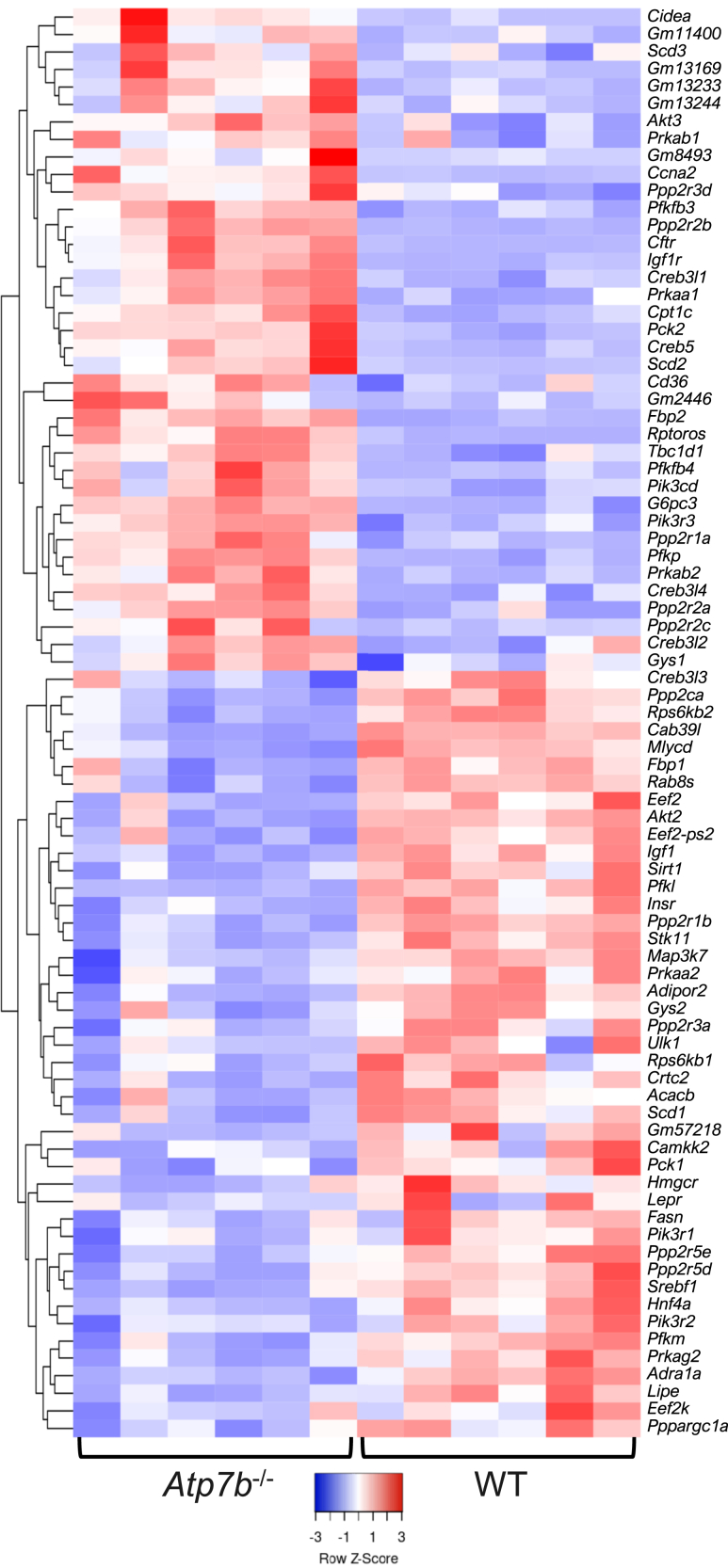
Hepatic AMP-activated protein kinase (AMPK) signaling heat map in *Atp7b*^–/–^ mice at 30 weeks of age. Hierarchical heat map clustering of samples based on Pearson correlation coefficient for genes in the AMPK signaling pathway determined by Kyoto Encyclopedia of Genes and Genomes pathway analysis in the livers of *Atp7b*^–/–^ mice at 30 weeks of age. (*n*): *Atp7b*^–/–^ (6), WT (6). *Atp7b*^–/–^, *Atp7b* null global knockout on C57Bl/6 background; WT, wild-type controls (*Atp7b*^+/+^) for *Atp7b*^–/–^.

**Figure 11:**
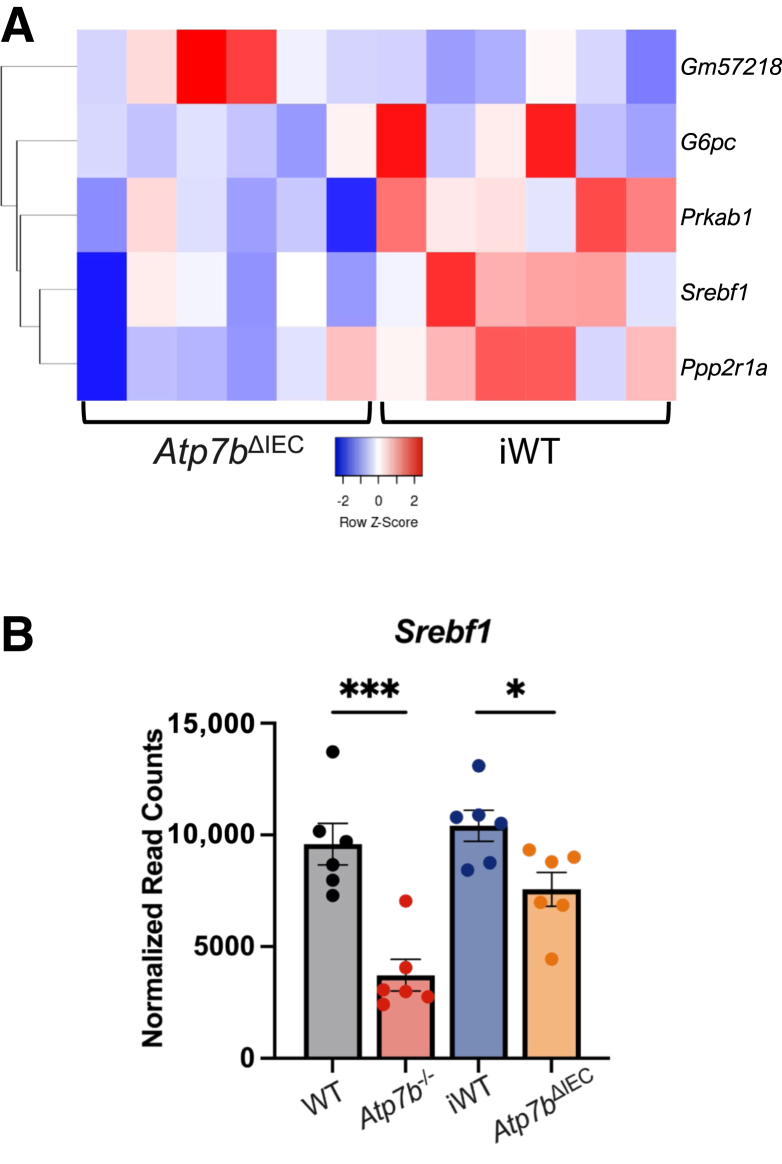
Hepatic AMP-activated protein kinase (AMPK) signaling at 30 weeks of age in *Atp7b*^–/–^ and *Atp7b*^ΔIEC^ mice. **A** and **B:** Hierarchical heat map clustering of samples based on Pearson correlation coefficient for genes in the AMPK signaling pathway determined by Kyoto Encyclopedia of Genes and Genomes pathway analysis in the livers of *Atp7b*^–/–^ (**A**) and *Atp7b*^ΔIEC^ (**B**) mice at 30 weeks of age. **B:** Relative expression of sterol regulatory element-binding transcription factor 1 (*Srebf1*) for both *Atp7b*^–/–^ and *Atp7b*^ΔIEC^ mice. Statistical significance was determined by *t*-test. Values are means ± SEM (**B**). (*n*): *Atp7b*^–/–^ (6), WT (6), *Atp7b*^ΔIEC^ (6), iWT (6). ∗*P* < 0.05, ∗∗∗*P* < 0.001. *Atp7b*^–/–^, *Atp7b* null global knockout on C57Bl/6 background; *Atp7b*^ΔIEC^, intestine epithelial cell–specific knockout on C57Bl/6 background; iWT, wild-type controls (Lox^+/+^:Cre^−^) for *Atp7b*^ΔIEC^; WT, wild-type controls (*Atp7b*^+/+^) for *Atp7b*^–/–^.

In contrast to the IECs, changes in liver pathways were driven by overlapping genes in both *Atp7b*^–/–^ and *Atp7b*^ΔIEC^ mice, although with different numbers of involved genes. Although both knockout models displayed differentially expressed genes in the AMPK signaling pathway at 16 weeks, *Atp7b*^–/–^ mice had 81 genes differentially expressed, whereas *Atp7b*^ΔIEC^ mice presented only 8, with similar trends between the two strains (Figures 8 and 9A). In particular, stearoyl-CoA desaturase 3 (*Scd3*), which enables palmitoyl-CoA 9-desaturase activity and is homologous to human stearoyl-CoA desaturase, and *Scd*, which catalyzes the rate-limiting step in the formation of monounsaturated fatty acids, were up-regulated in both *Atp7b*^–/–^ and *Atp7b*^ΔIEC^ mice (Figure 9B). Additionally, both *Atp7b*^–/–^ and *Atp7b*^ΔIEC^ mice presented decreased expression of lipase hormone (*Lipe*), which enables carboxylic ester hydrolase activity, protein kinase binding activity, and serine hydrolase activity, as well as decreased expression of acyl-CoA synthetase long-chain family member 3 (*Acsl3*), which converts long-chain free fatty acids into fatty acyl-CoA esters in fatty acid metabolism (Figure 9, C and D). The human ortholog of this protein, lipase E, hydrolyzes stored TGs to free fatty acids. At 30 weeks, both knockout models showed persistent changes in the AMPK signaling pathway (Figures 10 and 11A). Sterol regulatory element binding transcription factor 1 (*Srebf1*) transcript levels were reduced in both *Atp7b*^–/–^ and *Atp7b*^ΔIEC^ mice (Figure 11B).

#### Cell Cycle and DNA Replication in the IECs of *Atp7b*^–/–^ and *Atp7b*^ΔIEC^ Mice

Reactome pathway analysis revealed changes in genes related to cell cycle and DNA synthesis and replication in the IECS of *Atp7b*^–/–^ and *Atp7b*^ΔIEC^ mice at 16 (Supplemental Figure S8, A and B) and 30 (Supplemental Figure S8, C and D) weeks of age, and both strains showed an overall down-regulation of genes associated with cell cycle and DNA replication/repair activities. At 16 weeks, *Atp7b*^–/–^ mice showed changes in expression of genes associated with cell cycle (*Cdc20* and *Cdca8*) and centromere genes (*Cenpa*, *Cenpe*, *Cenph*, and *Cenpm*) (Supplemental Table S10), both of which are essential in cell division. In contrast, *Atp7b*^ΔIEC^ mice at 16 weeks had few differentially expressed genes, although they were still histone- and cell cycle–associated genes (Supplemental Table S11). By 30 weeks of age, both mouse strains showed significant changes in cell cycle and DNA replication pathways relative to their respective controls. *Atp7b*^–/–^ mice still exhibited differential expression in genes associated with cell division and the centromere but shifted to genes related specifically to chromatid cohesion (Supplemental Table S12). At 30 weeks, *Atp7b*^ΔIEC^ mice had a significant number of cell cycle and DNA damage response–related genes differentially expressed compared with iWT, including increased expression of several genes encoding proteosome subunits (eg, *Psme1*, *Psmb4*, *Psmb8*, *Psmb10*, and *Psmc5*) (Supplemental Table S13).

## Discussion

This time-course study aimed to characterize a new mouse model with an intestine-specific *Atp7b* knockout. The following key findings were identified: i) *Atp7b* dysfunction causes mitochondrial and junctional abnormalities in the intestine; ii) *Atp7b* dysfunction is associated with differential expression of pathways and genes related to inflammation, mitochondrial function, lipid metabolism, cell cycle, and prion disease in the IECs and in the liver; iii) dysregulated pathways and genes are independent from hepatic copper accumulation or altered liver pathology; and iv) *Atp7b*^–/–^ mice present changes in pathways, including MASLD, that partially overlap between the intestine and the liver. WD is a monogenic disease with systemic manifestations, likely because of the interactions between genetic, epigenetic, and metabolic factors.^21^^,^^24^ However, the role of extrahepatic ATP7B on WD systemic manifestations and liver disease remains unknown. In the absence of copper accumulation and related organ-specific inflammation, it was shown that intestinal ATP7B contributes to the pathophysiology of WD and possibly influences the response to anti-copper treatment. The interaction between the intestine and the liver is crucial in liver diseases and is increasingly recognized in WD. Alcohol-associated liver disease presents increased intestinal permeability with bacterial component translocation, including lipopolysaccharides, which, in turn, induces liver inflammation through increased tumor necrosis factor expression in the liver and thus driving progression of liver disease.^25^^,^^26^ MASLD is similarly associated with intestinal barrier dysfunction, higher levels of circulating bacterial endotoxin,^27^ and changes in the gut microbiota.^28^^,^^29^ Adding to this, Parkinson disease, which is routinely considered in the differential diagnosis of neurologic WD, is also characterized by gut dysbiosis.^30^ WD has liver and intestine morphologic features shared with common liver diseases but also features unique to this rare condition. We observed marked mitochondrial morphologic abnormalities in the IECs of *Atp7b*^–/–^ mice, concomitant with hepatic copper accumulation, and in the IECs of *Atp7b*^ΔIEC^ mice, without copper accumulation. In addition, junctional changes in the intestine of both mouse models were observed. Mitochondrial abnormalities have been previously reported in mouse models of hepatic copper accumulation, mostly focused on the hepatocytes.^31^ Fontes et al^15^ described altered mitochondrial structures in the intestine of two rodent models with WD and Caco-2 ATP7B knockout cells. Rats with hepatic copper accumulation have intestine mitochondria with depleted and unorganized cristae, loss of electron-dense matrices, and detached outer mitochondria membranes. Functionally, in the same study, the authors reported increased intestinal permeability in the 10.13039/100004696WD rat model of acute hepatitis, with *in vivo* findings supported by *in vitro* experiments in Caco-2 cells. Intestinal tissue proteomics analysis identified proteins involved in lipid metabolism and glycolysis as being dysregulated.^15^ Sarode et al^13^ described a specific lipidomic pattern in the liver and plasma of *Atp7b*^ΔIEC^ mice characterized by dysregulated triglyceride, diglyceride, phospholipid, and sphingolipid metabolism, with evidence of abnormal response to a high-fat dietary challenge. In this study, there was evidence of a functional correlation between gut microbiota changes and lipidomic patterns. Intestine involvement in WD pathogenesis was previously explored by Pierson et al,^7^ who showed that organoids derived from *Atp7b*^–/–^ mouse IECs presented impaired lipid processing with mislocalization of apolipoprotein B and loss of chylomicron development. These studies showed intestinal copper metabolism changes present early in the course of liver disease, although there is always significant presence of hepatic copper accumulation. The next important step is understanding to what extent the liver pathology is affected and potentially regulated by these intestinal metabolic changes. In the current study, RNA sequencing of 16-week–old *Atp7b*^–/–^ mice showed IEC changes in fat digestion- and absorption-related genes. This finding was accompanied by misregulation of hepatic fatty acid metabolism and degradation pathways. By 30 weeks of age, the pathways affected in the intestine shift toward chemical carcinogenesis and oxidative phosphorylation, both of which are indicative of mitochondrial damage. This was accompanied by increasing liver disease severity and changes in pathways, including chemical carcinogenesis and nonalcoholic fatty liver disease/MASLD. RNA-sequencing of *Atp7b*^ΔIEC^ mouse IECs revealed similar changes to those seen in *Atp7b*^–/–^ mice, although with a smaller number of involved genes. IECs from 16-week–old *Atp7b*^ΔIEC^ mice showed changes in gene transcripts related to lipid metabolism accompanied by changes in several hepatic signaling pathways, including mitogen-activated protein kinase and AMPK. At 30 weeks of age, the IEC pathways altered in *Atp7b*^ΔIEC^ mice included chemical carcinogenesis and oxidative phosphorylation, similar to *Atp7b*^–/–^ IECs. Pathways affected in the liver of *Atp7b*^ΔIEC^ mice at 30 weeks included AMPK signaling and MASLD, which were seen in *Atp7b*^–/–^ mice at 30 weeks as well. These results suggest the metabolic pathway changes seen in the liver of *Atp7b*^–/–^ mice are at least partially driven by the gut-liver axis, independent from hepatic copper accumulation. A study on *Atp7b*^–/–^ mice previously identified increased hepatic phosphorylated AMPK levels.^23^ Notably, AMPK is a major sensor of oxidative stress,^32^ as induced by copper accumulation, and has been connected to dysregulation of lipid metabolism. The finding of dysregulated AMPK pathway in both the IECs and the liver confirms that the source of aberrant lipid metabolism is actually localized in the gastrointestinal tract. Pathways that need to be mentioned, as they are likely to have pathogenic relevance, include NOD-like receptor signaling pathway, prion disease, cell cycle, and DNA replication. In particular, NOD-like receptor signaling reflects inflammasome activation and could be linked to altered gut microbiota.^33^^,^^34^ Marked dysregulation of this pathway in IECs of both *Atp7b*^–/–^ and *Atp7b*^ΔIEC^ mice at 16 weeks of age was observed. Prion protein has been shown to favor copper toxicity and its suppression is a possible therapeutic target in WD.^35^ Prion protein was identified through genome-wide screening in ATP7B-knockout HepG2 cells and prion disease pathway dysregulation was identified in both IECs and livers of both mouse models.

The other major pathways identified by RNA-sequencing analysis relate to cell cycle and DNA replication. Changes in cell cycle, mitosis, and nuclear division are well described in livers of mouse models and in patients with WD^36^^,^^37^ and the involvement of these pathways in the livers of both mouse models was confirmed. Liver and IEC transcriptome findings were not associated with significant changes in liver pathology, liver enzymes, or cholesterol or triglyceride levels in *Atp7b*^ΔIEC^ mice, suggesting that, ultimately, hepatic copper accumulation is the determinant factor for the progression of liver disease. In addition, there was no evidence of increased intestinal copper in *Atp7b*^ΔIEC^ mice, which is in agreement with previous findings of lack of total copper changes in the intestine of *Atp7b*^–/–^ rats.^15^ The lack of total copper accumulation could be due to redistribution of copper in subcellular compartments. The present study had several strengths, including the time-course assessment of a new mouse model and the fact that RNA-sequencing analyses were conducted specifically on IECs rather than the whole intestine, with consequent detailed characterization of the cellular subtype driving the metabolic changes. Ultimately, this study points to the fact that the pathogenesis of liver disease in WD is affected by ATP7B dysregulation in the intestine as the first site of lipid metabolism and other mechanistically relevant pathways and genes. The clinical implications of these findings are several. Nutrients and dietary components are likely to interact with ATP7B copper transporter not only at the level of dietary copper content but also relative to fat intake. Zinc salts and chelators exert their action at the level of the intestine, and it is possible that *ATP7B* variants interact or interfere in different ways with anti-copper drugs, potentially explaining variable treatment response. Finally, the interactions between intestinal function and permeability, gut microbiota, and intestinal ATP7B dysfunction affect the liver and the brain in WD, more so than any other metabolic liver disease, and are to be seen as new potential therapeutic sites. Treatment options targeted to the intestinal barrier or dietary modifications focused on energy metabolism modulation should be studied in patients with WD, especially when presenting with hepatic steatosis or poor response to conventional copper-lowering strategies. Future studies should include intestine biopsies from patients with WD to explore their morphology and ultrastructure and should plan for dietary interventions based on energy metabolism modulation. The field is rapidly evolving and the present data help with the identification of the intestine as a therapeutic site. In previous studies, human induced pluripotent stem cell–derived hepatocytes have been successfully generated for drug screening or therapeutic purposes in WD.^38^, ^39^, ^40^, ^41^, ^42^ In particular, clustered regularly interspaced short palindromic repeats (CRISPR)–targeted genome editing has successfully edited *ATP7B* variants in pluripotent stem cells derived from patients with WD.^41^ A lentiviral vector-based approach was used to correct ATP7B function in hepatocyte-like cells derived from patients with WD.^38^ It is possible that IECs can be generated to rescue dysfunctional IECs in patients with WD. Ultimately, WD-specific edited hepatocytes and IECs could restore organ-specific ATP7B dysfunction. In conclusion, WD should be managed as a systemic disease with varied clinical presentations and variable response to anti-copper treatments likely due to the occurrence of multiple factors, including extrahepatic ATP7B dysfunction.

## Disclosure Statement

None declared.

## Supplemental Data

Supplemental Figure S1 *Atp7b* model validation. **A:***Atp7b* real-time quantitative PCR for 9-week–old *Atp7b*^ΔIEC^ (Lox^+/+^/Cre^+^) mice and iWT control (Lox^+/+^/Cre^−^), with *Atp7b*^–/–^ mice and WT control as additional references. Results were normalized to *Gapdh* in intestinal epithelial cells (IECs) and *Ndufs3* in liver. *Atp7b* expression is reduced 99% in *Atp7b*^ΔIEC^ IECs compared with iWT. This nearly complete reduction of *Atp7b* expression in the intestine-specific Cre-lox mouse model corresponds to expression levels demonstrated by Muchenditsi et al^19^ in the hepatocyte-specific Cre-lox mouse model, *Atp7b*^ΔHep^. Residual expression detected may be due to either contaminating lymphocytes or incomplete inactivation by Cre recombinase in a hemizygous state. *Atp7b*^–/–^ mice demonstrated no detectable *Atp7b* transcript levels. *Atp7b* levels were not different between *Atp7b*^ΔIEC^ and iWT samples in liver, indicating IEC specificity. **B** and **C:** Representative ATP7B immunoblot of 9-week–old and 16-week–old *Atp7b*^ΔIEC^ and iWT IEC lysates. Blots were normalized to β-actin. ATP7B protein expression reflects gene expression in specificity and quantity; ATP7B is not detectable in *Atp7b*^–/–^ mice but, although dramatically reduced, ATP7B can be detected in *Atp7b*^ΔIEC^ mice as a faint band. **A** and **B:** Previously published by this group.^13^ No changes were made to the figure as published in public access reference.^13^ The figure was published under the Creative Commons Attribution License (CC BY) *https://creativecommons.org/licenses/by-nc-nd/4.0/*. *n* = 8 *Atp7b*^ΔIEC^ (**A**); *n* = 7 iWT control (**A**); *n* = 3 *Atp7b*^–/–^ mice and WT control (**A**); *n* = 5 9-week–old *Atp7b*^ΔIEC^ and iWT IEC lysates (**B**); *n* = 3 16-week–old *Atp7b*^ΔIEC^ and iWT IEC lysates (**B**). *Atp7b*, ATPase copper transporting β; *Atp7b*^–/–^, *Atp7b* global knockout mice on a C57Bl/6 background; *Atp7b*^ΔIEC^ mice, intestine epithelial cell–specific *Atp7b* knockout mice; *Gapdh*, glyceraldehyde 3-phosphate dehydrogenase; iWT, littermate controls (Lox^+/+^/Cre^−^) for *Atp7b*^ΔIEC^ mice; *Ndufs3*, NADH:ubiquinone oxidoreductase core subunit S3; WT, littermate controls (*Atp7b*^+/+^) for *Atp7b*^–/–^ mice.

Supplemental Figure S2 RNA-sequencing (RNA-seq) Kyoto Encyclopedia of Genes and Genomes (KEGG) pathway analysis of intestinal epithelial cells (IECs) at 16 weeks of age. Top 20 RNA-seq KEGG pathways of 16-week–old *Atp7b*^–/–^ versus WT (**A**) and *Atp7b*^ΔIEC^ versus iWT (**B**) IECs. Numbers above bar columns are the number of differentially expressed genes in the pathway. *n* = 6 per genotype (**A** and **B**). *Atp7b*^–/–^, *Atp7b* global knockout on C57Bl/6 background; *Atp7b*^ΔIEC^, intestine epithelial cell–specific knockout on C57Bl/6 background; iWT, wild-type controls (Lox^+/+^:Cre^−^) for *Atp7b*^ΔIEC^; WT, wild-type controls (*Atp7b*^+/+^) for *Atp7b*^–/–^.

Supplemental Figure S3 RNA-sequencing (RNA-seq) Kyoto Encyclopedia of Genes and Genomes (KEGG) pathway analysis of intestinal epithelial cells (IECs) at 30 weeks of age. Top 20 RNA-seq KEGG pathways of 30-week–old *Atp7b*^–/–^ versus heterozygous (Het) (**A**) and *Atp7b*^ΔIEC^ versus iWT (**B**) IECs. Numbers above bar columns are the number of differentially expressed genes in the pathway. *n* = 6 per genotype (**A** and **B**), except for Het, *n* = 5 (**A**). *Atp7b*^–/–^, *Atp7b* global knockout on C57Bl/6 background; *Atp7b*^ΔIEC^, intestine epithelial cell–specific knockout on C57Bl/6 background; iWT, wild-type controls (Lox^+/+^:Cre^−^) for *Atp7b*^ΔIEC^; TCA, tricarboxylic acid cycle; TRP, transient receptor potential; WT, wild-type controls (*Atp7b*^+/+^) for *Atp7b*^–/–^.

Supplemental Figure S4 RNA-sequencing (RNA-seq) Kyoto Encyclopedia of Genes and Genomes (KEGG) pathway analysis of liver at 16 weeks of age. Top 20 RNA-seq KEGG pathways of 16-week–old *Atp7b*^–/–^ versus WT (**A**) and *Atp7b*^ΔIEC^ versus iWT (**B**) liver. Numbers above bar columns are the number of differentially expressed genes in the pathway. *n* = 6 per genotype (**A** and **B**). AMPK, AMP-activated protein kinase; *Atp7b*^–/–^, *Atp7b* global knockout on C57Bl/6 background; *Atp7b*^ΔIEC^, intestine epithelial cell–specific knockout on C57Bl/6 background; ECM, extracellular matrix; iWT, wild-type controls (Lox^+/+^:Cre^−^) for *Atp7b*^ΔIEC^; MAPK, mitogen-activated protein kinase; mTOR, mammalian target of rapamycin; PPAR, peroxisome proliferator-activated receptor; Th17, type 17 helper T cell; WT, wild-type controls (*Atp7b*^+/+^) for *Atp7b*^–/–^.

Supplemental Figure S5 RNA-sequencing (RNA-seq) Kyoto Encyclopedia of Genes and Genomes (KEGG) pathway analysis of liver at 30 weeks of age. Top 20 RNA-seq KEGG pathways of 30-week–old *Atp7b*^–/–^ versus WT (**A**) and *Atp7b*^ΔIEC^ versus iWT (**B**) liver. Numbers above bar columns are the number of differentially expressed genes in the pathway. *n* = 6 per genotype (**A** and **B**). *Atp7b*^–/–^, *Atp7b* global knockout on C57Bl/6 background; *Atp7b*^ΔIEC^, intestine epithelial cell–specific knockout on C57Bl/6 background; ECM, extracellular matrix; iWT, wild-type controls (Lox^+/+^:Cre^−^) for *Atp7b*^ΔIEC^; NOD, nucleotide-binding oligomerization domain; PI3K, phosphatidylinositol 3-kinase; RIG-I, retinoic acid-inducible gene I; WT, wild-type controls (*Atp7b*^+/+^) for *Atp7b*^–/–^.

Supplemental Figure S6 Fatty acid metabolism at 16 weeks of age in the intestinal epithelial cells (IECs) of *Atp7b*^–/–^ and *Atp7b*^ΔIEC^ mice. Hierarchical heat map clustering based on Pearson correlation coefficient for genes in the fatty acid metabolism, fatty acid degradation, and linoleic acid metabolism pathways determined by Kyoto Encyclopedia of Genes and Genomes pathway analysis in the IECs of *Atp7b*^–/–^ (**A**) and *Atp7b*^ΔIEC^ (**B**) mice at 16 weeks of age. Relative gene expression of diacylglycerol O-acyltransferase2 *(Dgat2*; **C**), fatty acid desaturase 6 (*Fads6*; **D**), and acyl-CoA dehydrogenase long chain (*Acadl*; **E**) in *Atp7b*^–/–^ mice. Relative expression of solute carrier family 27 member 2 (*Slc27a2*; **F**) and aldehyde dehydrogenase 7 family member A1 (*Aldh7a1*; **G**) in *Atp7b*^ΔIEC^ mice. Statistical significance was determined by *t*-test. Values are means ± SEM (**C**–**G**). (*n*): *Atp7b*^–^^/–^ (6), WT (6), *Atp7b*^ΔIEC^ (6), iWT (6). ∗*P* < 0.05, ∗∗*P* < 0.01. *Atp7b*^–/–^, *Atp7b* global knockout on C57Bl/6 background; *Atp7b*^ΔIEC^, intestine epithelial cell–specific knockout on C57Bl/6 background; iWT, wild-type controls (Lox^+/+^:Cre^−^) for *Atp7b*^ΔIEC^; WT, wild-type controls (*Atp7b*^+/+^) for *Atp7b*^–/–^.

Supplemental Figure S7 Fatty acid metabolism at 30 weeks of age in the intestinal epithelial cells (IECs) of *Atp7b*^–/–^ and *Atp7b*^ΔIEC^ mice. Hierarchical heat map clustering based on Pearson correlation coefficient for genes in the fatty acid metabolism, fatty acid degradation, and linoleic acid metabolism pathways determined by Kyoto Encyclopedia of Genes and Genomes pathway analysis in the IECs of *Atp7b*^–/–^ (**A**) and *Atp7b*^ΔIEC^ (**B**) mice at 30 weeks of age. Relative gene expression of acyl-CoA dehydrogenase long chain (*Acadl*; **C**), stearoyl-CoA desaturase (*Scd2*; **D**), and acyl-CoA acyltransferase 1(*Acaa1*; **E**) in *Atp7b*^–/–-^ mice. Relative expression of microsomal triglyceride transfer protein (*Mttp*; **F**) and monoacylglycerol O-acyltransferase 2 (*Mogat2*; **G**) in *Atp7b*^ΔIEC^ mice. Statistical significance was determined by *t*-test. Values are means ± SEM (**C**–**G**). ∗*P* < 0.05, ∗∗*P* < 0.01. (*n*): *Atp7b*^–/–^ (6), Heterozygous (Het) (5), *Atp7b*^ΔIEC^ (6), iWT (6). *Atp7b*^–/–^, *Atp7b* global knockout on C57Bl/6 background; *Atp7b*^ΔIEC^, intestine epithelial cell–specific knockout on C57Bl/6 background; iWT, wild-type controls (Lox^+/+^:Cre^−^) for *Atp7b*^ΔIEC^; WT, wild-type controls (*Atp7b*^+/+^) for *Atp7b*^–/–^.

Supplemental Figure S8 Cell cycle, synthesis of DNA, and DNA replication in intestinal epithelial cells (IECs) of *Atp7b*^–/–^ and *Atp7b*^ΔIEC^ mice. Hierarchical heat map clustering based on Pearson correlation coefficient for genes in the cell cycle, synthesis of DNA, and DNA replication pathways determined by Reactome pathway analysis in the IECs of *Atp7b*^–/–^ (**A**) and *Atp7b*^ΔIEC^ (**B**) mice at 16 weeks of age and in the IECs of *Atp7b*^–/–^ (**C**) and *Atp7b*^ΔIEC^ (**D**) mice at 30 weeks of age. (*n*): 16 weeks (w): *Atp7b*^–/–^ (6), WT (6), *Atp7b*^ΔIEC^ (6), iWT (6); 30 w: *Atp7b*^–/–^ (6), Heterozygous (Het) (5), *Atp7b*^ΔIEC^ (6), iWT (6). *Atp7b*^–/–^, *Atp7b* global knockout on C57Bl/6 background; *Atp7b*^ΔIEC^, intestine epithelial cell–specific knockout on C57Bl/6 background; iWT, wild-type controls (Lox^+/+^:Cre^−^) for *Atp7b*^ΔIEC^; WT, wild-type controls (*Atp7b*^+/+^) for *Atp7b*^–/–^.
