# Supplementary figures and images for: Inactivation of Atp7b Copper Transporter in Intestinal Epithelial Cells Is Associated with Altered Lipid Processing and Cell Growth Machinery Independent from Hepatic Copper Accumulation and Severity of Liver Histology
Source: Am J Pathol. 2025 Oct 16;196(2):407–27. doi: 10.1016/j.ajpath.2025.09.015 (PMC12881291; doi:10.1016/j.ajpath.2025.09.015)

**A**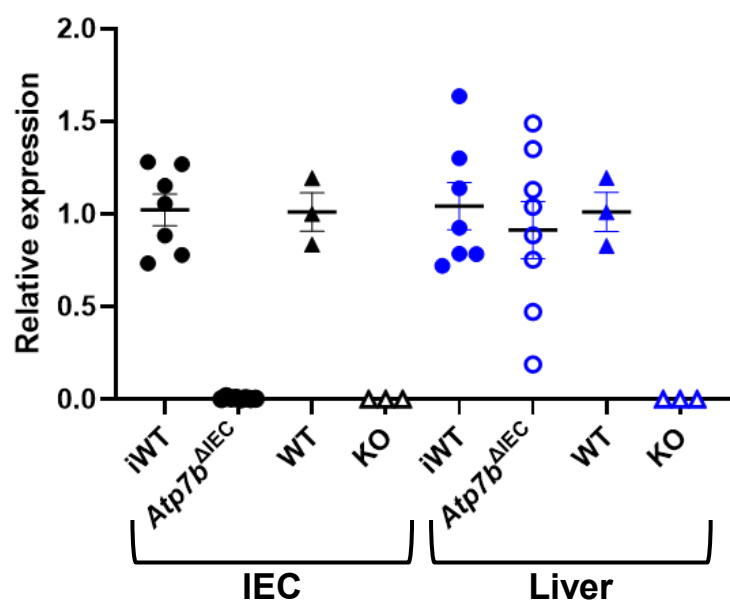**B**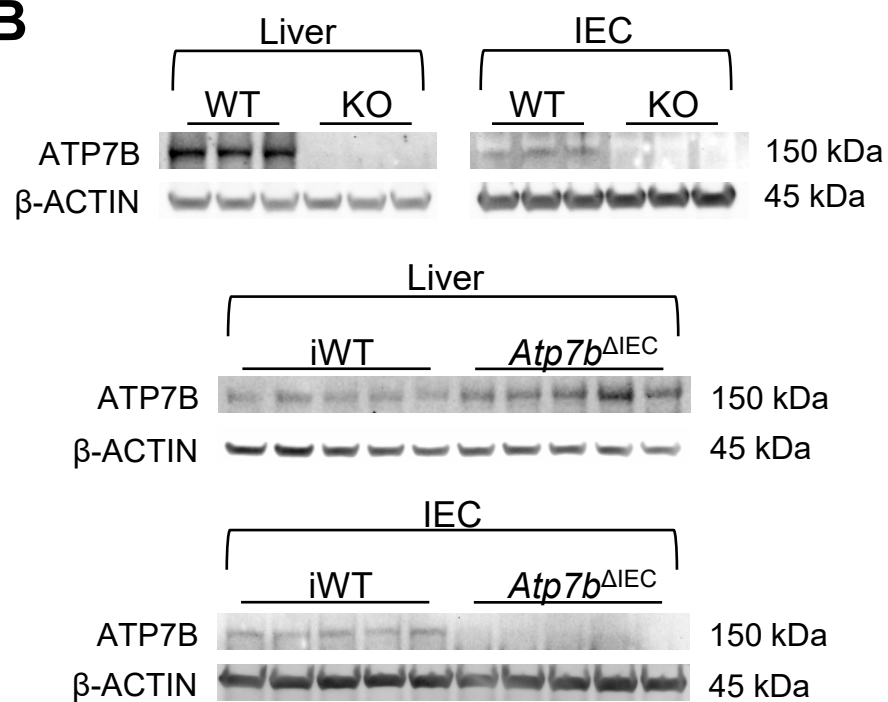**C**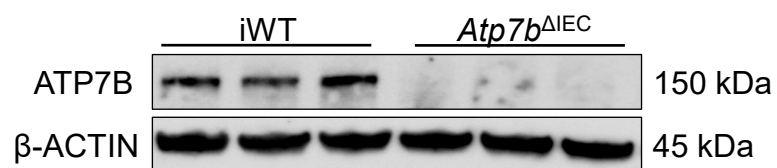

Supplement: Supplemental Figure S1 — Atp7b model validation. A:Atp7b real-time quantitative PCR for 9-week–old Atp7bΔIEC (Lox+/+/Cre+) mice and iWT control (Lox+/+/Cre−), with Atp7b–/– mice and WT control as additional references. Results were normalized to Gapdh in intestinal epithelial cells (IECs) and Ndufs3 in liver. Atp7b expression is reduced 99% in Atp7bΔIEC IECs compared with iWT. This nearly complete reduction of Atp7b expression in the intestine-specific Cre-lox mouse model corresponds to expression levels demonstrated by Muchenditsi et al19 in the hepatocyte-specific Cre-lox mouse model, Atp7bΔHep. Residual expression detected may be due to either contaminating lymphocytes or incomplete inactivation by Cre recombinase in a hemizygous state. Atp7b–/– mice demonstrated no detectable Atp7b transcript levels. Atp7b levels were not different between Atp7bΔIEC and iWT samples in liver, indicating IEC specificity. B and C: Representative ATP7B immunoblot of 9-week–old and 16-week–old Atp7bΔIEC and iWT IEC lysates. Blots were normalized to β-actin. ATP7B protein expression reflects gene expression in specificity and quantity; ATP7B is not detectable in Atp7b–/– mice but, although dramatically reduced, ATP7B can be detected in Atp7bΔIEC mice as a faint band. A and B: Previously published by this group.13 No changes were made to the figure as published in public access reference.13 The figure was published under the Creative Commons Attribution License (CC BY) https://creativecommons.org/licenses/by-nc-nd/4.0/. n = 8 Atp7bΔIEC (A); n = 7 iWT control (A); n = 3 Atp7b–/– mice and WT control (A); n = 5 9-week–old Atp7bΔIEC and iWT IEC lysates (B); n = 3 16-week–old Atp7bΔIEC and iWT IEC lysates (B). Atp7b, ATPase copper transporting β; Atp7b–/–, Atp7b global knockout mice on a C57Bl/6 background; Atp7bΔIEC mice, intestine epithelial cell–specific Atp7b knockout mice; Gapdh, glyceraldehyde 3-phosphate dehydrogenase; iWT, littermate controls (Lox+/+/Cre−) for Atp7bΔIEC mice; Ndufs3, NADH:ubiquinone oxidore [file mmc1.pdf]

**A**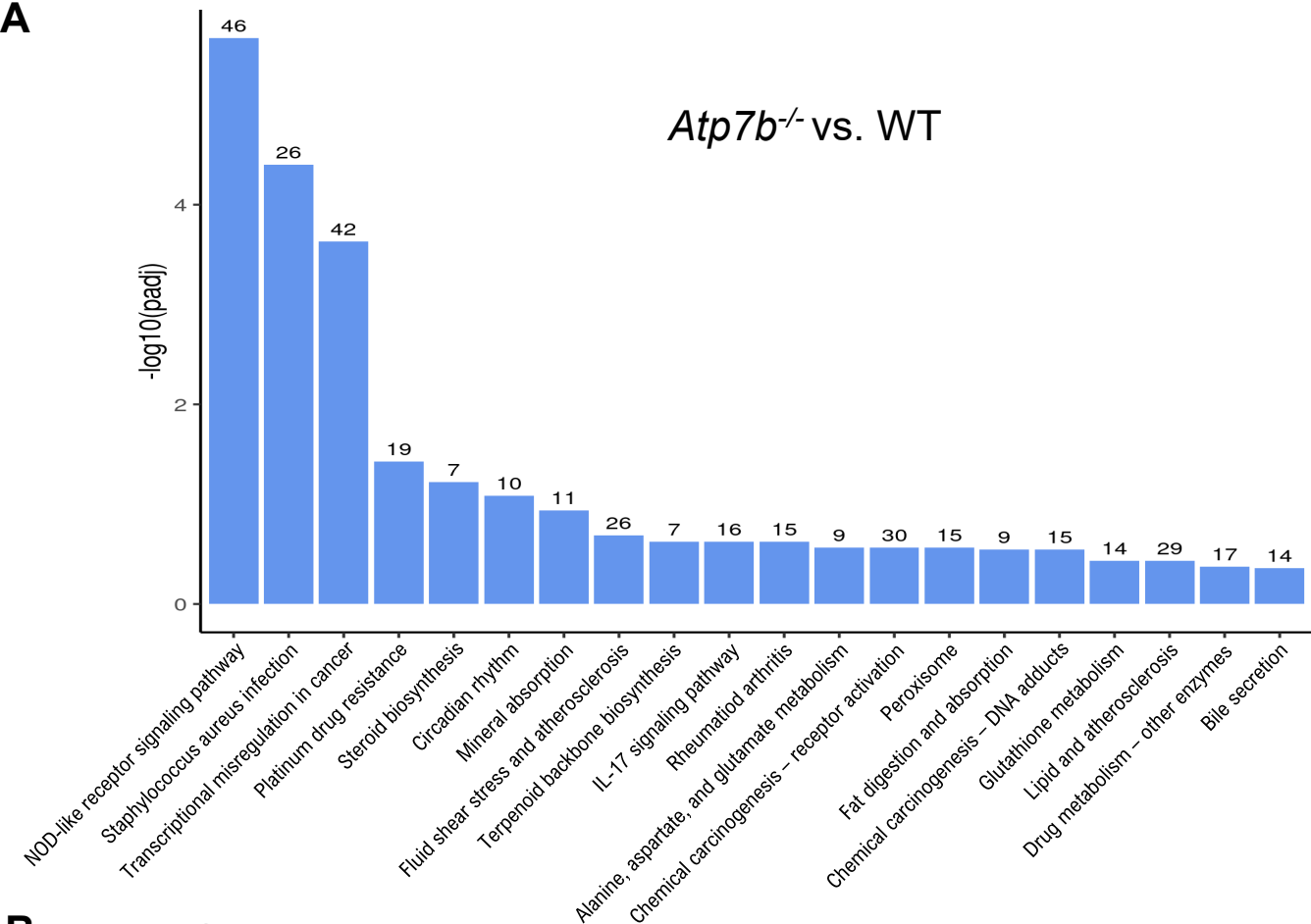**B**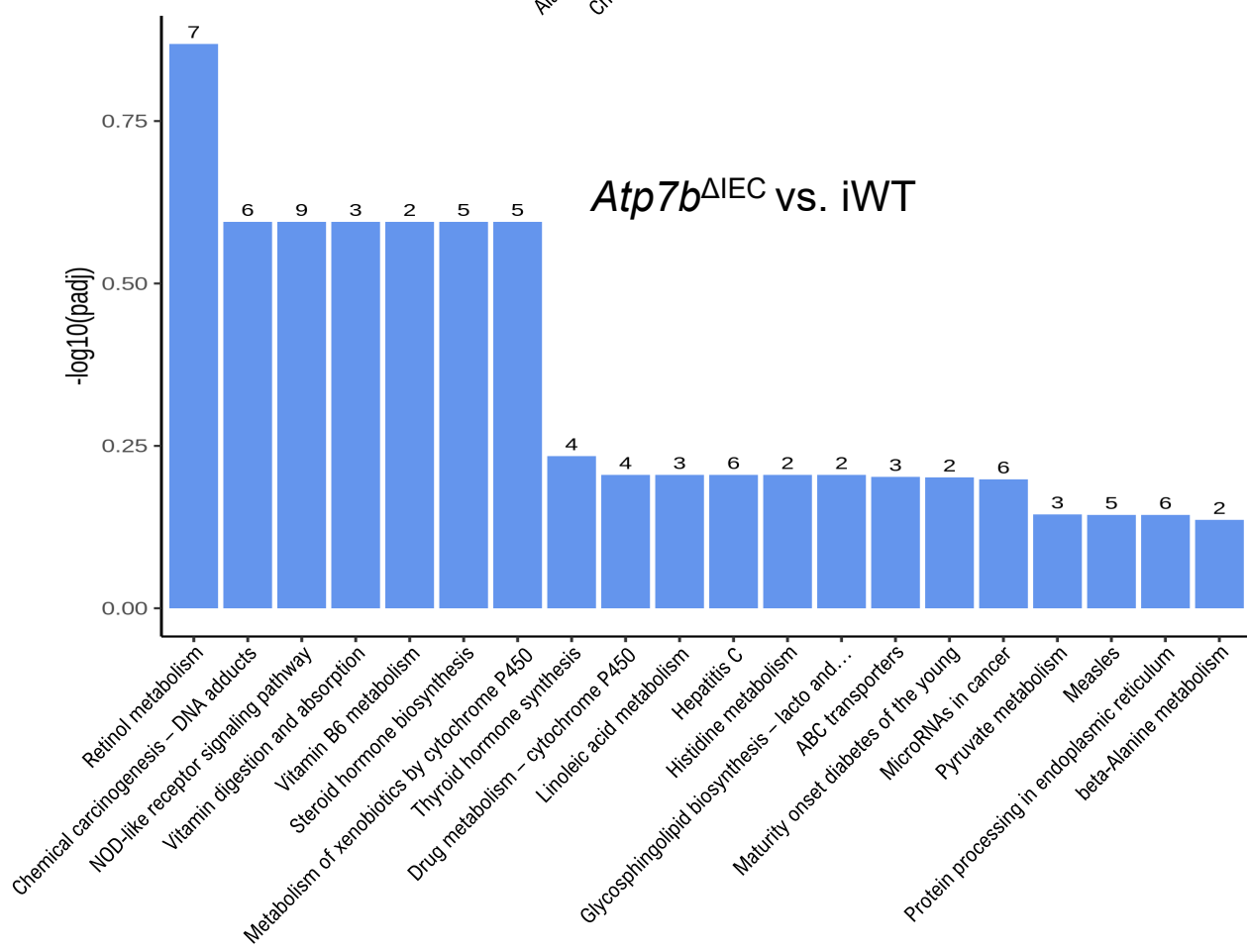

Supplement: Supplemental Figure S2 — RNA-sequencing (RNA-seq) Kyoto Encyclopedia of Genes and Genomes (KEGG) pathway analysis of intestinal epithelial cells (IECs) at 16 weeks of age. Top 20 RNA-seq KEGG pathways of 16-week–old Atp7b–/– versus WT (A) and Atp7bΔIEC versus iWT (B) IECs. Numbers above bar columns are the number of differentially expressed genes in the pathway. n = 6 per genotype (A and B). Atp7b–/–, Atp7b global knockout on C57Bl/6 background; Atp7bΔIEC, intestine epithelial cell–specific knockout on C57Bl/6 background; iWT, wild-type controls (Lox+/+:Cre−) for Atp7bΔIEC; WT, wild-type controls (Atp7b+/+) for Atp7b–/–. [file mmc2.pdf]

**A**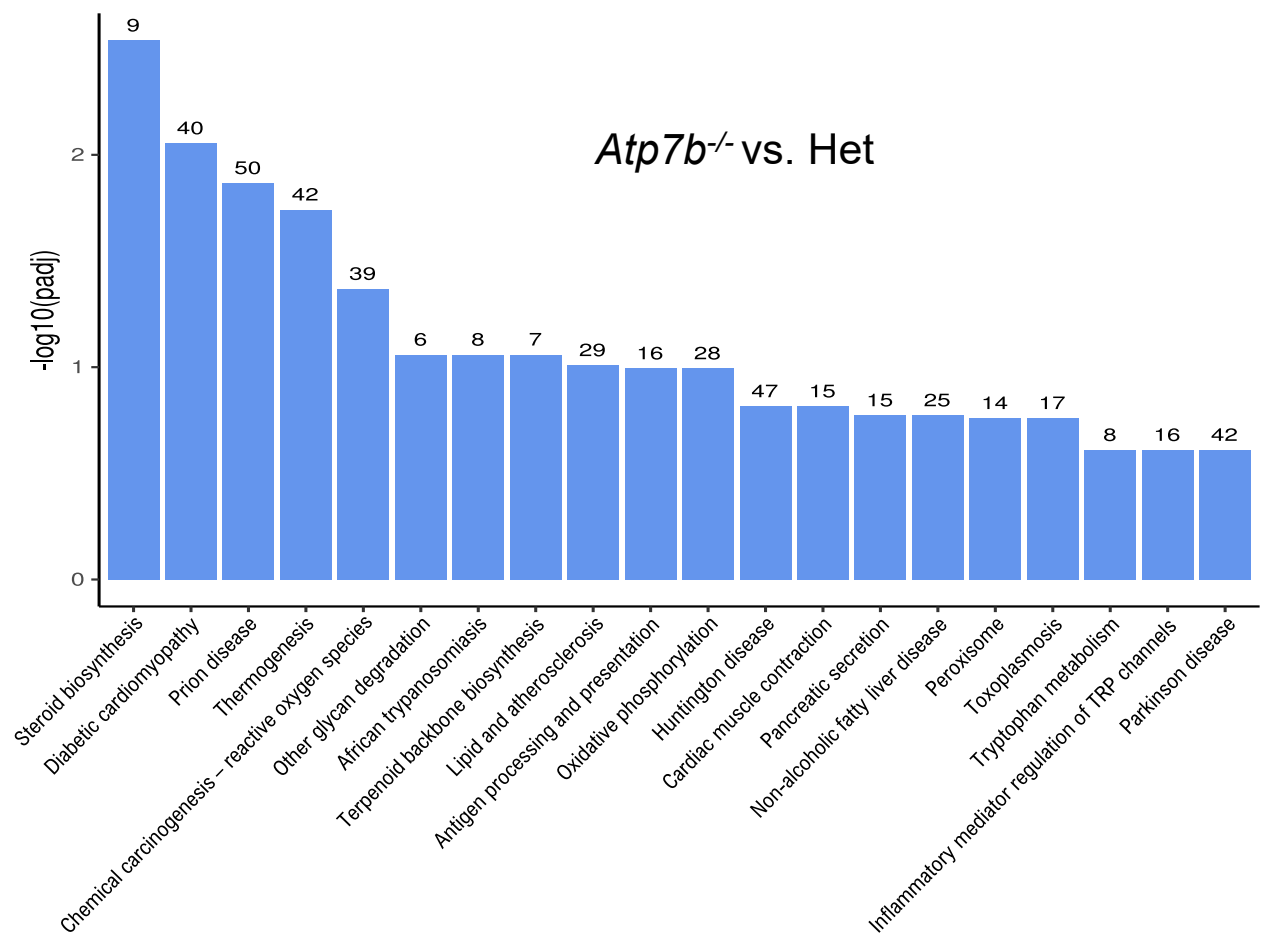**B**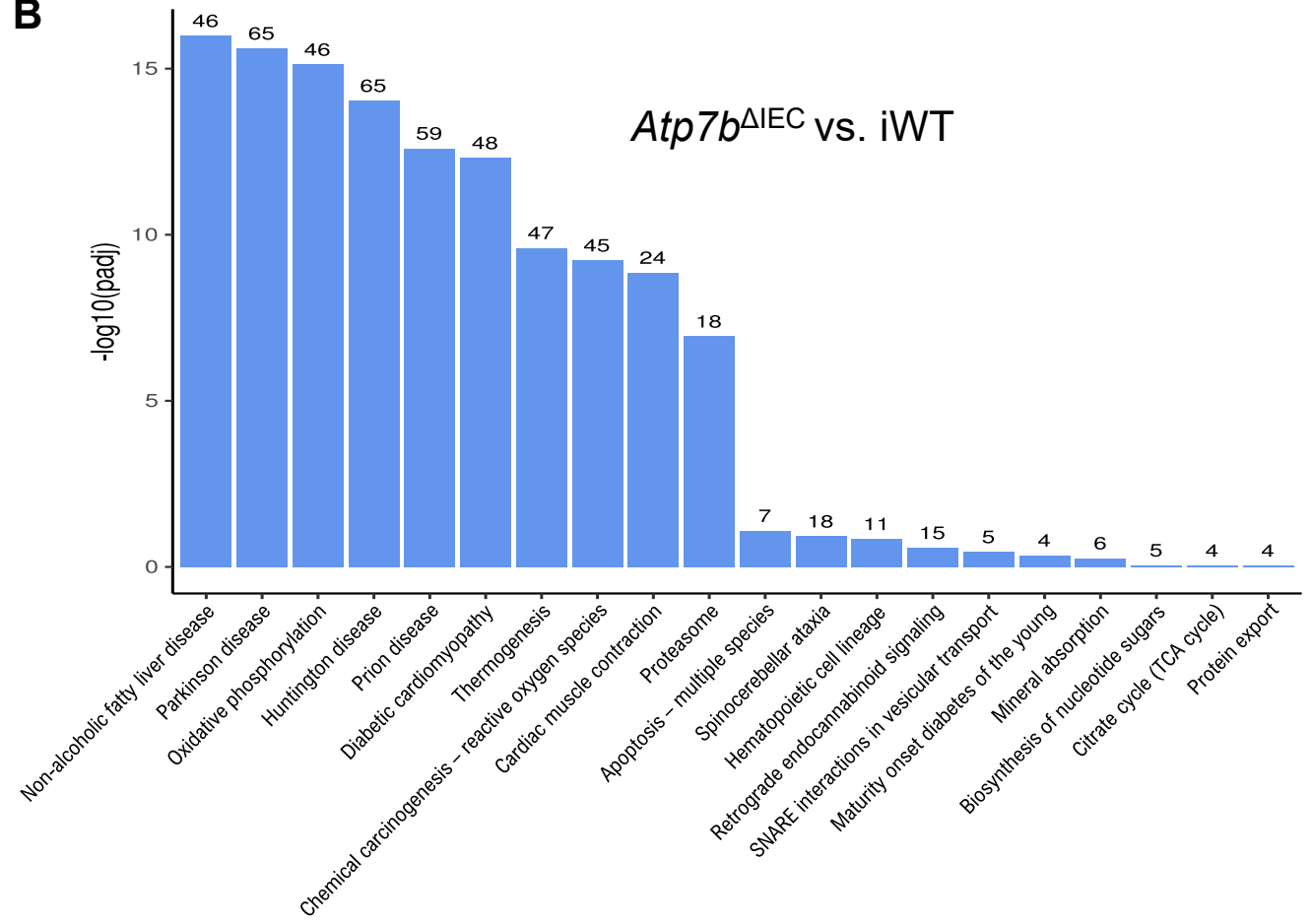

Supplement: Supplemental Figure S3 — RNA-sequencing (RNA-seq) Kyoto Encyclopedia of Genes and Genomes (KEGG) pathway analysis of intestinal epithelial cells (IECs) at 30 weeks of age. Top 20 RNA-seq KEGG pathways of 30-week–old Atp7b–/– versus heterozygous (Het) (A) and Atp7bΔIEC versus iWT (B) IECs. Numbers above bar columns are the number of differentially expressed genes in the pathway. n = 6 per genotype (A and B), except for Het, n = 5 (A). Atp7b–/–, Atp7b global knockout on C57Bl/6 background; Atp7bΔIEC, intestine epithelial cell–specific knockout on C57Bl/6 background; iWT, wild-type controls (Lox+/+:Cre−) for Atp7bΔIEC; TCA, tricarboxylic acid cycle; TRP, transient receptor potential; WT, wild-type controls (Atp7b+/+) for Atp7b–/–. [file mmc3.pdf]

**A**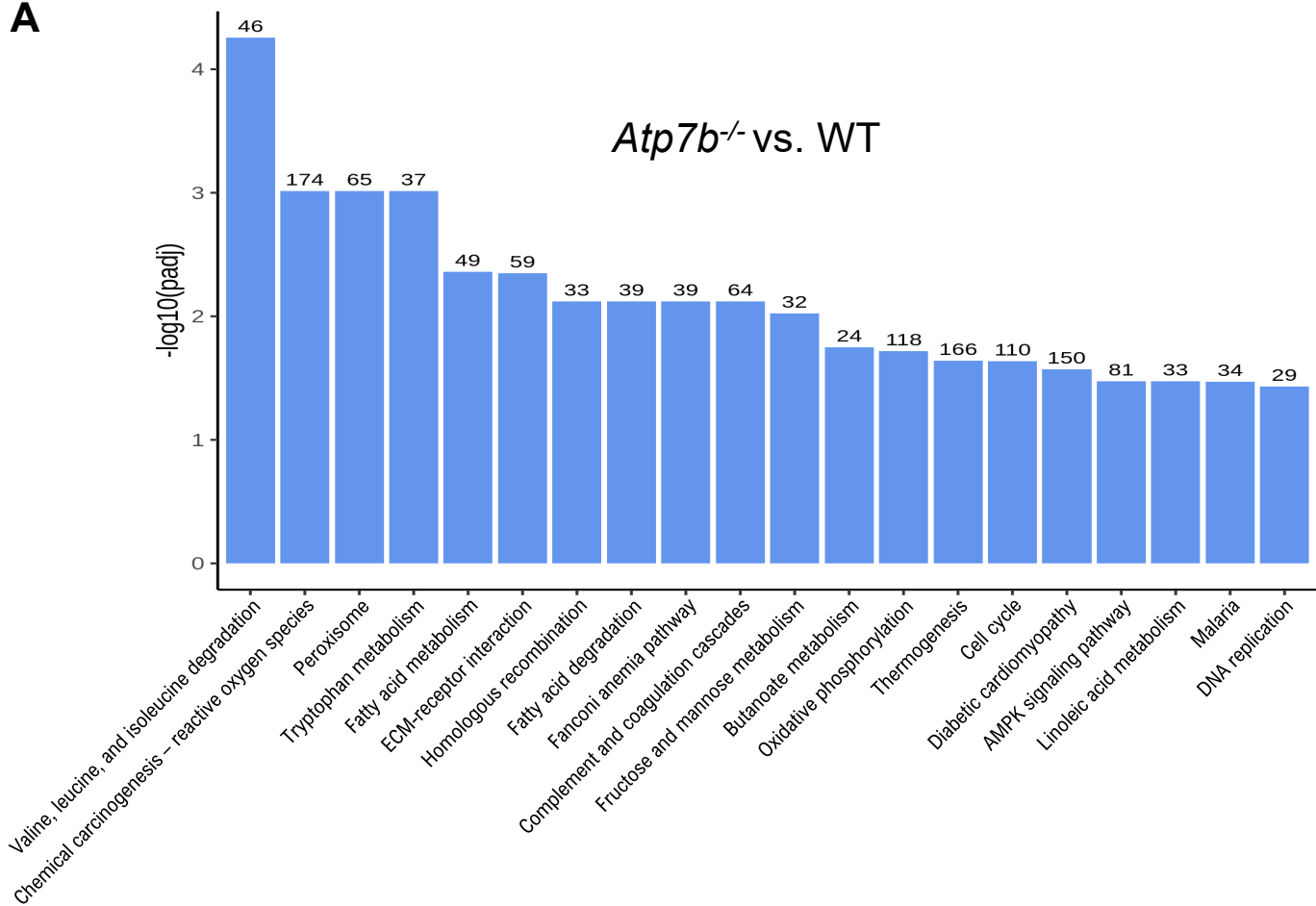**B**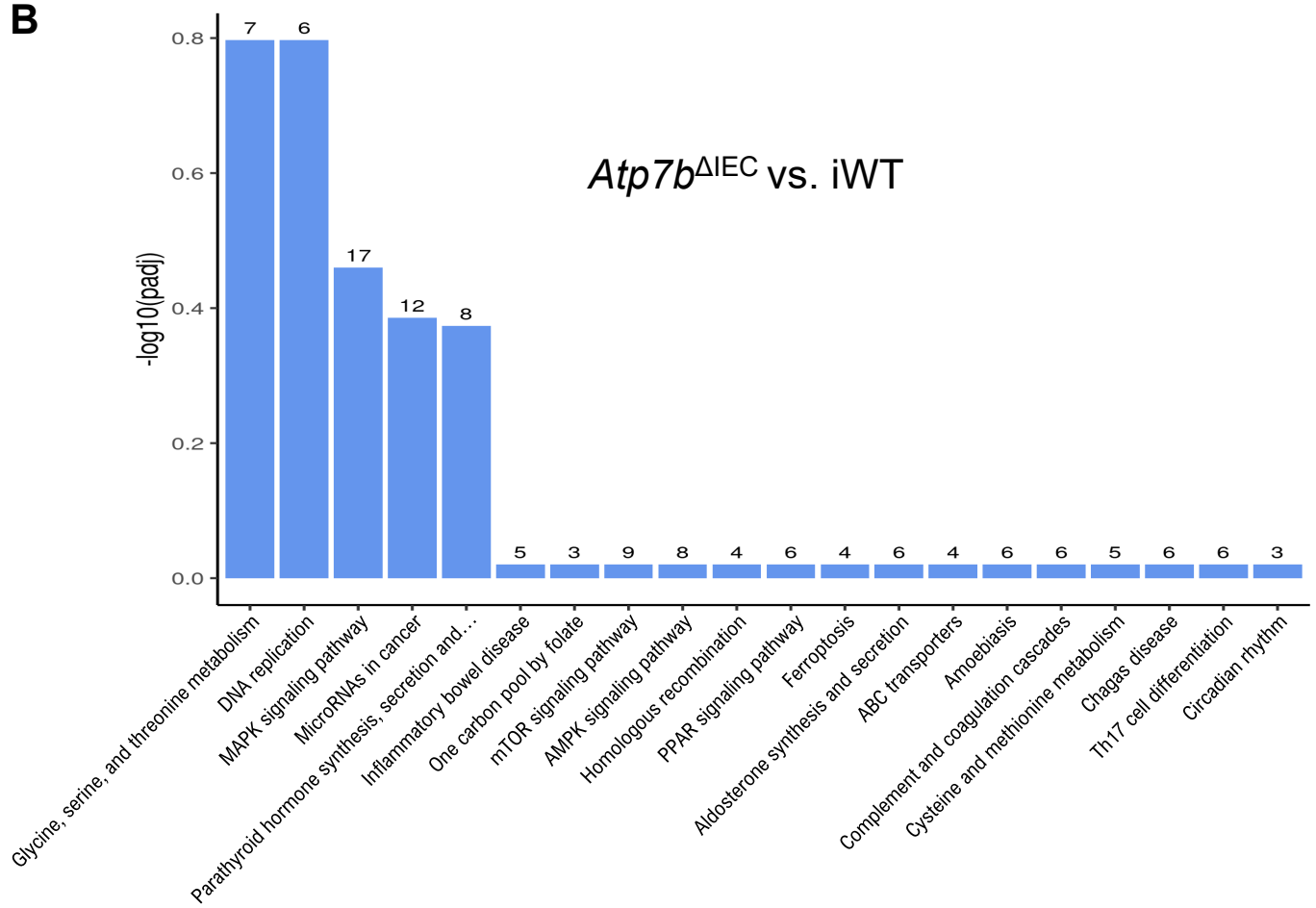

Supplement: Supplemental Figure S4 — RNA-sequencing (RNA-seq) Kyoto Encyclopedia of Genes and Genomes (KEGG) pathway analysis of liver at 16 weeks of age. Top 20 RNA-seq KEGG pathways of 16-week–old Atp7b–/– versus WT (A) and Atp7bΔIEC versus iWT (B) liver. Numbers above bar columns are the number of differentially expressed genes in the pathway. n = 6 per genotype (A and B). AMPK, AMP-activated protein kinase; Atp7b–/–, Atp7b global knockout on C57Bl/6 background; Atp7bΔIEC, intestine epithelial cell–specific knockout on C57Bl/6 background; ECM, extracellular matrix; iWT, wild-type controls (Lox+/+:Cre−) for Atp7bΔIEC; MAPK, mitogen-activated protein kinase; mTOR, mammalian target of rapamycin; PPAR, peroxisome proliferator-activated receptor; Th17, type 17 helper T cell; WT, wild-type controls (Atp7b+/+) for Atp7b–/–. [file mmc4.pdf]

**A**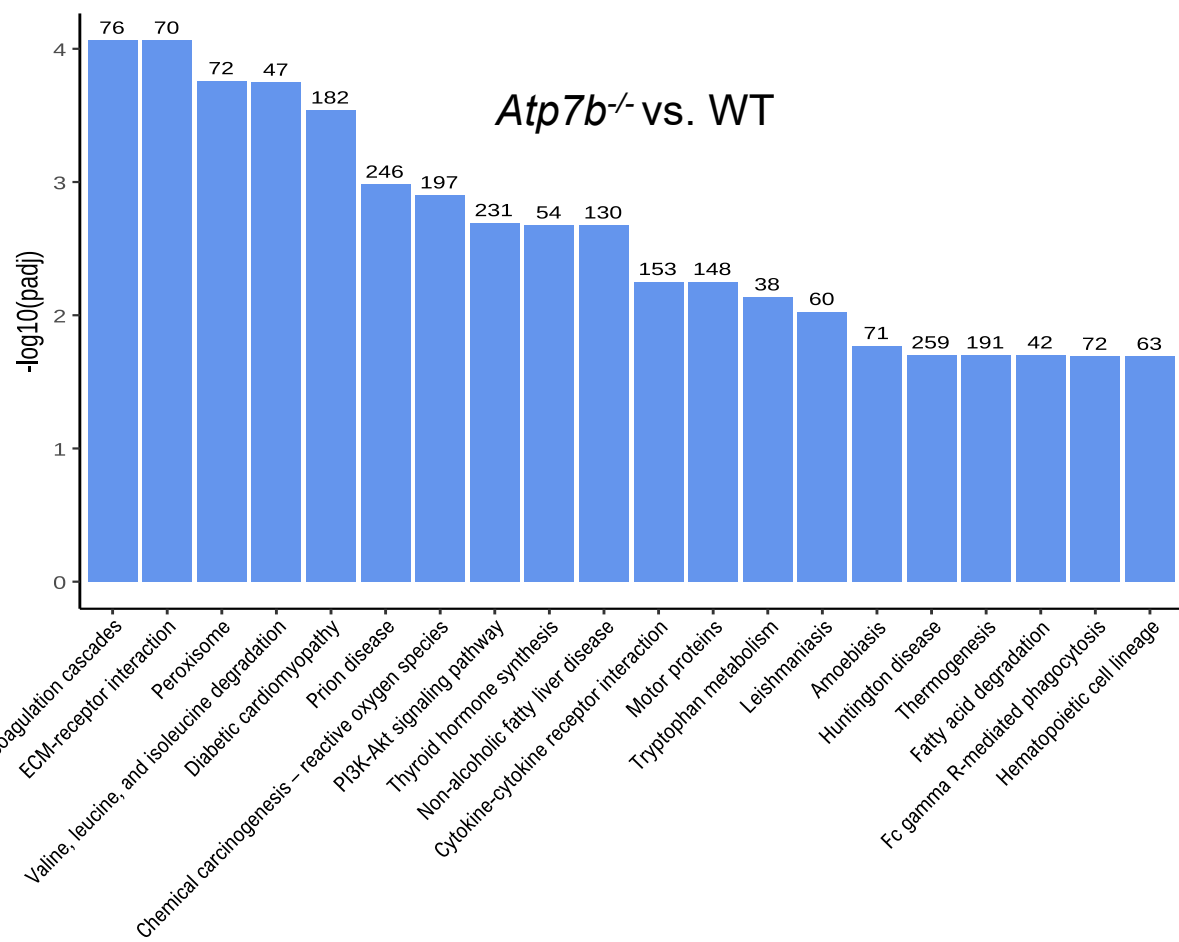**B**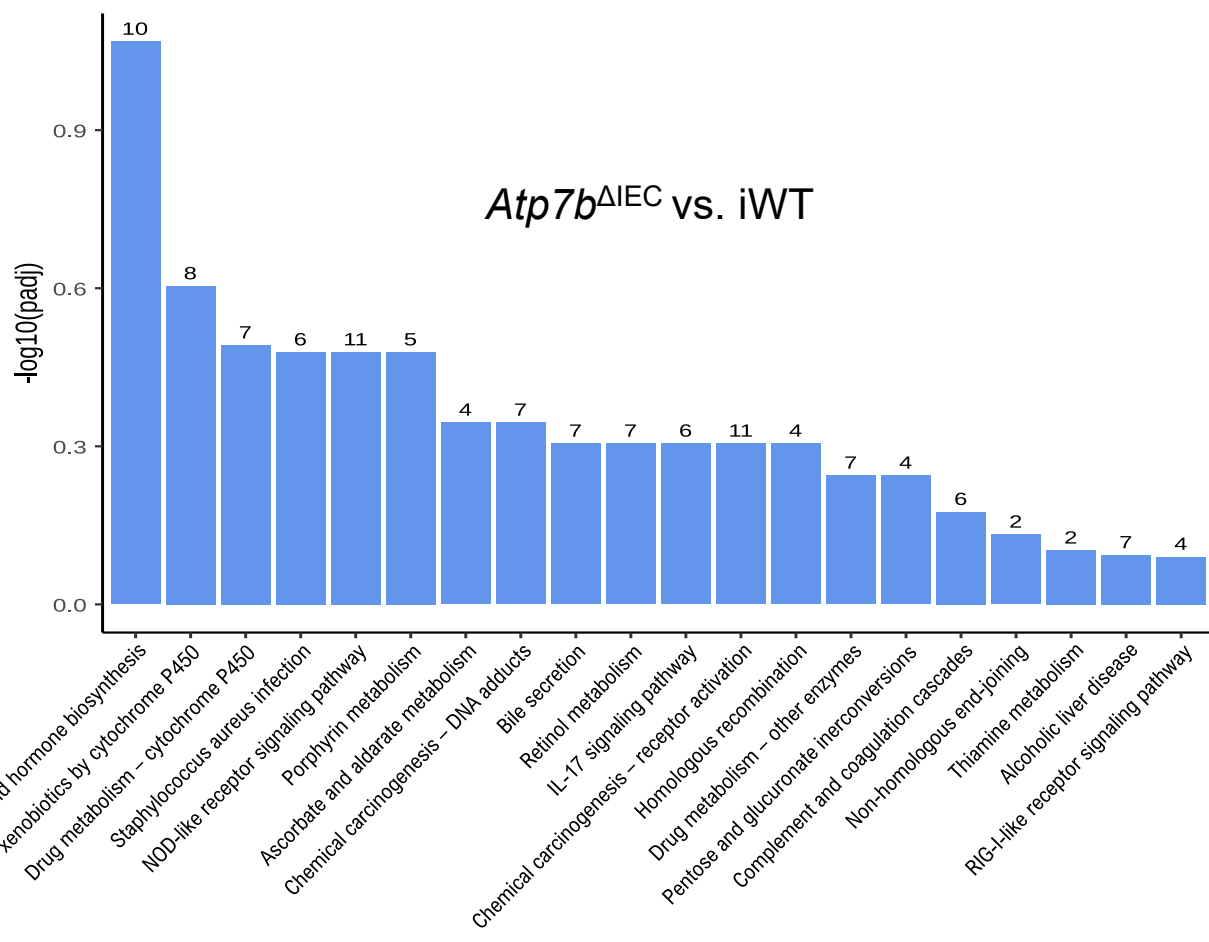

Supplement: Supplemental Figure S5 — RNA-sequencing (RNA-seq) Kyoto Encyclopedia of Genes and Genomes (KEGG) pathway analysis of liver at 30 weeks of age. Top 20 RNA-seq KEGG pathways of 30-week–old Atp7b–/– versus WT (A) and Atp7bΔIEC versus iWT (B) liver. Numbers above bar columns are the number of differentially expressed genes in the pathway. n = 6 per genotype (A and B). Atp7b–/–, Atp7b global knockout on C57Bl/6 background; Atp7bΔIEC, intestine epithelial cell–specific knockout on C57Bl/6 background; ECM, extracellular matrix; iWT, wild-type controls (Lox+/+:Cre−) for Atp7bΔIEC; NOD, nucleotide-binding oligomerization domain; PI3K, phosphatidylinositol 3-kinase; RIG-I, retinoic acid-inducible gene I; WT, wild-type controls (Atp7b+/+) for Atp7b–/–. [file mmc5.pdf]

**A**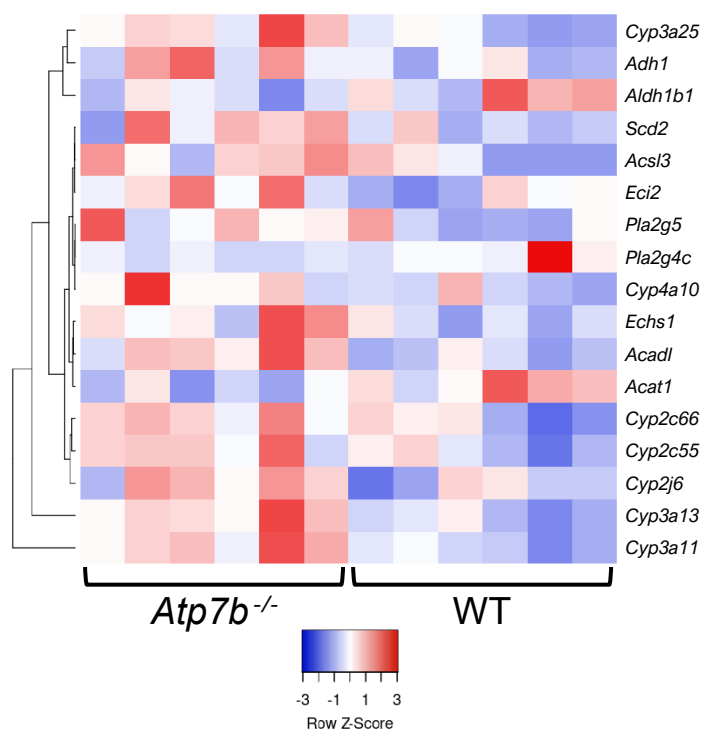**B**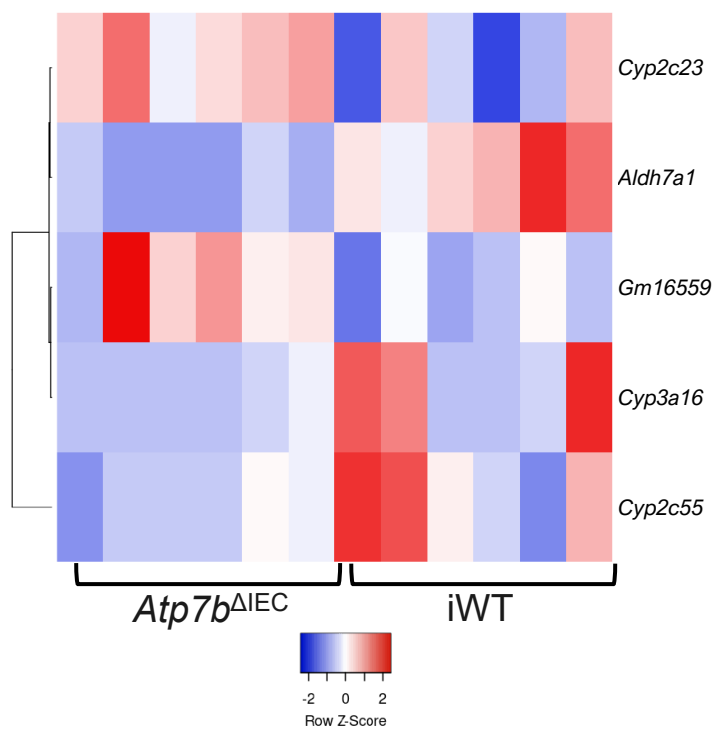**C**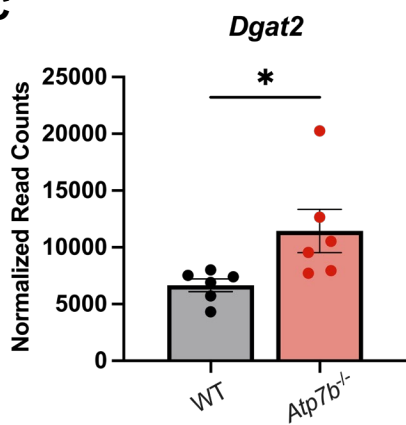**D**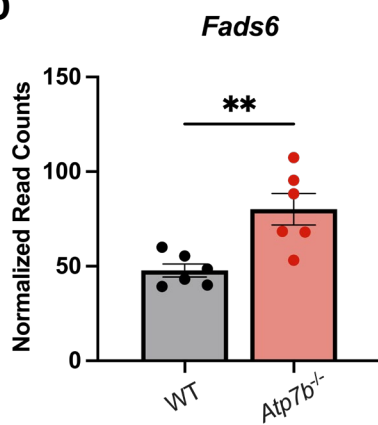**E**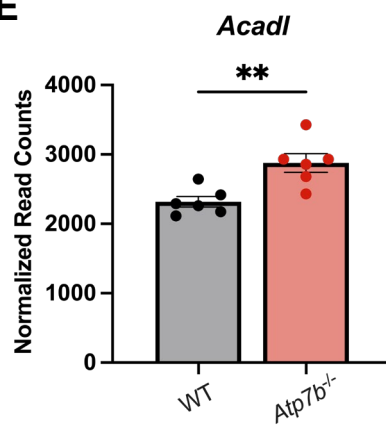**F**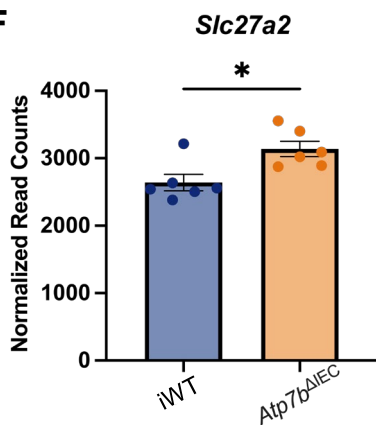**G**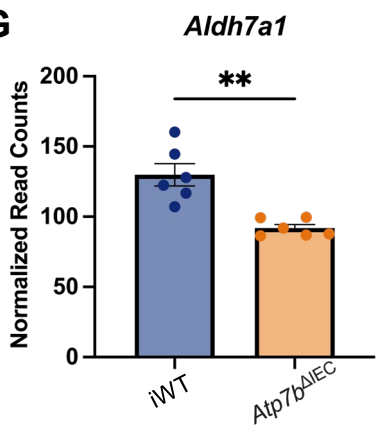

Supplement: Supplemental Figure S6 — Fatty acid metabolism at 16 weeks of age in the intestinal epithelial cells (IECs) of Atp7b–/– and Atp7bΔIEC mice. Hierarchical heat map clustering based on Pearson correlation coefficient for genes in the fatty acid metabolism, fatty acid degradation, and linoleic acid metabolism pathways determined by Kyoto Encyclopedia of Genes and Genomes pathway analysis in the IECs of Atp7b–/– (A) and Atp7bΔIEC (B) mice at 16 weeks of age. Relative gene expression of diacylglycerol O-acyltransferase2 (Dgat2; C), fatty acid desaturase 6 (Fads6; D), and acyl-CoA dehydrogenase long chain (Acadl; E) in Atp7b–/– mice. Relative expression of solute carrier family 27 member 2 (Slc27a2; F) and aldehyde dehydrogenase 7 family member A1 (Aldh7a1; G) in Atp7bΔIEC mice. Statistical significance was determined by t-test. Values are means ± SEM (C–G). (n): Atp7b–/– (6), WT (6), Atp7bΔIEC (6), iWT (6). ∗P < 0.05, ∗∗P < 0.01. Atp7b–/–, Atp7b global knockout on C57Bl/6 background; Atp7bΔIEC, intestine epithelial cell–specific knockout on C57Bl/6 background; iWT, wild-type controls (Lox+/+:Cre−) for Atp7bΔIEC; WT, wild-type controls (Atp7b+/+) for Atp7b–/–. [file mmc6.pdf]

**A**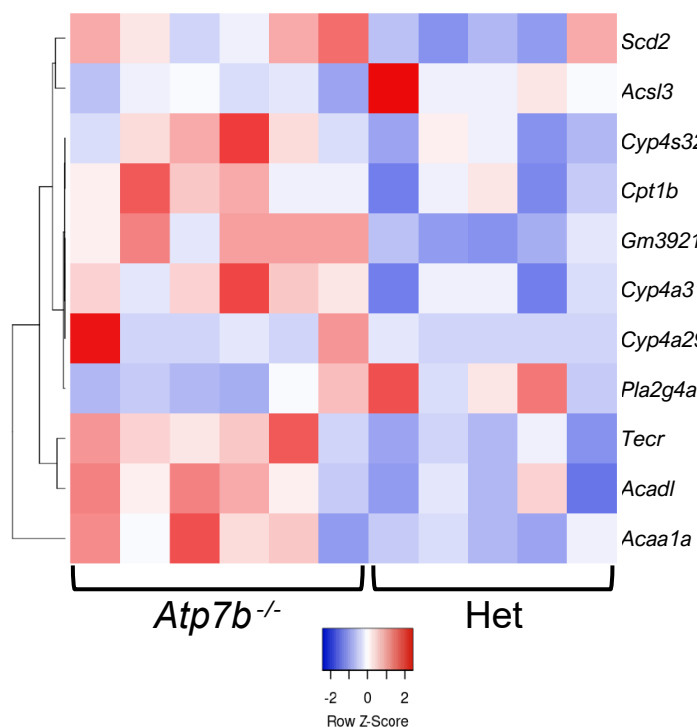**B**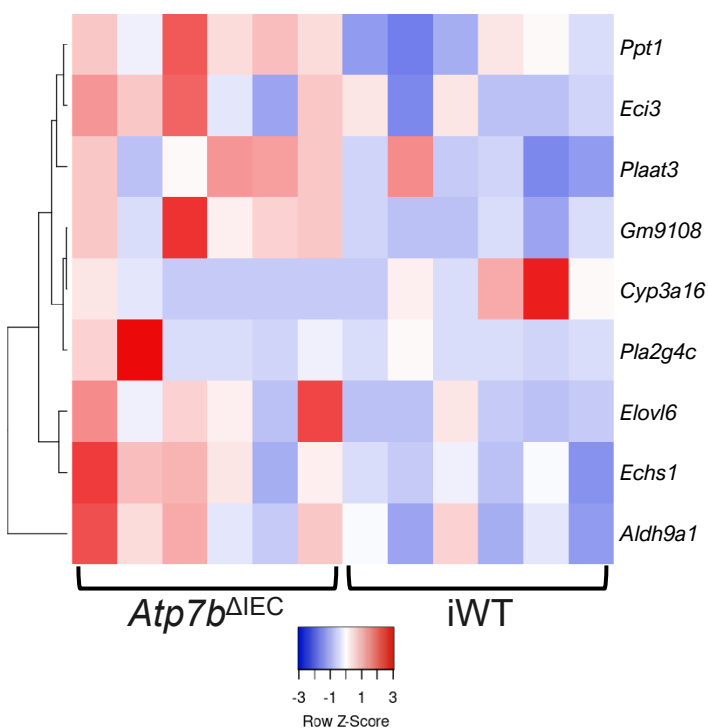**C**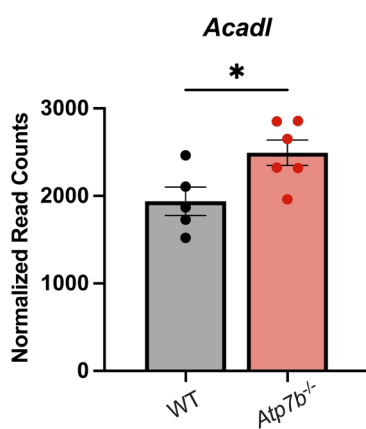**D**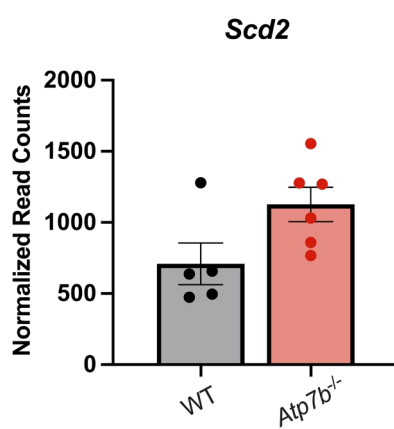**E**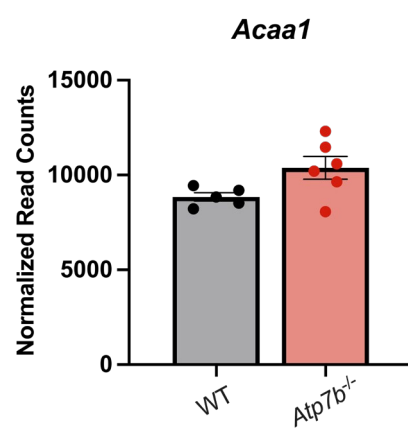**F**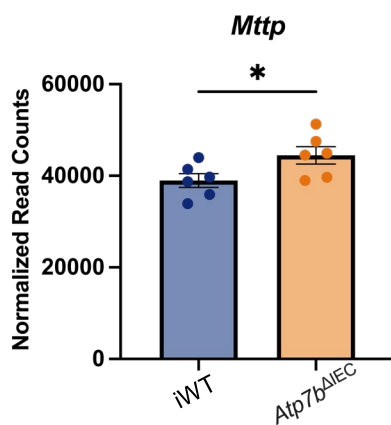**G**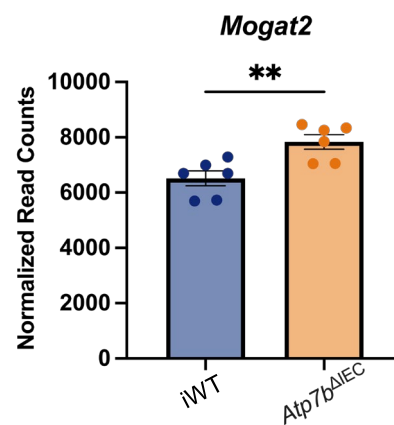

Supplement: Supplemental Figure S7 — Fatty acid metabolism at 30 weeks of age in the intestinal epithelial cells (IECs) of Atp7b–/– and Atp7bΔIEC mice. Hierarchical heat map clustering based on Pearson correlation coefficient for genes in the fatty acid metabolism, fatty acid degradation, and linoleic acid metabolism pathways determined by Kyoto Encyclopedia of Genes and Genomes pathway analysis in the IECs of Atp7b–/– (A) and Atp7bΔIEC (B) mice at 30 weeks of age. Relative gene expression of acyl-CoA dehydrogenase long chain (Acadl; C), stearoyl-CoA desaturase (Scd2; D), and acyl-CoA acyltransferase 1(Acaa1; E) in Atp7b–/–- mice. Relative expression of microsomal triglyceride transfer protein (Mttp; F) and monoacylglycerol O-acyltransferase 2 (Mogat2; G) in Atp7bΔIEC mice. Statistical significance was determined by t-test. Values are means ± SEM (C–G). ∗P < 0.05, ∗∗P < 0.01. (n): Atp7b–/– (6), Heterozygous (Het) (5), Atp7bΔIEC (6), iWT (6). Atp7b–/–, Atp7b global knockout on C57Bl/6 background; Atp7bΔIEC, intestine epithelial cell–specific knockout on C57Bl/6 background; iWT, wild-type controls (Lox+/+:Cre−) for Atp7bΔIEC; WT, wild-type controls (Atp7b+/+) for Atp7b–/–. [file mmc7.pdf]

**A**

16 Weeks

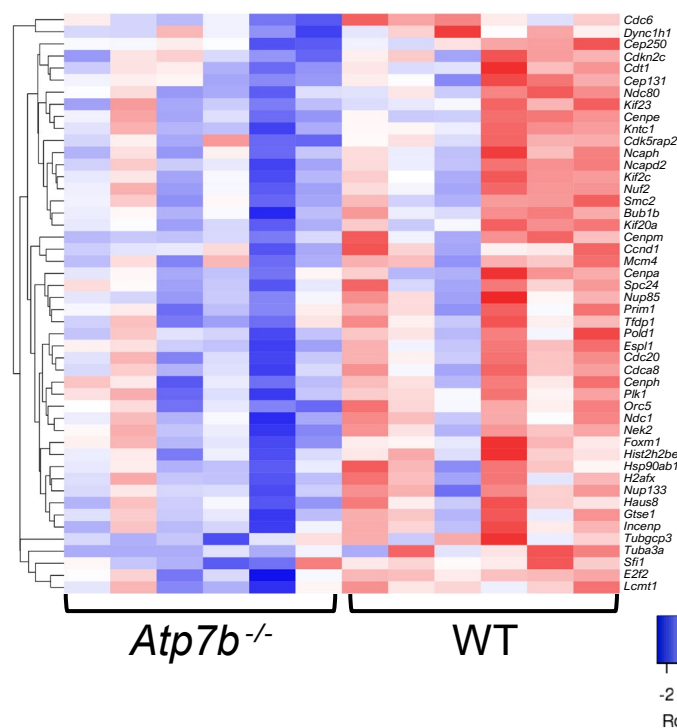**B**

16 Weeks

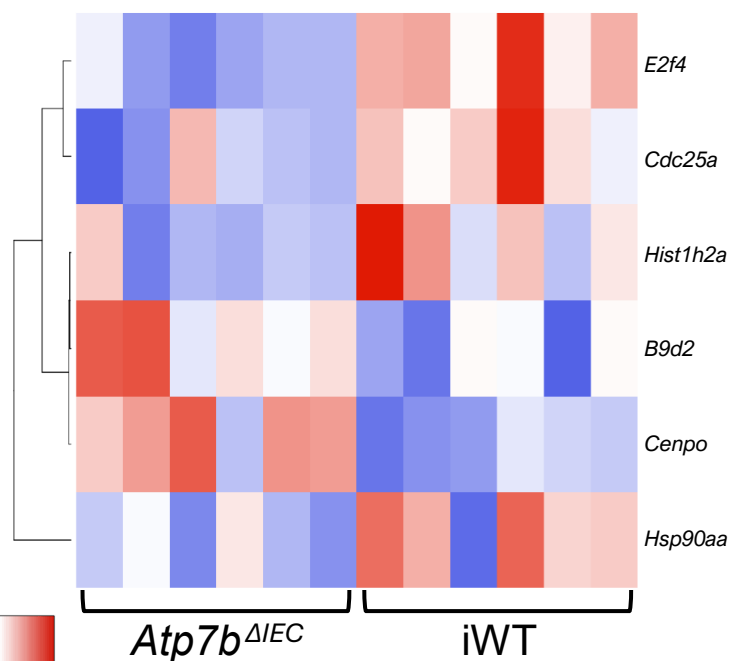**C**

30 Weeks

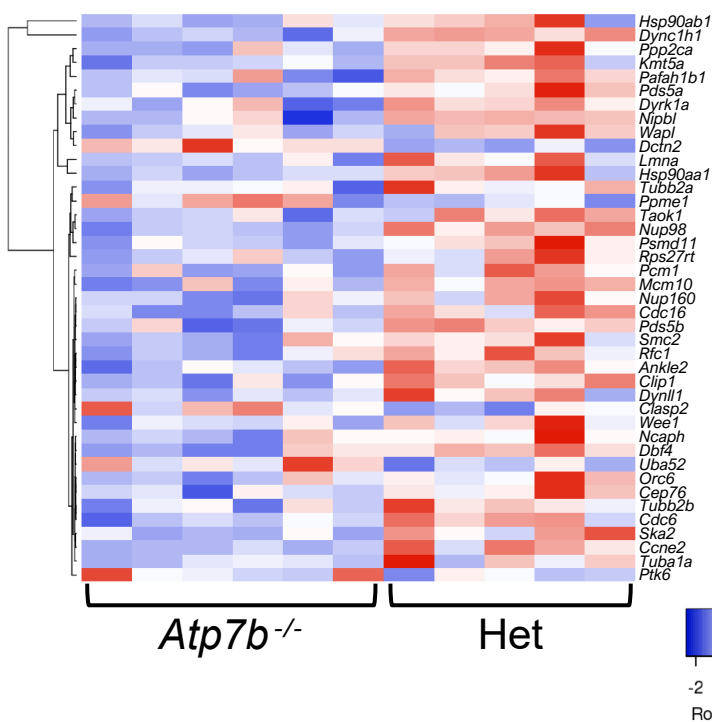**D**

30 Weeks

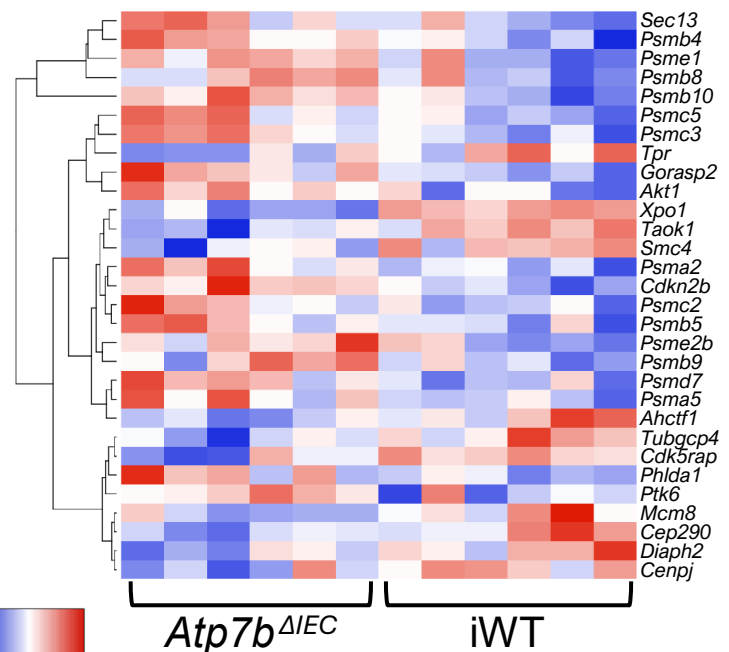

Supplement: Supplemental Figure S8 — Cell cycle, synthesis of DNA, and DNA replication in intestinal epithelial cells (IECs) of Atp7b–/– and Atp7bΔIEC mice. Hierarchical heat map clustering based on Pearson correlation coefficient for genes in the cell cycle, synthesis of DNA, and DNA replication pathways determined by Reactome pathway analysis in the IECs of Atp7b–/– (A) and Atp7bΔIEC (B) mice at 16 weeks of age and in the IECs of Atp7b–/– (C) and Atp7bΔIEC (D) mice at 30 weeks of age. (n): 16 weeks (w): Atp7b–/– (6), WT (6), Atp7bΔIEC (6), iWT (6); 30 w: Atp7b–/– (6), Heterozygous (Het) (5), Atp7bΔIEC (6), iWT (6). Atp7b–/–, Atp7b global knockout on C57Bl/6 background; Atp7bΔIEC, intestine epithelial cell–specific knockout on C57Bl/6 background; iWT, wild-type controls (Lox+/+:Cre−) for Atp7bΔIEC; WT, wild-type controls (Atp7b+/+) for Atp7b–/–. [file mmc8.pdf]
