# Supplementary material for: Inactivation of Atp7b Copper Transporter in Intestinal Epithelial Cells Is Associated with Altered Lipid Processing and Cell Growth Machinery Independent from Hepatic Copper Accumulation and Severity of Liver Histology
Source: Am J Pathol. 2025 Oct 16;196(2):407–27. doi: 10.1016/j.ajpath.2025.09.015 (PMC12881291; doi:10.1016/j.ajpath.2025.09.015)
Supplement: Supplemental Table S1 [file mmc9.docx]

**Supplemental Table S1. Mesenteric white adipose tissue weights normalized to body weight.**

| **16 Weeks** | | | | |
| --- | --- | --- | --- | --- |
| Genotype | n | | MWAT / Body Weight | |
|  | M | F | Male | Female |
| WT | 12 | 13 | 0.0042 ± 0.0012 | 0.0047 ± 0.0025 |
| *Atp7b*^-/-^ | 10 | 11 | 0.0036 ± 0.0008 | 0.0032 ± 0.0010 |
| iWT | 11 | 12 | 0.0050 ± 0.0016 | 0.0038 ± 0.0012^†^ |
| *Atp7b*^ΔIEC^ | 10 | 14 | 0.0041 ± 0.0012 | 0.0037 ± 0.0011 |
| **24 Weeks** | | | | |
| Genotype | n | | MWAT / Body Weight | |
|  | M | F | Male | Female |
| WT | 12 | 10 | 0.0047 ± 0.0013 | 0.0046 ± 0.0013 |
| *Atp7b*^-/-^ | 12 | 9 | 0.0038 ± 0.0012 | 0.0032 ± 0.0009* |
| iWT | 11 | 10 | 0.0076 ± 0.0022 | 0.0056 ± 0.0013^†^ |
| *Atp7b*^ΔIEC^ | 12 | 10 | 0.0081 ± 0.0024 | 0.0054 ± 0.0015^††^ |
| **30 Weeks** | | | | |
| Genotype | n | | MWAT / Body Weight | |
|  | M | F | Male | Female |
| WT | 12 | 13 | 0.0075 ± 0.0019 | 0.0054 ± 0.0024^†^ |
| *Atp7b*^-/-^ | 10 | 14 | 0.0037 ± 0.0008** | 0.0040 ± 0.0012 |
| iWT | 11 | 11 | 0.0081 ± 0.0028 | 0.0087 ± 0.0030 |
| *Atp7b*^ΔIEC^ | 10 | 11 | 0.0080 ± 0.0016 | 0.0085 ± 0.0029 |

Values are mean ± SD and statistical significance was determined by Student’s t test. An asterisk (*) indicates values are significantly different between a WD model (*Atp7b*^-/-^ or *Atp7b*^ΔIEC^) and its respective control (WT or iWT) within the same sex (* p<0.01, ** p<0.001). A dagger (†) indicates values are significantly different between sexes within the same genotype (^†^ p<0.05, ^††^ p<0.001).

*Atp7b*^-/-^, *Atp7b* global knockout on C57Bl/6 background; *Atp7b*^ΔIEC^, intestine epithelial cell-specific knockout on C57Bl/6 background; iWT, wildtype controls (Lox^+/+^:Cre^-^) for *Atp7b*^ΔIEC^; MWAT, mesenteric white adipose tissue; WT, wildtype controls (*Atp7b*^+/+^) for *Atp7b*^-/-^.
