# Supplementary material for: Inactivation of Atp7b Copper Transporter in Intestinal Epithelial Cells Is Associated with Altered Lipid Processing and Cell Growth Machinery Independent from Hepatic Copper Accumulation and Severity of Liver Histology
Source: Am J Pathol. 2025 Oct 16;196(2):407–27. doi: 10.1016/j.ajpath.2025.09.015 (PMC12881291; doi:10.1016/j.ajpath.2025.09.015)
Supplement: Supplemental Table S2 [file mmc10.docx]

**Supplemental Table S2. RNA-seq top 20 KEGG pathways and associated differentially expressed genes in IECs of 16-week *Atp7b*^-/-^ mice (KEGG:** [**https://www.kegg.jp**](https://www.kegg.jp/)**).**

| **KEGG ID** | **Pathway Description** | **Gene Name** |
| --- | --- | --- |
| mmu04621 | NOD-like receptor signaling pathway | *Defa22/Defa21/Defa20/Gm7849/Defa36/Gm7861/Defa2/Defa-ps6/Defa-ps7/*  *Defa26/Defa5/Trp53bp1/Gm14851/Defa23/Nampt/Defa29/Ifnar2/Defa-ps18/*  *Defa32/Gm15502/Defa35/Il1b/Fadd/Oas3/Defa27/Itpr1/Nfkbia/Irf7/Defa31/Nlrp3/Gm15292/Gm7019/Jun/Il18/Hsp90ab1/Nlrp6/Pycard/Defa30/Mapk13/Gm15293/Tbk1/Oas1g/Gsdmcl2/Cxcl2/Txnip/Oas1a* |
| mmu05150 | Staphylococcus aureus infection | *Defa22/Defa21/Defa20/Gm7849/Defa36/Gm7861/Defa2/Defa-ps6/Defa-ps7/*  *Defa26/Defa5/Gm14851/Defa23/Defa29/Defa-ps18/Defa32/Defa35/Defa27/*  *Defa31/A830036E02Rik/Gm15292/Masp1/Defa30/Krt23/Gm15293/Itgb2l* |
| mmu05202 | Transcriptional misregulation in cancer | *Defa22/Defa21/Defa20/Gm7849/Defa36/Gm7861/Defa2/Defa-ps6/Defa-ps7/*  *Defa26/Defa5/Gm14851/Defa23/Defa29/Defa-ps18/Per2/Defa32/Mycn/Id2/*  *H3f3c/Defa35/Nr4a3/Defa27/Ncor1/Gadd45g/Defa31/Il2rb/Gm15292/Gm7019/Six4/Pparg/Cebpa/Spi1/Plat/Cdkn2c/Defa30/Gm15293/Il1r2/Bmi1/Flt3/Taf15/Hhex* |
| mmu01524 | Platinum drug resistance | *Map3k5/Gsta1/Gm3776/Gm10639/Gsta4/Abcc2/Atp7b/Gstm2/Fadd/Gstm2-ps1/Erbb2/Gm10053/Gm7019/Top2a/Pmaip1/Bbc3/Gsta2/Atp7a/Msh6* |
| mmu00100 | Steroid biosynthesis | *Msmo1/Cyp51/Hsd17b7/Lss/Dhcr24/Nsdhl/Sqle* |
| mmu04710 | Circadian rhythm | *Bhlhe40/Per3/Cry2/Per2/Nr1d2/Dbp/Rorc/Per1/Arntl/Nfil3* |
| mmu04978 | Mineral absorption | *Mt1/Atp2b1/Slc6a19/Atp7b/Slc11a2/Slc8a2/Atp7a/Atp1b1/Slc34a3/Slc5a1/Trpv6* |
| mmu05418 | Fluid shear stress and atherosclerosis | *Map3k5/Gsta1/Vegfa/Gm3776/Gm10639/Gsta4/Fos/Dusp1/Gstm2/Il1b/Gstm2-ps1/Gm14269/Jun/Ctsl/Gsta2/Hsp90ab1/Plat/Sdc4/Sdc2/Mapk13/Thbd/*  *Rac3/Il1r2/Gm10241/Calml4/Nqo1* |
| mmu00900 | Terpenoid backbone biosynthesis | *Mvk/Idi1/Nus1/Acat1/Gm3571/Hmgcr/Mvd* |
| mmu04657 | IL-17 signaling pathway | *Fosb/Ccl20/Fos/Ptgs2/Il1b/Fadd/Mapk4/Nfkbia/Mapk6/Jun/Ccl17/Hsp90ab1/Mapk13/Il17rc/Tbk1/Cxcl2* |
| mmu05323 | Rheumatoid arthritis | *Vegfa/Ccl20/Ccl3/Tnfrsf11a/Fos/Atp6v1d/Il1b/Il15/Jun/Ctsl/Il18/Atp6v0e2/Itgb2l/Cxcl2/Atp6v1e1* |
| mmu00250 | Alanine, aspartate and glutamate metabolism | *Aspa/Got1/Gpt/Aldh4a1/Asl/Ddo/Il4i1/Rimkla/Agxt2* |
| mmu05207 | Chemical carcinogenesis - receptor activation | *Nr1i3/Gsta1/Cyp3a13/Vegfa/Gm3776/Cyp3a11/Gm10639/Cyp3a25/Gsta4/Fos/Arrb1/Ugt1a9/Creb3l3/Gstm2/Gstm2-ps1/Ar/Ugt2b34/Fgf9/Cacna1c/*  *Gnai3/Jun/Cdc6/Ccnd1/Gsta2/Hsp90ab1/Adrb2/Dll4/Paqr8/Fgf2/Klf4* |
| mmu04146 | Peroxisome | *Mvk/Hao2/Idh1/Acox2/Pxmp2/Pex6/Paox/Pex11a/Ddo/Acsl3/Nudt12/Eci2/Pex19/Pex2/Gm8566* |
| mmu04975 | Fat digestion and absorption | *Dgat2/Gm47528/Apoa1/Abca1/Acat1/Cd36/Fabp2/Pnliprp1/Pla2g5* |
| mmu05204 | Chemical carcinogenesis - DNA adducts | *Gsta1/Cyp3a13/Gm3776/Cyp3a11/Gm10639/Cyp3a25/Gsta4/Ptgs2/Ugt1a9/Gstm2/Gstm2-ps1/Ugt2b34/Cyp2c55/Cyp2c66/Gsta2* |
| mmu00480 | Glutathione metabolism | *Gsta1/Gm3776/Gm10639/Gsta4/Gclc/Nat8/Idh1/Nat8f5/Gstm2/Gstm2-ps1/*  *Nat8f2/Gsta2/Nat8f6/Rrm2* |
| mmu05417 | Lipid and atherosclerosis | *Map3k5/Ccl3/Tnfsf10/Fos/Gm47528/Ern1/Apoa1/Pou2f3/Casp6/Il1b/Abca1/Itpr1/Nfkbia/Irf7/Gm10053/Nlrp3/Cd36/Jun/Il18/Cyp2j6/Pparg/Hsp90ab1/Pycard/Mapk13/Tbk1/Rock2/Cxcl2/Calml4/Camk2a* |
| mmu00983 | Drug metabolism - other enzymes | *Ces2a/Gsta1/Gm3776/Gm10639/Ces1d/Gsta4/Ugt1a9/Gstm2/Gstm2-ps1*  */Gm7208/Ugt2b34/Ces2c/Gsta2/Ces2d-ps/Hprt/Tk1/Rrm2* |
| mmu04976 | Bile secretion | *Gm42686/Abcb1a/Ugt1a9/Abcc2/Nr0b2/Slc2a1/Ugt2b34/Slc10a2/Hmgcr/Slc10a1/Slc51a/Atp1b1/Gm15592/Slc5a1* |
