# Supplementary material for: Inactivation of Atp7b Copper Transporter in Intestinal Epithelial Cells Is Associated with Altered Lipid Processing and Cell Growth Machinery Independent from Hepatic Copper Accumulation and Severity of Liver Histology
Source: Am J Pathol. 2025 Oct 16;196(2):407–27. doi: 10.1016/j.ajpath.2025.09.015 (PMC12881291; doi:10.1016/j.ajpath.2025.09.015)
Supplement: Supplemental Table S3 [file mmc11.docx]

**Supplemental Table S3. RNA-seq top 20 KEGG pathways and associated differentially expressed genes in IECs of 16-week *Atp7b*^ΔIEC^ mice (KEGG:** [**https://www.kegg.jp**](https://www.kegg.jp/)**).**

| **KEGG ID** | **Pathway Description** | **Gene Name** |
| --- | --- | --- |
| mmu00830 | Retinol metabolism | *Cyp3a16/Gm16559/Cyp2c55/Lrat/Gm15368/Cyp2c23/Cyp2a5* |
| mmu05204 | Chemical carcinogenesis - DNA adducts | *Cyp3a16/Cyp2c55/Gm15368/Cyp2c23/Gstm1/Cyp1b1* |
| mmu04621 | NOD-like receptor signaling pathway | *Gm7849/Txnip/Hsp90aa1/Gbp5/Ifnar1/Oas1b/Oas2/Oas1a/Traf5* |
| mmu04977 | Vitamin digestion and absorption | *Gm45674/Lrat/Gm13270* |
| mmu00750 | Vitamin B6 metabolism | *D030028A08Rik/Psat1* |
| mmu00140 | Steroid hormone biosynthesis | *Cyp3a16/Cyp2c55/Gm15368/Cyp2c23/Cyp1b1* |
| mmu00980 | Metabolism of xenobiotics by cytochrome P450 | *Gm16559/Gm15368/Gstm1/Aldh3b3/Cyp1b1* |
| mmu04918 | Thyroid hormone synthesis | *Pdia4/Gsr/Pax8/Duoxa2* |
| mmu00982 | Drug metabolism - cytochrome P450 | *Gm16559/Gm15368/Gstm1/Aldh3b3* |
| mmu00591 | Linoleic acid metabolism | *Cyp3a16/Cyp2c55/Cyp2c23* |
| mmu05160 | Hepatitis C | *Cldn2/Gm21178/Ifnar1/Oas1b/Oas2/Oas1a* |
| mmu00340 | Histidine metabolism | *Aldh7a1/Aldh3b3* |
| mmu00601 | Glycosphingolipid biosynthesis - lacto and neolacto series | *B3galt2/Fut2* |
| mmu02010 | ABC transporters | *Gm45674/Abcg3/Abcc6* |
| mmu04950 | Maturity onset diabetes of the young | *Gm17743/Bhlha15* |
| mmu05206 | MicroRNAs in cancer | *Timp3/Gm45674/Cdc25a/Marcks/Pdcd4/Cyp1b1* |
| mmu00620 | Pyruvate metabolism | *Aldh7a1/Gm16559/4833422C13Rik* |
| mmu05162 | Measles | *Cd209d/Ifnar1/Oas1b/Oas2/Oas1a* |
| mmu04141 | Protein processing in endoplasmic reticulum | *Hyou1/Hsph1/Hsp90aa1/Pdia4/Gm15542/Hspa4l* |
| mmu00410 | beta-Alanine metabolism | *Aldh7a1/Aldh3b3* |
