# Supplementary material for: Inactivation of Atp7b Copper Transporter in Intestinal Epithelial Cells Is Associated with Altered Lipid Processing and Cell Growth Machinery Independent from Hepatic Copper Accumulation and Severity of Liver Histology
Source: Am J Pathol. 2025 Oct 16;196(2):407–27. doi: 10.1016/j.ajpath.2025.09.015 (PMC12881291; doi:10.1016/j.ajpath.2025.09.015)
Supplement: Supplemental Table S4 [file mmc12.docx]

**Supplemental Table S4. RNA-seq top 20 KEGG pathways and associated differentially expressed genes in IECs of 30-week *Atp7b*^-/-^ mice (KEGG:** [**https://www.kegg.jp**](https://www.kegg.jp/)**).**

| **KEGG ID** | **Pathway Description** | **Gene Name** |
| --- | --- | --- |
| mmu00100 | Steroid biosynthesis | *Fdft1/Tm7sf2/Nsdhl/Sqle/Hsd17b7/Cyp51/Cyp27b1/Soat1/Msmo1* |
| mmu05415 | Diabetic cardiomyopathy | *Gm13340/Ren1/Gm28661/Gm29216/Pik3r3/mt-Co3/Ager/Mapk13/mt-Atp8/mt-Nd2/Uqcrb/Camk2g/Gm10222/mt-Cytb/Mcpt4/mt-Nd3/Ndufv2/*  *mt-Nd4/Cybb/Ndufb10/Cox6c/mt-Nd1/Uqcrfs1/Prkcg/mt-Atp6/Cpt1b/*  *Ndufa5/Ndufa3/Ppp1ccb/Mpc2/mt-Co1/Gm28439/Atp5pb/Uqcr10/*  *mt-Co2/Mapk11/Atp2a3/Gm10925/Cox6b1/Vdac3-ps1* |
| mmu05020 | Prion disease | *Gm13340/Gm28661/C1qa/Gm29216/Stip1/Pik3r3/Eif2ak3/Hspa1b/mt-Co3/Mapk13/mt-Atp8/mt-Nd2/Uqcrb/Hspa8/Gm10222/mt-Cytb/mt-Nd3/*  *Ndufv2/mt-Nd4/Cybb/Hspa1a/Ndufb10/Gm3756/Cox6c/Gm15459/*  *mt-Nd1/Uqcrfs1/Tuba1a/C1qc/mt-Atp6/C1qb/Ndufa5/Itpr1/Ndufa3/*  *Adrm1/Tubb2b/Tubb2a/mt-Co1/Gm28439/Atp5pb/Uqcr10/mt-Co2/*  *Mapk11/Gm10925/Cox6b1/Gm7232/Psmd11/Klc2/Vdac3-ps1/Psma1* |
| mmu04714 | Thermogenesis | *Bmp8b/Gm13340/Gm28661/Gm29216/Bmp8a/mt-Co3/Mapk13/mt-Atp8/*  *mt-Nd2/Uqcrb/Gm10222/mt-Cytb/Atp5k/mt-Nd3/Ndufv2/mt-Nd4/Acsl3/*  *Ndufb10/Cox6c/mt-Nd1/Uqcrfs1/mt-Atp6/Cpt1b/Ndufa5/Pparg/Coa5/*  *Ndufa3/Adcy8/mt-Co1/Gm28439/Atp5pb/Gm10221/Frs2/Uqcr10/mt-Co2/Rps6ka2/Mapk11/Gm10925/Cox6b1/Prkag3/Fgfr1/Tsc1* |
| mmu05208 | Chemical carcinogenesis - reactive oxygen species | *Gm13340/Egfr/Gm28661/Gm29216/Pik3r3/mt-Co3/Mapk13/mt-Atp8/mt-Nd2/Uqcrb/Gm10222/mt-Cytb/mt-Nd3/Cyp1a1/Vegfa/Ndufv2/mt-Nd4/*  *Ndufb10/Cox6c/Nox1/mt-Nd1/Uqcrfs1/Gstm2/mt-Atp6/Chuk/Ndufa5/*  *Ndufa3/As3mt/Gsta4/mt-Co1/Gm28439/Atp5pb/Slc26a1/Uqcr10/mt-Co2/Mapk11/Gm10925/Cox6b1/Vdac3-ps1* |
| mmu00511 | Other glycan degradation | *Fuca1/Man2c1/Man2b2/Neu1/Hexdc/Man2b1* |
| mmu05143 | African trypanosomiasis | *Hbb-bs/Hbb-bt/Hba-a1/Hba-a2/Prkcg/Il18/Tlr9/Ido1* |
| mmu00900 | Terpenoid backbone biosynthesis | *Mvk/Hmgcs1/Mvd/Pmvk/Gm3571/Fdps/Hmgcr* |
| mmu05417 | Lipid and atherosclerosis | *Ldlr/Bid/Gm12346/Hsp90aa1/Pik3r3/Eif2ak3/Hspa1b/Ager/Mapk13/Hspa8/Nfatc3/Camk2g/Cyp1a1/Cybb/Hspa1a/Nox1/Gm15459/Il18/Hsp90b1/Chuk/Apob/Pparg/Itpr1/Hsp90ab1/Ly96/Nlrp3/Cyp2j9/Mapk11/Gm7232* |
| mmu04612 | Antigen processing and presentation | *Gm12346/H2-Q10/Hsp90aa1/Hspa1b/Lgmn/Hspa8/Hspa1a/Gm15459/*  *Ctss/H2-Ob/H2-M6-ps/H2-Oa/Hsp90ab1/Nfya/Gm7232/Pdia3* |
| mmu00190 | Oxidative phosphorylation | *Gm13340/Gm28661/Gm29216/mt-Co3/mt-Atp8/mt-Nd2/Uqcrb/*  *Gm10222/mt-Cytb/Atp5k/mt-Nd3/Ndufv2/mt-Nd4/Ndufb10/Cox6c/mt-Nd1/Uqcrfs1/mt-Atp6/Ndufa5/Ndufa3/mt-Co1/Gm28439/Atp5pb/*  *Gm10221/Uqcr10/mt-Co2/Gm10925/Cox6b1* |
| mmu05016 | Huntington disease | *Gm13340/Gm28661/Gm29216/mt-Co3/Gpx1/mt-Atp8/mt-Nd2/Uqcrb/*  *Gm10222/mt-Cytb/Dnah6/mt-Nd3/Ndufv2/mt-Nd4/Gpx3/Ndufb10/*  *Gm3756/Cox6c/Rb1cc1/Dctn6/mt-Nd1/Uqcrfs1/Tuba1a/mt-Atp6/Ndufa5/*  *Pparg/Polr2a/Itpr1/Ndufa3/Adrm1/Tubb2b/Tubb2a/mt-Co1/Gm28439/*  *Atp5pb/Dctn2/Uqcr10/Rcor1/mt-Co2/Dnah8/Gm10925/Cox6b1/Psmd11/*  *Klc2/Tbpl1/Vdac3-ps1/Psma1* |
| mmu04260 | Cardiac muscle contraction | *Gm13340/Gm28661/Gm29216/mt-Co3/Uqcrb/mt-Cytb/Cox6c/Uqcrfs1/*  *mt-Co1/Uqcr10/mt-Co2/Atp2a3/Cox6b1/Cacna2d2/Atp1a3* |
| mmu04972 | Pancreatic secretion | *Trpc1/Clca3b/Clca4a/Rab27b/Clca3a1/Prkcg/Itpr1/Adcy8/Clca2/Atp2a3/Ctrb1/Slc12a2/Slc4a2/Atp1a3/Atp2b1* |
| mmu04932 | Non-alcoholic fatty liver disease | *Bid/Gm13340/Gm28661/Gm29216/Pik3r3/Eif2ak3/mt-Co3/Mapk13/*  *Uqcrb/Socs3/mt-Cytb/Ndufv2/Ndufb10/Cox6c/Uqcrfs1/Ndufa5/*  *Pparg/Ndufa3/mt-Co1/Il6ra/Uqcr10/mt-Co2/Mapk11/Cox6b1/Prkag3* |
| mmu04146 | Peroxisome | *Mvk/Nos2/Far2/Prdx1/Acsl3/Gm21399/Hmgcl/Paox/Pex19/Mpv17l/Pmvk/Acaa1a/Pex5/Gm19680* |
| mmu05145 | Toxoplasmosis | *Ldlr/Nos2/Hspa1b/Mapk13/Hspa8/Hspa1a/Gm15459/Lama3/Birc3/Chuk/Lamc2/Pik3r5/H2-Ob/H2-Oa/Ly96/Mapk11/Gm7232* |
| mmu00380 | Tryptophan metabolism | *Kyat1/Cyp1a1/4930438A08Rik/Kmo/Gm39213/Kyat3/Ido1/Dld* |
| mmu04750 | Inflammatory mediator regulation of TRP channels | *Pik3r3/Mapk13/Camk2g/Asic5/Ptger4/Prkcg/Trpv3/Cyp4a32/Itpr1/Cyp4a31/Cyp4a29/Adcy8/Ppp1ccb/Cyp2j9/Pla2g4a/Mapk11* |
| mmu05012 | Parkinson disease | *Gm13340/Gm28661/Gm29216/Eif2ak3/mt-Co3/mt-Atp8/mt-Nd2/Uqcrb/*  *Camk2g/Gm10222/mt-Cytb/mt-Nd3/Ndufv2/mt-Nd4/Ndufb10/Gm3756/*  *Cox6c/mt-Nd1/Uqcrfs1/Tuba1a/mt-Atp6/Ndufa5/Lrrk2/Itpr1/Ndufa3/*  *Adrm1/Tubb2b/Tubb2a/mt-Co1/Uba52/Gm28439/Atp5pb/Uqcr10/mt-Co2/Slc39a10/Gm10925/Cox6b1/Psmd11/Klc2/Vdac3-ps1/Psma1/*  *Slc6a3* |
