# Supplementary material for: Inactivation of Atp7b Copper Transporter in Intestinal Epithelial Cells Is Associated with Altered Lipid Processing and Cell Growth Machinery Independent from Hepatic Copper Accumulation and Severity of Liver Histology
Source: Am J Pathol. 2025 Oct 16;196(2):407–27. doi: 10.1016/j.ajpath.2025.09.015 (PMC12881291; doi:10.1016/j.ajpath.2025.09.015)
Supplement: Supplemental Table S5 [file mmc13.docx]

**Supplemental Table S5. RNA-seq top 20 KEGG pathways and associated differentially expressed genes in IECs of 30-week *Atp7b*^ΔIEC^ mice (KEGG:** [**https://www.kegg.jp**](https://www.kegg.jp/)**).**

| **KEGG ID** | **Pathway Description** | **Gene Name** |
| --- | --- | --- |
| mmu04932 | Non-alcoholic fatty liver disease | *Casp3/Cox6a1/Ndufv1/Cyc1/Gm16418/4930481A15Rik/Pklr/Cox5b/Sdhb/Cox8a/Uqcrfs1/Cox7a1/Xbp1/Uqcr10/Prkag3/Cox5a/Ndufs8/Ndufa8/Uqcr11/Gm14023/Gm10053/Ndufb8/Casp7/Ndufs7/Bid/Ndufc1/Cox4i1/Tnf/Ndufb6/Cox7b/Uqcrc1/Gsk3a/Ndufb10/Cox6b1/Mlx/Ndufs5/Uqcrq/Ndufv3/mt-Co3/Sdhc/Ndufa3/Mlxip/Akt1/Sdhd/Gm12338/Ndufb9* |
| mmu05012 | Parkinson disease | *Casp3/Cox6a1/Ndufv1/Cyc1/Gm16418/4930481A15Rik/Psma2/Cox5b/Sdhb/Psmb4/Cox8a/Uqcrfs1/Cox7a1/Psmc3/Xbp1/Uqcr10/Cox5a/Atp5o/Psmb3/Gm11808/Psma5/Ndufs8/Atp5b/Ndufa8/Uqcr11/Gm10039/Gm10053/Keap1/Ndufb8/Psmd7/Ndufs7/Gm5406/Ndufc1/Atp5e/Cox4i1/Ndufb6/Cox7b/Atp5g3/Uqcrc1/Psmc5/Calm3/Ndufb10/Atp5g1/Slc39a1/Atp5a1/Cox6b1/Ndufs5/Psmb5/Sem1/Uqcrq/Uba1/Gm9774/mt-Nd3/Klc2/Ndufv3/mt-Co3/*  *Sdhc/Lrrk2/Ndufa3/Atp5h/Psmc2/Sdhd/Gm12338/Ndufb9/Ube2j2* |
| mmu00190 | Oxidative phosphorylation | *Cox6a1/Ndufv1/Cyc1/Gm16418/4930481A15Rik/Cox5b/Sdhb/Cox8a/Uqcrfs1/Cox7a1/Uqcr10/Cox11/Cox5a/Atp5o/Ndufs8/Atp5b/Ndufa8/Uqcr11/Gm10039/Gm10053/Ndufb8/Atp5k/Ndufs7/Ndufc1/Atp5e/Cox4i1/Ndufb6/Cox7b/Atp5g3/Uqcrc1/Ndufb10/Atp5g1/Atp5a1/Cox6b1/Ndufs5/Uqcrq/mt-Nd3/*  *Ndufv3/Ppa1/mt-Co3/Sdhc/Ndufa3/Atp5h/Sdhd/Gm12338/Ndufb9* |
| mmu05016 | Huntington disease | *Gpx2/Casp3/Cox6a1/Ndufv1/Cyc1/Gm16418/4930481A15Rik/Ap2m1/Psma2/Cox5b/Sdhb/Psmb4/Cox8a/Uqcrfs1/Cox7a1/Psmc3/Uqcr10/Cox5a/Dnal4/Atp5o/Psmb3/Psma5/Ndufs8/Atp5b/Ndufa8/Uqcr11/Gm10039/Gm10053/Ndufb8/Psmd7/Ndufs7/Gm5406/Ndufc1/Atp5e/Cox4i1/Gm11993/Ndufb6/Cox7b/Atp5g3/Uqcrc1/Psmc5/Ndufb10/Atp5g1/Actr10/Atp5a1/Hap1/Cox6b1/Ndufs5/Psmb5/Sem1/Uqcrq/Gm9774/mt-Nd3/Klc2/Ndufv3/mt-Co3/Sdhc/*  *Taf4/Ndufa3/Atp5h/Psmc2/Sdhd/Gm12338/Ndufb9/Mtor* |
| mmu05020 | Prion disease | *Casp3/Cox6a1/Ndufv1/Cyc1/Gm16418/4930481A15Rik/Psma2/Cox5b/Sdhb/Psmb4/Cox8a/Uqcrfs1/Cox7a1/Psmc3/Uqcr10/Cox5a/Atp5o/Psmb3/Psma5/Ndufs8/Atp5b/Ndufa8/Uqcr11/Gm14023/Gm10039/Gm10053/Ndufb8/Psmd7/Ndufs7/Gm5406/Ndufc1/Atp5e/Cox4i1/Tnf/Ndufb6/Cox7b/Atp5g3/Uqcrc1/Psmc5/Ndufb10/Atp5g1/Atp5a1/Cox6b1/Ndufs5/Psmb5/Sem1/Uqcrq/Gm9774/mt-Nd3/Klc2/Ndufv3/mt-Co3/Sdhc/Ndufa3/Atp5h/Psmc2/Sdhd/*  *Gm12338/Ndufb9* |
| mmu05415 | Diabetic cardiomyopathy | *Cox6a1/Ndufv1/Cyc1/Gm16418/Tbc1d4/4930481A15Rik/Gsr/Cox5b/Sdhb/Cox8a/Uqcrfs1/Cox7a1/Uqcr10/Cox5a/Atp5o/Ndufs8/Atp5b/Ndufa8/Uqcr11/Gm10039/Tgfbr1/Ndufb8/Ndufs7/Ndufc1/Atp5e/Cox4i1/Ndufb6/Cox7b/Atp5g3/Uqcrc1/Ppp1ca/Ndufb10/Atp5g1/Atp5a1/Cox6b1/Ndufs5/Uqcrq/mt-Nd3/Ndufv3/mt-Co3/Sdhc/Ndufa3/Atp5h/Akt1/Sdhd/Gm12338/Ndufb9/Mtor* |
| mmu04714 | Thermogenesis | *Cox6a1/Ndufv1/Cyc1/Gm16418/4930481A15Rik/Cox5b/Sdhb/Cox8a/Uqcrfs1/Cox7a1/Uqcr10/Cox11/Prkag3/Cox5a/Atp5o/Ndufs8/Atp5b/Ndufa8/Uqcr11/Gm10039/Ndufb8/Atp5k/Ndufs7/Ndufc1/Atp5e/Cox4i1/Ndufb6/Cox7b/Atp5g3/Uqcrc1/Ndufb10/Atp5g1/Atp5a1/Cox6b1/Ndufs5/Cox14/Uqcrq/mt-Nd3/Ndufv3/mt-Co3/Sdhc/Ndufa3/Atp5h/Sdhd/Gm12338/Ndufb9/Mtor* |
| mmu05208 | Chemical carcinogenesis - reactive oxygen species | *Cox6a1/Ndufv1/Cyc1/Gm16418/4930481A15Rik/Cox5b/Sdhb/Cox8a/Uqcrfs1/Cox7a1/Uqcr10/Cox5a/Atp5o/Ndufs8/Atp5b/Ndufa8/Uqcr11/Gm10039/Keap1/Ndufb8/Ndufs7/Ndufc1/Atp5e/Cox4i1/Ndufb6/Cox7b/Atp5g3/Uqcrc1/Ndufb10/Atp5g1/Atp5a1/Cox6b1/Ndufs5/Uqcrq/mt-Nd3/Ndufv3/mt-Co3/Slc26a6/Sdhc/Ndufa3/Atp5h/Akt1/Sdhd/Gm12338/Ndufb9* |
| mmu04260 | Cardiac muscle contraction | *Cox6a1/Slc8a1/Cyc1/Gm16418/4930481A15Rik/Hrc/Cox5b/Cox8a/Tpm3/Uqcrfs1/Tpm3-rs7/Cox7a1/Uqcr10/Cox5a/Uqcr11/Cox4i1/Cox7b/*  *Cacna2d1/Uqcrc1/Cox6b1/Fxyd2/Uqcrq/mt-Co3/Gm12338* |
| mmu03050 | Proteasome | *Psmb10/Psma2/Psmb4/Psmc3/Psmb3/Psma5/Psmb9/Psme2b/Psmd7/Psme1/Gm5406/Psmc5/Psmb8/Pomp/Psmb5/Sem1/Gm9774/Psmc2* |
| mmu04215 | Apoptosis - multiple species | *Casp3/Birc6/Bak1/Gm10053/Casp7/Bid/Gm26749* |
| mmu05017 | Spinocerebellar ataxia | *Cacna1a/Vldlr/Psma2/Psmb4/Psmc3/Xbp1/Psmb3/Psma5/Gm10053/Psmd7/Gm5406/Psmc5/Psmb5/Sem1/Gm9774/Psmc2/Akt1/Mtor* |
| mmu04640 | Hematopoietic cell lineage | *Il11/Dntt/Thpo/Cd5/Gm14023/Tnf/Siglech/Anpep/Il7/Cr1l/Cd4* |
| mmu04723 | Retrograde endocannabinoid signaling | *Ndufv1/Cacna1a/Gng5-ps/Ndufs8/Ndufa8/Ndufb8/Ndufs7/Ndufc1/Ndufb6/*  *Ndufb10/Ndufs5/mt-Nd3/Ndufv3/Ndufa3/Ndufb9* |
| mmu04130 | SNARE interactions in vesicular transport | *Sec22b/Stx18/Vamp5/Stx17/Stx8* |
| mmu04950 | Maturity onset diabetes of the young | *Neurod1/Pklr/Rfx6/Gck* |
| mmu04978 | Mineral absorption | *Slc8a1/Slc30a1/Slc9a3/Slc34a2/Fxyd2/Slc26a6* |
| mmu01250 | Biosynthesis of nucleotide sugars | *Gmppb/Gpi1/Gmds/Nans/Gck* |
| mmu00020 | Citrate cycle (TCA cycle) | *Sdhb/Aco2/Sdhc/Sdhd* |
| mmu03060 | Protein export | *Sec61b/Spcs2/Spcs1/Sec61a1* |
