# Supplementary material for: Inactivation of Atp7b Copper Transporter in Intestinal Epithelial Cells Is Associated with Altered Lipid Processing and Cell Growth Machinery Independent from Hepatic Copper Accumulation and Severity of Liver Histology
Source: Am J Pathol. 2025 Oct 16;196(2):407–27. doi: 10.1016/j.ajpath.2025.09.015 (PMC12881291; doi:10.1016/j.ajpath.2025.09.015)
Supplement: Supplemental Table S6 [file mmc14.docx]

**Supplemental Table S6. RNA-Seq top 20 KEGG pathways and associated differentially expressed genes in liver of 16-week *Atp7b*^-/-^ mice (KEGG:** [**https://www.kegg.jp**](https://www.kegg.jp/)**).**

| **KEGG ID** | **Pathway Description** | **Gene Name** |
| --- | --- | --- |
| mmu00280 | Valine, leucine and isoleucine degradation | *Acaa1b/Acadm/Mccc2/Acat1/Oxct1/Aldh6a1/Hadhb/Hmgcs2/Aacs/Hmgcll1/Hmgcl/Hadha/Aldh9a1/Acaa2/Hibadh/Hsd17b10/Pccb/Aldh1b1/Aldh3a2/Hmgcs1/Acat3/Aox2/Echs1/Hadh/Aox3/Bckdhb/Acadsb/Acads/Dld/Mcee/Hadhb-ps/Auh/Gm17244/Acaa1a/Bckdha/Acat2/Acad8/Oxct2b/Ehhadh/Mmut/*  *Bcat2/Aldh2/Aox1/Dbt/Aldh7a1/Agxt2* |
| mmu05208 | Chemical carcinogenesis - reactive oxygen species | *Cox6b2/Src/Gstm3/Gsta5/Prkd3/Cyp2e1/Nfe2l2/Gm8834/Sod2/4930481A15Rik/Uqcrfs1/Slc25a4/Atp5g1/Gm3776/Gm10039/Egfr/Atp5c1/Ikbkg/Ndufb10/Vdac2/Gm8566/Mapk13/Ndufv1/Cox5a/Cox7b/Cyp1a2/Gm10250/Atp5a1/Prkd2/Ndufb5/Pld1/Cat/Atp5h/Sdha/Ndufb4/Cyp1a1/Cox7c/Acp1/Cox4i1/Ndufv2/Sdhc/Hmox1/Vegfa/Uqcrc2/Ndufb11/Slc26a2/Atp5b/Ndufb9/Uqcr11/Uqcr10/Ndufa3/Gsta1/Atp5pb/Ndufb4c/Atp5j/Gstt1/Atp5o/Cyc1/Map2k2/mt-Nd2/*  *Sod1/Slc26a1/Ndufb8/Gm12338/Cyp2f2/Nox4/Ndufs2/Atp5e/Chuk/Ndufa4/*  *Uqcrc1/Cyp1b1/Sdhb/Ndufv3/Ndufa9/Ndufa12-ps/Ephx2/Gm16418/*  *ENSMUSG00000120425/Gm6665/Gm12337/Ndufa6/Ndufab1/Slc25a31/Ncf1/Atp5d/Nqo1/Ndufs7/Hras/Cox6c/Ndufs8/Akt2/Ndufs3/Uqcrb/Cox8a/Vdac1/Ephx4/Gstm2/Ndufa13/Ndufa5/Ndufa11/Cox7a2l/Mgst1/mt-Nd1/Cyba/*  *Ndufb7/Ndufs4/Bad/Atp5g2/Uqcrq/Atp5g3/Cox7a1/Sdhd/Ndufs1/Cox6a1/*  *Gm12499/Cox6b1/Cox5b/Ndufa2/Abl1/Cbr1/Gm15883/Ppif/Ndufc2/Ndufa1/Ndufab1-ps/Araf/Hgf/Vdac3/Gm2962/Ndufs6/Gm5436/Ndufs5-ps/mt-Nd3/*  *Vdac3-ps1/Gm4459/Ndufb2/Ndufa7/Gm16089/Cox5b-ps/Ndufa8/Ncf2/*  *Gsto1/Map3k14/Nfkbia/mt-Nd4/Pik3r3/Ephx1/Ndufs5/Pik3r2/mt-Cytb/*  *Atp5pb-ps/Gm12251/Gpr68/Cox7a2/Gstt3/Slc26a6/Ndufc1/Gstm4/Ndufa10/*  *Prkd1/Arnt/Ndufb3/As3mt/Gstm6/Met/Gstm2-ps1/Gm15283/Gm14388/*  *Ndufb6/Ptpn1/Gm13341/Braf/Gm14794* |
| mmu04146 | Peroxisome | *Acaa1b/Acsl1/Crot/Mlycd/Hacl1/Slc27a2/Dhrs4/Acox1/Sod2/Baat/Abcd3/Hsd17b4/Pex16/Pecr/Acsl3/Hmgcll1/Hmgcl/Amacr/Gm8566/Pipox/Xdh/Cat/Nudt7/Gstk1/Pex11a/Ech1/Phyh/Pex11b/Pex14/Pex5/Decr2/Sod1/Idh1/Scp2-ps2/Agxt/Pxmp4/Abcd4/Ephx2/Pex2/Acsl5/Paox/Agps/Mpv17l/Pex6/Mvk/*  *Pxmp2/Acaa1a/Mpv17l2/Pex11g/Scp2/Hao1/Ehhadh/4833413G10Rik/Acot8/Abcd1/Pex7/Hao2/Nudt19/Prdx1/Pex13/Mpv17/Gm19680/Pex5l/Far1/Idh2* |
| mmu00380 | Tryptophan metabolism | *Inmt/Acat1/Hadha/Gcdh/Aldh9a1/Cyp1a2/Cat/Dhtkd1/Afmid/Cyp1a1/Haao/Kynu/Aldh1b1/Aldh3a2/Acat3/Aldh8a1/Aox2/Echs1/Tdo2/Hadh/Maob/Kyat1/Cyp1b1/Aox3/Ido2/Aadat/Dld/Maoa/Acat2/Gm39213/Ehhadh/2900009J06Rik/Kyat3/Aoc1/Aldh2/Aox1/Aldh7a1* |
| mmu01212 | Fatty acid metabolism | *Acaa1b/Acsl1/Acadm/Elovl2/Elovl7/Acat1/Acadl/Elovl5/Acox1/Cpt1c/Hadhb/Hsd17b4/Acsl3/Hadha/Tecr/Acaa2/Fads1/Cpt2/Fads2/Scd2/Acat3/Echs1/Hacd3/Hadh/Scp2-ps2/Fasn/Acsl5/Acadsb/Acads/Hadhb-ps/Acaa1a/Acat2/*  *Scd1/Mcat/Scp2/Hacd4/Ehhadh/Acaca/H2-Ke6/Hacd1/Gm11451/Ppt2/*  *Acadvl/Scd3/Mecr/Gm5182/Elovl3/Elovl1/Rpp14* |
| mmu04512 | ECM-receptor interaction | *Hmmr/Spp1/Lamc3/Lamb3/Lamc2/Lama5/Col1a1/Itgb4/Thbs1/Itgav/Col6a3/Cd36/Itgb8/Col1a2/Col4a5/Col4a1/Lama2/Itgb6/Sdc4/Col4a2/Itga2/Lamc1/Lama3/Lamb2/Col4a3/Vtn/Cd44/Tnc/Col4a4/Itga3/Agrn/Frem2/Itga11/Col6a1/Col9a3/Frem1/Lama4/Col4a6/Itga6/Thbs4/Lamb1/Col6a2/Col6a6/Comp/Thbs3/Col9a2/Itga8/Itgb5/Thbs2/Itga9/Gm42604/Cd47/Sv2c/Fras1/Hspg2/Itga5/Itga4/Chad/Gp1ba* |
| mmu03440 | Homologous recombination | *Rad51/Rad54l/Eme1/Rad54b/Xrcc2/Bard1/Brca1/Blm/Mre11a/Rbbp8/Topbp1/Brip1/Babam1/Brcc3/Pold2/Rad50/Palb2/Rpa1/Top3a/Rpa2/Brca2/Sem1/Rad51c/Top3b/Pold1/Uimc1/Mus81/Atm/Ssbp1/Rad51d/Rpa3/Pold3/Abraxas* |
| mmu00071 | Fatty acid degradation | *Acaa1b/Acsl1/Acadm/Cyp4a31/Acat1/Acadl/Acox1/Cpt1c/Adh1/Hadhb/Adh4/Acsl3/Eci1/Hadha/Gcdh/Aldh9a1/Acaa2/Cpt2/Aldh1b1/Aldh3a2/Acat3/Echs1/Hadh/Cyp4a14/Adh5/Acsl5/Acadsb/Acads/Hadhb-ps/Acaa1a/Acat2/Adh7/*  *Gm39213/Ehhadh/Adh6-ps1/Cyp4a10/Aldh2/Acadvl/Aldh7a1* |
| mmu03460 | Fanconi anemia pathway | *Rad51/Fancd2/Atr/Eme1/Fancb/Fanci/Brca1/Fancg/Blm/Fance/Fanca/Polh/Faap24/Polk/Brip1/Cenps/Palb2/Rmi2/Rev1/Fancf/Slx4/Rpa1/Top3a/Fancm/Rpa2/Cenpx/Brca2/Rad51c/Poln/Pms2/Top3b/Fancl/Mus81/Fancc/Telo2/Rpa3/Eme2/Wdr48/Slx1b* |
| mmu04610 | Complement and coagulation cascades | *Fgb/Klkb1/Fgg/Cfh/Cd59a/Mbl1/Plat/Serpinf2/Fga/F2/C3ar1/Serpinc1/Bdkrb2/Itgax/Pros1/Cfhr2/Vtn/C8g/Kng1/Serpina1d/Plaur/Serpina1b/Serpina1a/Itgb2/F10/C5ar1/Plg/Proc/Gm16548/Serpind1/Gm8893/C1ra/Itgb2l/Gm16332/Cr2/F7/Cfhr3/Clu/Serpina5/Cfhr1/C4b/C8b/Cfd/F8/Serpine1/Cd55/Serpina1c/Masp1/Gm13449/F3/Cd46/F13a1/C4a/Hc/Thbd/Plau/C1qb/C1qc/Vsig4/F9/C1rb/Bdkrb1/C8a/Serpina1e* |
| mmu00051 | Fructose and mannose metabolism | *Akr1b3/Pfkl/Hkdc1/Akr1b10/Fbp2/Sord/Pfkp/Tpi1/Aldob/Khk/Pmm1/Fbp1/Pfkfb2/Hk1/Hk2/Gm29427/Gm47465/Pfkfb3/Aldoc/Gfus/Mpi/Pmm2/Tigar/Akr1b8/Gm12138/Akr1b7/Tpi-rs11/Gm6736/Pfkfb4/Pfkm/Gmppa/Fpgt* |
| mmu00650 | Butanoate metabolism | *Acat1/Oxct1/Bdh1/Hmgcs2/Aacs/Hmgcll1/Acsm4/Hmgcl/Hadha/L2hgdh/Acsm3/Hmgcs1/Acat3/Echs1/Hadh/Acads/Gm17244/Acat2/Oxct2b/Ehhadh/Acsm1/Aldh5a1/9330162012Rik/Acsm5* |
| mmu00190 | Oxidative phosphorylation | *Cox6b2/Atp6v0e2/4930481A15Rik/Uqcrfs1/Atp5g1/Gm10039/Atp5c1/Ndufb10/Ndufv1/Cox5a/Cox7b/Gm10250/Atp5a1/Ndufb5/Atp5h/Sdha/Ndufb4/Cox7c/Cox4i1/Ndufv2/Sdhc/Uqcrc2/Ndufb11/Atp5b/Ndufb9/Uqcr11/Uqcr10/Ndufa3/Atp5pb/Ndufb4c/Atp5j/Atp5o/Cyc1/mt-Nd2/Ndufb8/Gm12338/Ndufs2/Atp5e/*  *Ndufa4/Uqcrc1/Atp5l/Gm10053/Sdhb/Ndufv3/Ndufa9/Ndufa12-ps/Gm16418/*  *ENSMUSG00000120425/Gm12337/Ndufa6/Atp6v1f/Ndufab1/Atp5d/Ndufs7/Cox6c/Gm10221/Cycs/Ndufs8/Ndufs3/Uqcrb/Cox8a/Ndufa13/Atp5k/Ndufa5/Ndufa11/Cox7a2l/mt-Nd1/Ndufb7/Ndufs4/Atp5g2/Uqcrq/Atp5g3/Cox7a1/*  *Atp6v0a1/Sdhd/Ndufs1/Cox6a1/Cox6b1/Lhpp/Cox5b/Ndufa2/Ppa1/Ndufc2/Ndufa1/Ndufab1-ps/Gm2962/Ndufs6/Gm5436/Ndufs5-ps/mt-Nd3/Atp6v0d1/*  *Gm4459/Ndufb2/Ndufa7/Gm16089/Cox5b-ps/Ndufa8/mt-Nd4/Ndufs5/mt-Cytb/Atp5pb-ps/Gm12251/Atp6v0b/Atp6v0a4/Gpr68/Cox7a2/Atp6v0d2/*  *Ndufc1/Ndufa10/Ndufb3/Atp5l2-ps/Atp6v1b1/Atp6v1b2/Ndufb6/Gm13341/*  *Gm14794/Tcirg1/Cox10* |
| mmu04714 | Thermogenesis | *Cox6b2/Bmp8b/Acsl1/Ndufaf2/Pparg/Cpt1c/4930481A15Rik/Uqcrfs1/Atp5g1/Acsl3/Gm10039/Atp5c1/Ndufb10/Mapk13/Ndufv1/Cox5a/Prdm16/Creb3l2/Cox7b/Gm10250/Atp5a1/Ndufb5/Ndufaf1/Atp5h/Mgll/Slc25a20/Sdha/Rptoros/Ndufb4/Smarcc1/Cox7c/Cox4i1/Ndufv2/Sdhc/Zfp516/Cpt2/Adcy6/Uqcrc2/Ndufb11/Atp5b/Ndufb9/Uqcr11/Uqcr10/Slc25a29/Ndufa3/Atp5pb/Ndufb4c/Atp5j/Atp5o/Adcy7/Cyc1/mt-Nd2/Ndufb8/Gm12338/Gm26588/Fgfr1/Ndufs2/Atp5e/*  *Adcy1/Ndufa4/Uqcrc1/Ndufaf4/Creb3l1/Atp5l/Sdhb/Ndufaf8/Ndufv3/Ndufa9/Ndufa12-ps/Gm16418/ENSMUSG00000120425/Frs2/Gm12337/Acsl5/*  *Ndufa6/Cnr1/Ndufab1/Atp5d/Rps6kb2/Ndufs7/Hras/Cox6c/Ndufaf6/Gm10221/Ndufs8/Akt1s1/Prkaa1/Ndufs3/Uqcrb/Cox8a/Ndufa13/Atp5k/Ndufa5/Ndufa11/Cox7a2l/mt-Nd1/Rps6ka3/Smarca4/Ndufb7/Ndufs4/Atp5g2/Uqcrq/*  *Atp5g3/Cox7a1/Sdhd/Ndufs1/Cox6a1/Cox6b1/Mlst8/Creb3l3/Adcy3/Cox5b/Ndufa2/Smarcd2/Prkaca/Coa5/Ndufc2/Ndufa1/Creb3/Ndufab1-ps/Gm2962/*  *Creb5/Ndufs6/Gm5436/Ndufs5-ps/mt-Nd3/Gm4459/Ndufb2/Ndufa7/*  *Gm16089/Adcy8/Cox5b-ps/Adrb3/Ndufa8/mt-Nd4/Ndufs5/mt-Cytb/Atp5pb-ps/Gm12251/Prkag1/Adcy10/Lipe/Gpr68/Cox7a2/Cox19/Cox16/Gm12715/Ndufc1/Cox14/Ndufa10/Smarca2/Ndufb3/Adcy2/Atp5l2-ps/Smarcc2/Ndufb6/*  *Dpf1/Npr1/Prkab1/Gm13341/Kdm3b/Sirt6/Gm14794/Ndufaf5/Ndufaf3/Cox10* |
| mmu04110 | Cell cycle | *Mad2l1/Cdc20/Plk1/Bub1b/Trip13/Cdca5/Ttk/Cdk1/Chek2/Ccnb1/Aurkb/Espl1/Ccnb2/Sgo1/Ndc80/2700099C18Rik/Atr/Knl1/Cdkn2c/Ccna2/Dbf4/Bub1/Esco2/E2f2/Cdc6/Pkmyt1/Rbl1/Mtbp/Ccne2/Cdc45/Cdkn1a/Tgfb3/Tgfb2/Skp2/Gm13169/Wee1/Mdm2/Cdt1/Cdc25c/Ticrr/Ywhah/Ywhae/Chek1/Smc1b/Rb1/Mcm6/E330011O21Rik/Ppp2r1b/Gm13233/Cdc7/Skp1/Cdc23/Mcm2/Cdc25b/E2f1/Mcm5/Mcm4/Mcm7/Orc1/Ddx11/Tfdp1/Gadd45a/Mcm3/Sfn/Cdc26/Anapc4/Gm13244/Rad21/Ccnd3/Orc2/Cdkn2b/Ccnb1-ps/Ccnd2/Pttg1/*  *Gm4202/Ppp2ca/E2f3/Ywhag/Fbxo5/Ccnh/Hdac8/Cdc27/Abl1/Pcna/Rbx1/*  *Cdc25a/Cdc14a/Ywhaz/Cdc14b/Hdac1/Atm/Mad2l1bp/Anapc11/Ppp2r5d/Ep300/Orc6/Gm12856/Ywhaq/Anapc1/Anapc7/Cdk7/Anapc5/Cul1/Gm14287/Anapc10/Ppp2cb/Cdkn1b/Anapc2/Orc3/Mau2* |
| mmu05415 | Diabetic cardiomyopathy | *Cox6b2/Tgfbr2/Col1a1/Tgfb3/Tgfb2/Cybb/Col3a1/4930481A15Rik/Cd36/Col1a2/Uqcrfs1/Slc25a4/Atp5g1/Insr/Gys2/Gm10039/Atp5c1/Mpc2/Ndufb10/Pdk2/Mmp2/Vdac2/Agtr1a/Mapk13/Ndufv1/Cox5a/Ace/Ppp1ca/Cox7b/Gm10250/Atp5a1/Plcb4/Ndufb5/Atp5h/Sdha/Ndufb4/Cox7c/Cox4i1/Ndufv2/Sdhc/Cpt2/Uqcrc2/Pdk4/Ndufb11/Gfpt1/Agt/Atp5b/Ndufb9/Uqcr11/Uqcr10/Ndufa3/Atp5pb/Ndufb4c/Atp5j/Atp5o/Cyc1/mt-Nd2/Ndufb8/Gm12338/Plcb3/Ndufs2/Atp5e/*  *Ndufa4/Uqcrc1/Sdhb/Mpc1-ps/Ndufv3/Ndufa9/Ndufa12-ps/Gm16418/*  *ENSMUSG00000120425/Gm12337/Ndufa6/Ndufab1/Slc25a31/Ncf1/Atp5d/Ndufs7/Mpc1/Cox6c/Ndufs8/Akt2/Ndufs3/Uqcrb/Pdha1/Cox8a/Vdac1/Ndufa13/Ndufa5/Ndufa11/Cox7a2l/mt-Nd1/Cyba/Ndufb7/Ndufs4/Atp5g2/Uqcrq/*  *Atp5g3/Cox7a1/Pdk3/Sdhd/Ndufs1/Cox6a1/Cox6b1/Camk2b/Cox5b/Ndufa2/Ncf4/Ppif/Ndufc2/Ndufa1/Gfpt2/Ndufab1-ps/Vdac3/Gm2962/Ndufs6/*  *Gm5436/Ndufs5-ps/mt-Nd3/Vdac3-ps1/Gm4459/Ndufb2/Prkca/Ndufa7/*  *Gm16089/Cox5b-ps/Ndufa8/Ncf2/Pdhb/mt-Nd4/Pik3r3/Camk2a/Ndufs5/*  *Pik3r2/Ppp1cb/mt-Cytb/Atp5pb-ps/Gm12251/Nos3/Gpr68/Cox7a2/Ndufc1/*  *Ptpa/Ndufa10/Camk2g/Mmp9/Ndufb3/Ndufb6/Gm13341/Gm14794* |
| mmu04152 | AMPK signaling pathway | *Ccna2/Pfkl/Mlycd/Pck2/Gm13169/Pparg/Adra1a/Cpt1c/Cd36/Cftr/G6pc/Insr/Ppp2r2b/Gys2/Igf1r/Ppp2r1b/Gm13233/Igf1/Pck1/Creb3l2/Fbp2/Rptoros/Tbc1d1/Rab11b/Adipor2/Cab39l/Scd2/Pfkp/Gm8493/G6pc3/Rab8a/Crtc2/Creb3l1/Fasn/Gm13244/Ppp2r2a/Rps6kb2/Ppp2r3a/Srebf1/Eef2/Hmgcr/Akt2/Akt1s1/Prkaa1/Fbp1/Pfkfb2/Gm29427/Scd1/Ppp2ca/Cidea/Pfkfb3/Creb3l3/Acaca/Stk11/Gm11400/Creb3/Creb5/Lepr/Ppp2r3c/Pik3r3/Hnf4a/Eef2-ps2/Pik3r2/*  *Camkk2/Irs2/Prkag1/Scd3/Lipe/Adipor1/Ppp2r2c/Ppp2r5d/Gm19221/Sirt1/Acacb/Gm5182/Pfkfb4/Pfkm/Ppp2cb/Irs3/Prkab1/Rab10* |
| mmu00591 | Linoleic acid metabolism | *Cyp2c29/Cyp2c23/Cyp2c54/Cyp2c65/Cyp3a25/Pla2g2e/Cyp2e1/Cyp2j5/Cyp2c50/Cyp2c37/Cyp2c55/Cyp3a13/Cyp2c68/Cyp1a2/Pla2g2c/Cyp2c38/Pla2g5/Plb1/Plaat3/Cyp3a11/Pla2g12b/Pla2g4c/Pla2g4b/Cyp2c39/Cyp3a63-ps/*  *Pla2g4a/Cyp2c52-ps/Pla2g6/Cyp2j9/Cyp2c70/Cyp3a44/Pla2g12a/Cyp2c40* |
| mmu05144 | Malaria | *Icam1/Sdc2/Thbs1/Tgfb3/Tgfb2/Cd36/Klrb1/Vcam1/Tlr2/Itgb2/Gm16587/Itgb2l/Tnf/Il18/Thbs4/Tlr9/Comp/Pecam1/Sele/Thbs3/Hbb-bt/Cd40/Hgf/Itgal/Hba-a1/Thbs2/Hba-a2/Hbb-bs/Gypc/Ackr1/Gypa/Lrp1/Met/Ccl2* |
| mmu03030 | DNA replication | *Pole/Rfc5/Mcm6/Lig1/Pola1/Rnaseh2a/Fen1/Mcm2/Pold2/Pole2/Pole4/Mcm5/Mcm4/Mcm7/Mcm3/Rpa1/Rpa2/Rfc4/Pola2/Rnaseh2b/Rnaseh2c/Pcna/Pold1/Rfc2/Prim1/Ssbp1/Pole3/Rpa3/Pold3* |
