# Supplementary material for: Inactivation of Atp7b Copper Transporter in Intestinal Epithelial Cells Is Associated with Altered Lipid Processing and Cell Growth Machinery Independent from Hepatic Copper Accumulation and Severity of Liver Histology
Source: Am J Pathol. 2025 Oct 16;196(2):407–27. doi: 10.1016/j.ajpath.2025.09.015 (PMC12881291; doi:10.1016/j.ajpath.2025.09.015)
Supplement: Supplemental Table S7 [file mmc15.docx]

**Supplemental Table S7. RNA-Seq top 20 KEGG pathways and associated differentially expressed genes in liver of 16-week *Atp7b*^ΔIEC^ mice (KEGG:** [**https://www.kegg.jp**](https://www.kegg.jp/)**).**

| **KEGG ID** | **Pathway Description** | **Gene Name** |
| --- | --- | --- |
| mmu00260 | Glycine, serine and threonine metabolism | *Gm5096/Sdsl/Bhmt/Bhmt-ps1/Sardh/Shmt2/Gm15622* |
| mmu03030 | DNA replication | *Pole2/Mcm5/Rpa3/Mcm6/Pole4/Rnaseh2b* |
| mmu04010 | MAPK signaling pathway | *Stmn1/Kit/Efna1/Rps6ka5/Nfatc1/Mknk2/Mapkapk2/Taok1/Cacna1g/Jund/Dusp16/Il1r1/Egfr/Map2k6/Dusp10/Jun/Mapkapk5* |
| mmu05206 | MicroRNAs in cancer | *Stmn1/Efna1/Rps6ka5/Bcl2l11/Tnxb/Pim1/Zfpm2/Irs1/Abcb1b/Pak4/Rptor/Egfr* |
| mmu04928 | Parathyroid hormone synthesis, secretion and action | *Mafb/Lrp5/Adcy1/Slc9a3r1/Mmp25/Jund/Arhgef11/Egfr* |
| mmu05321 | Inflammatory bowel disease | *Nfatc1/Rora/Tlr2/Tbx21/Jun* |
| mmu00670 | One carbon pool by folate | *Mthfd2l/Shmt2/Mthfd1l* |
| mmu04150 | mTOR signaling pathway | *Lrp5/Slc38a9/Fnip2/Wnt4/Irs1/Prr5/Nprl2/Rptor/Grb10* |
| mmu04152 | AMPK signaling pathway | *Lipe/Ppp2r1a/Foxo3/Irs1/Ppargc1a/Scd3/Rptor/Fbp1* |
| mmu03440 | Homologous recombination | *Rad51/Rpa3/Uimc1/Xrcc3* |
| mmu03320 | PPAR signaling pathway | *Fabp5/Acsl3/Scd3/Fabp3-ps1/Apoa5/Acaa1a* |
| mmu04216 | Ferroptosis | *Acsl3/Atg7/Sat1/Slc40a1* |
| mmu04925 | Aldosterone synthesis and secretion | *Adcy1/Lipe/Dagla/Ldlr/Cacna1g/Gm45837* |
| mmu02010 | ABC transporters | *Abcc12/Abcb1b/Gm15821/Abcc3* |
| mmu05146 | Amoebiasis | *Adcy1/Lamc2/Tlr2/Rab7b/Il1r1/Gna14* |
| mmu04610 | Complement and coagulation cascades | *Gm16332/Gm13449/Fgg/Fgb/Fga/Cfd* |
| mmu00270 | Cysteine and methionine metabolism | *Gm5096/Sdsl/Bhmt/Bhmt-ps1/Gm15622* |
| mmu05142 | Chagas disease | *Adcy1/Ppp2r1a/Tlr2/Jun/Gm9845/Gna14* |
| mmu04659 | Th17 cell differentiation | *Nfatc1/Rora/Rara/Tbx21/Il1r1/Jun* |
| mmu04710 | Circadian rhythm | *Rora/Nfil3/Csnk1e* |
