# Supplementary material for: Inactivation of Atp7b Copper Transporter in Intestinal Epithelial Cells Is Associated with Altered Lipid Processing and Cell Growth Machinery Independent from Hepatic Copper Accumulation and Severity of Liver Histology
Source: Am J Pathol. 2025 Oct 16;196(2):407–27. doi: 10.1016/j.ajpath.2025.09.015 (PMC12881291; doi:10.1016/j.ajpath.2025.09.015)
Supplement: Supplemental Table S8 [file mmc16.docx]

**Supplemental Table S8. RNA-Seq top 20 KEGG pathways and associated differentially expressed genes in liver of 30-week *Atp7b*^-/-^ mice (KEGG:** [**https://www.kegg.jp**](https://www.kegg.jp/)**).**

| **KEGG ID** | **Pathway Description** | **Gene Name** |
| --- | --- | --- |
| mmu04610 | Complement and coagulation cascades | *Plat/Clu/Klkb1/Itgax/Bdkrb2/Serpina1d/C8g/Proc/Serpina1b/F7/Plaur/Vwf/Cfhr3/C3ar1/Gm16332/Plg/Serpinc1/F3/Vtn/Mbl1/Cd59b/C7/Itgam/Serpina1a/F2/Serpind1/Serpine1/Pros1/Cfhr2/Cd59a/F10/F2r/Gm16548/C5ar1/Serpinf2/Cfhr1/Itgb2/Cr2/Serpina1c/F13b/Plau/Procr/Gm8893/Masp2/A2m/Serpina5/Kng1/B430119L08Rik/Thbd/Cfh/C1ra/C1qb/C8b/Fga/F9/Fgb/Kng2/Fgg/Cfi/C2/C1qc/Serping1/C1qa/Mbl2/Cpb2/F13a1/Bdkrb1/Serpina1e/C1s2/Cd46/Cr1l/Cfd/F12/Tfpi/Cd55/Masp1* |
| mmu04512 | ECM-receptor interaction | *Spp1/Lamc3/Lamc2/Lama5/Itgb8/Col1a1/Col6a3/Itga3/Itgb4/Col1a2/Itga8/Thbs1/Itgb6/Hmmr/Col4a6/Lamb2/Tnc/Col4a2/Col4a4/Col4a1/Col6a2/Itga2/Lama2/Col6a1/E030013I19Rik/Vwf/Cd44/Thbs2/Vtn/Thbs3/Agrn/Gm42604/Col4a3/Col4a5/Lama4/Frem1/Col9a3/Itga11/Col6a6/Lamc1/Lamb1/Itgav/Itga9/Itgb7/Tnr/Comp/Frem2/Lamb3/Sdc4/Col9a2/Lama3/Itga6/Fras1/Hspg2/Itgb5/Itga4/Thbs4/Itga7/Col6a4/Tnn/Lama1/Cd36/Col6a5/Tnxb/Fn1/Sv2a/Col2a1/Itgb1/Sv2c/Gp9* |
| mmu04146 | Peroxisome | *Baat/Pecr/Acsl1/Sod2/Hacl1/Slc27a2/Pipox/Gm8566/Gstk1/Agxt/Hmgcll1/Sod1/Paox/Abcd3/Cat/Mpv17l/Acsl5/Ech1/Pex2/Dhrs4/Idh1/Decr2/Ephx2/Pex5l/Far1/Acaa1b/Mlycd/Acsl4/Pex14/Gm19680/Pex11a/Pxmp2/Pex7/Pex11b/Agps/Mvk/Phyh/Hao1/Nudt7/Acox1/Pex6/Pex16/Hao2/Hmgcl/Scp2-ps2/Amacr/Pex26/4833413G10Rik/*  *Pex13/Pmvk/Xdh/Acnat1/Acot8/Crot/Pex3/Abcd2/Pex10/Nos2/Abcd4/Acsl3/Eci3/Nudt12/Pex5/Hsd17b4/Pxmp4/Scp2/Dao/Acnat2/Pex11g/Acaa1a/Crat/Acox3* |
| mmu00280 | Valine, leucine and isoleucine degradation | *Aldh9a1/Hibadh/Mccc2/Oxct1/Hmgcll1/Aldh6a1/Acat3/Mcee/Pccb/Aox2/Hmgcs2/Acaa2/Acat1/Aldh1b1/Aacs/Acaa1b/Dbt/Acadsb/Acad8/Dld/Acat2/Hsd17b10/Hadhb-ps/Mmut/4930438A08Rik/Hadha/Bcat2/Bckdha/Echs1/Hadhb/Abat/Hmgcl/Auh/Hadh/Oxct2b/Pcca/Aldh2/Ivd/Aox3/Acadm/Acads/Aox4/Gm17244/Bckdhb/Aldh7a1/Bcat1/Acaa1a* |
| mmu05415 | Diabetic cardiomyopathy | *Tgfb3/Tgfb2/Col1a1/Col3a1/Tgfbr2/Ace/Col1a2/Mmp2/Cox6b2/Mapk13/Slc25a4/Cybb/Pdk4/Pdk3/Agt/mt-Nd3/Agtr1a/Pdha1/Gm10925/Ncf1/Ndufb5/Ndufb10/Ndufv3/*  *Atp5a1/Ndufb9/Gm10250/4930481A15Rik/Atp5h/Atp5g1/Ndufa3/Ndufa5/Sdhc/Ndufa9/Atp5j/mt-Nd1/Gm10039/Smad3/Ndufb11/Ndufs7/Atp5c1/Sdhb/Mpc2/Cpt2/*  *Uqcrc2/mt-Cytb/Sdha/Cyba/Ncf4/Ncf2/Ndufa6/Vdac1/G6pdx/Pik3cd/Plcb4/Uqcrb/*  *Pik3r3/Cox8a/Plcb2/Ndufs8/Uqcrc1/Ppp1cb/Ndufs1/Gfpt2/Ndufa12-ps/Mpc1-ps/*  *Gpr68/Uqcrfs1/Insr/Cox7c/Ndufv1/Cox6c/Akt2/Atp5d/Cyc1/Ndufa4/mt-Nd2/mt-Nd4/*  *Mpc1/Pdhb/Ndufa10/Cox5a/Ndufs2/Atp2a3/Ndufb7/Ndufv2/Prkcb/Gm12338/Camk2a/Ndufs3/Gm2962/Atp5o/Pten/Rac2/Ndufb8/Ndufb3/Ppara/Cox4i1/Atp5e/Prkcg/Atp5pb/Uqcr11/Ndufa1/Cox5b/Cox7b/Gm4459/Ndufab1-ps/Ndufa13/Sdhd/Ndufa11/*  *Vdac2/Atp5g2/Cox5b-ps/Uqcrq/Atp5g3/Pdk2/Atp5b/Prkcd/Ndufc1/Uqcr10/Vdac3/*  *Gm16418/Akt3/Ndufb4/Gm12337/Ndufab1/Cox6a1/Vdac3-ps1/Ndufs5/Ndufa2/*  *Ndufb6/Atp5pb-ps/Ndufc2/Plcb3/Ndufs6b/Ndufs6/Cox6b1/Gm16089/Ppif/Gys2/*  *Gm8437/Camk2d/Ppp1ca/Cox7a1/Cox4i2/Pik3r2/Gm28437/Ndufb2/Gm14794/Gm10175/mt-Co1/Ctsd/Ndufs4/Cox7a2/Cd36/Slc2a1/Tgfbr1/Ndufa7/Gm7591/Prkcz/*  *Gm15694/mt-Co3/Gm12251/Mapk11/Gm28661/Pik3r1/Ryr2/Gm13339/Gys1/Tnni3/*  *ENSMUSG00000120425/Gm6123/Parp1/Gm5436/Gm11966/Gm6969/Ndufa8/Mmp9/Slc25a5/Gm6444/Gm29216/Cox6a2/Ren1* |
| mmu05020 | Prion disease | *Casp12/Apaf1/Klc3/Tuba8/Ncam1/Cacna1b/Cav2/Cox6b2/Mapk13/Slc25a4/Cybb/Tubb6/Tuba1a/C8g/Gm8566/Itpr3/Cacna1c/Sod1/mt-Nd3/C7/Gm10925/Ncf1/Ndufb5/*  *Tubb2a/Tuba1b/Ndufb10/Ndufv3/Atf4/Atp5a1/Ndufb9/Gm10250/4930481A15Rik/Creb5/Atp5h/Cav1/Tubb4b/Mapk3/Atp5g1/Psmc5/Ndufa3/Kif5c/Tubb2b/Ndufa5/Sdhc/Ndufa9/Grin2d/Atp5j/Csnk2b/mt-Nd1/Gm10039/Ndufb11/Ndufs7/Lamc1/Atp5c1/*  *Creb3l1/Sdhb/Gm5406/Uqcrc2/mt-Cytb/Sdha/Cyba/Ncf4/Ncf2/Ryr1/Tubb3/Ndufa6/*  *Ppp3cc/Vdac1/Pik3cd/Uqcrb/Tubb4a/Pik3r3/Tnf/Cox8a/Ndufs8/Hspa5/Uqcrc1/*  *Hspa1b/Ndufs1/Ndufa12-ps/Hspa8/Gpr68/Uqcrfs1/Tuba4a/Tubb5/Cox7c/Ndufv1/*  *Cox6c/Atp5d/Cyc1/Ndufa4/mt-Nd2/mt-Nd4/Ndufa10/Cox5a/Psmd12/Adrm1/Ndufs2/*  *Tuba1c/C1qb/Ndufb7/Ndufv2/Gm12338/Gm15459/Gm3756/Ndufs3/Tubb4b-s1/Bax/*  *Gm2962/Psmd6/Atp5o/Stip1/C8b/Rac2/Ndufb8/Ndufb3/Klc2/Cox4i1/Creb3l4/Atp5e/*  *Psmc1/Atp5pb/Uqcr11/Ndufa1/Cox5b/Cox7b/Gm4459/Psmd3/Ndufab1-ps/Gm8355/*  *Grin2c/Cacna1s/Ndufa13/Psmb2/Sdhd/Ndufa11/Vdac2/Atp5g2/Cox5b-ps/Uqcrq/*  *Psmc6/Atp5g3/Il1b/Psmc2/Prkaca/Atp5b/Prkcd/Ndufc1/Uqcr10/Vdac3/Gm16418/*  *C1qc/Cav3/Prnp/Psmd4/C1qa/Ndufb4/Gm12337/Psmb6/Casp3/Ndufab1/Cox6a1/Klc1/Vdac3-ps1/Ndufs5/Ndufa2/Ndufb6/Grin2b/Gm6467/Atp5pb-ps/Ndufc2/Psma4/*  *Ndufs6b/Ndufs6/Psmb3/Psma3/Cox6b1/Psmb1/Gm16089/Sem1/Ppif/Gm8437/Itpr2/*  *Mcu/Cox7a1/Cox4i2/Gm10053/Tuba3a/Pik3r2/Gm28437/Ndufb2/Gm14794/Gm10175/mt-Co1/Psma7/Grin1/Ndufs4/Gm13835/Cycs/Hspa2/Eif2ak3/Mapk1/Cox7a2/Klc4/*  *Prkacb/Gm14150/Gm4950/Creb3l2/Psmc3/Ndufa7/Gm7591/Psmd8/Psmd9/Kif5b/*  *Bad/4930447K03Rik/Gm15694/Psma2/mt-Co3/Gm12251/Mapk11/Gm28661/Pik3r1/*  *Ryr2/Gm13339/A730017L22Rik/Psma6/Psmb4/Notch1/Tuba5-ps/Psma5/*  *ENSMUSG00000120425/Creb3l3/Gm5436/Gm11966/Gm49450/Gm6969/Ndufa8/*  *Psmd2/Tmem259/Gm44040/Slc25a5/Gm6444/Gm29216/Cox6a2/Psma5-ps* |
| mmu05208 | Chemical carcinogenesis - reactive oxygen species | *Src/Gstm3/Prkd3/Gm8834/Cox6b2/Sod2/Mapk13/Slc25a4/Gsto1/Cyp1b1/Nfe2l2/Vegfa/Gm8566/Map3k14/Cyp2e1/Gstm2/Pld1/Cyp1a1/Sod1/mt-Nd3/Gm6665/Cat/*  *Ephx4/Hmox1/Gm10925/Ncf1/Ndufb5/Gsta5/Nqo1/Slc26a1/Ndufb10/Map2k2/Ndufv3/Gstt1/Atp5a1/Ephx2/Ndufb9/Gm10250/4930481A15Rik/Atp5h/Mapk3/Atp5g1/Ndufa3/As3mt/Chuk/Jun/Ndufa5/Sdhc/Ndufa9/Atp5j/Gsta1/mt-Nd1/Gm10039/Ndufb11/*  *Ndufs7/Atp5c1/Sdhb/Uqcrc2/mt-Cytb/Sdha/Cyba/Gm3776/Ncf2/Ndufa6/Vdac1/*  *Pik3cd/Ephx1/Uqcrb/Pik3r3/Cox8a/Hgf/Ndufs8/Uqcrc1/Ndufs1/Ndufa12-ps/Ikbkg/*  *Nox4/Gpr68/Uqcrfs1/Cox7c/Acp1/Ndufv1/Prkd1/Arnt/Cox6c/Akt2/Atp5d/Cyc1/*  *Ndufa4/mt-Nd2/mt-Nd4/Ndufa10/Cox5a/Ndufs2/Prkd2/Ndufb7/Ndufv2/Hras/*  *Gm12338/Ndufs3/Gm2962/Atp5o/Pten/Ndufb8/Nras/Ndufb3/Cox4i1/Atp5e/Atp5pb/Uqcr11/Ndufa1/Cox5b/Cox7b/Gm4459/Ndufab1-ps/Ndufa13/Sdhd/Ndufa11/Vdac2/*  *Atp5g2/Cox5b-ps/Uqcrq/Atp5g3/Atp5b/Prkcd/Ndufc1/Uqcr10/Vdac3/Cyp1a2/*  *Gm16418/Cyp2f2/Akt3/Gstm2-ps1/Ndufb4/Gm12337/Ndufab1/Cox6a1/Vdac3-ps1/*  *Ndufs5/Ndufa2/Ndufb6/Atp5pb-ps/Met/Ndufc2/Pld2/Ndufs6b/Lpo/Ndufs6/Cox6b1/*  *Gm16089/Ppif/Gstm7/Gm8437/Mgst1/Cox7a1/Cox4i2/Pik3r2/Gm28437/Ndufb2/Gm14794/Gm10175/mt-Co1/Ndufs4/Mapk1/Gsta2/Cox7a2/Gm4956/Araf/Fos/Gm12499/*  *Ndufa7/Gm7591/Bad/Gm15694/mt-Co3/Gm12251/Mapk11/Gm28661/Pik3r1/*  *Gm13339/ENSMUSG00000120425/Ephx3/Gm5436/Gm11966/Gstt3/Gm6969/Ndufa8/Keap1/Gstm6/Gm14388/Abl1/Slc26a2/Slc25a5/Gm6444/Gm29216/Egf/Cox6a2* |
| mmu04151 | PI3K-Akt signaling pathway | *Spp1/Lamc3/Lamc2/Vegfb/Lama5/Pdgfd/Ywhah/Itgb8/Col1a1/Col6a3/Ppp2r2b/Itga3/Itgb4/Col1a2/Itga8/Erbb2/Pdgfb/Cdkn1a/Pdgfa/Thbs1/Itgb6/Col4a6/Ghr/Lamb2/Tnc/Col4a2/Col4a4/Col4a1/Pdgfc/Pdgfra/Gnb5/Vegfa/Igf1r/Col6a2/Osmr/Flt3/G6pc3/Itga2/Gnb1/Lama2/Efna4/Col6a1/Pkn3/Pdgfrb/Rptoros/E030013I19Rik/Vwf/Pck2/Tlr2/Ywhaz/Thbs2/Jak3/Vtn/Brca1/Ccnd2/Thbs3/Angpt1/Bcl2/Col4a3/Fgf1/Igf1/Col4a5/Lama4/Map2k2/Fgfr1/Csf3r/Atf4/Fgfr3/Il6ra/Ppp2r1b/Il3ra/Creb5/Col9a3/Trp53/Areg/F2r/Itga11/Efna2/Mapk3/Efna3/Ifnar2/Chuk/Col6a6/Ngf/Lpar1/Stk11/Gm13169/Lamc1/Ntf5/G630022F23Rik/Lamb1/Itgav/Creb3l1/Kitl/Gm15998/Itga9/Itgb7/Csf1r/Pik3ap1/Pik3cd/Tnr/Rxra/Pik3r3/Hgf/Comp/Igf2/Fgfr2/Kdr/Gng2/Il7/Lamb3/Vegfc/Ikbkg/Eif4b/Pik3r5/Ddit4/Il2rg/Syk/Insr/Col9a2/Il4ra/Them4/Akt2/Ppp2r1a/Gnb4/Gng8/Gm13233/Lama3/Ccne1/Ppp2ca/Pik3cg/Chrm1/Ppp2r5e/Hras/Prkaa1/Pten/Itga6/Prlr/Cdk6/Nras/Epor/Creb3l4/Cdc37/Itgb5/Mdm2/Ppp2r2a/Ppp2r5d/Rps6kb2/Bcl2l11/Mlst8/Erbb4/Magi2/Tgfa/Itga4/Gm8493/Thbs4/Ntrk2/Fgf7/Fgfr4/Crtc2/Lpar4/Fgf2/Gm4202/Tlr4/Akt3/Itga7/Col6a4/Flt4/Rbl2/Angpt4/Met/Il4/Ppp2r2c/Gm2446/Pgf/Cdk2/Ywhae/Hsp90b1/Osm/Gm13244/Ppp2r3a/Gys2/Ntf3/Csf1/Pck1/Pik3r2/Rps6kb1/Il7r/Prkaa2/Mapk1/Tnn/Lama1/Gngt1/Sgk2/Col6a5/Fgf18/Creb3l2/Vegfd/Il2ra/Gm7327/Bad/Ppp2r3d/Tnxb/Ccne2/Ntrk1/Nr4a1/Mcl1/Pik3r1/Fn1/Efna1/Ccnd3/Jak1/Gys1/Col2a1/Itgb1/Creb3l3/Fgf21/Gnb2/Il2rb/Lpar5/Eif4e2/Gm12217/Cdkn1b/Gng12/Ywhaq-ps3/Myb/Egf/*  *Ifnar1/Gng11/Epha2/Bcl2l1* |
| mmu04918 | Thyroid hormone synthesis | *Gpx2/Atp1a2/Alb/Adcy7/Gpx3/Duox2/Adcy8/Duox1/Ttr/Itpr3/Adcy2/Atp1b2/Adcy3/Adcy1/Gpx7/Duoxa2/Atp1a3/Atp1b3/Atf4/Serpina7/Creb5/Adcy6/Gm13415/Creb3l1/Asgr2/Slc5a5/Plcb4/Ttf2/Plcb2/Hspa5/Iyd/Atp1a4/Prkcb/Tg/Creb3l4/Prkcg/Prkaca/Gpx1/Plcb3/Gpx8/Hsp90b1/Slc26a4/Itpr2/Gpx6/Asgr1/Prkacb/Pdia4/Creb3l2/Adcy5/Adcy9/Creb3l3/Lrp2/Gm44040/Canx* |
| mmu04932 | Non-alcoholic fatty liver disease | *Cox6b2/Mapk13/Cyp2e1/Mlxipl/Cebpa/Tnfrsf1a/Ndufb5/Ndufb10/Ndufv3/Atf4/Pklr/Adipor2/Ndufb9/Il6ra/4930481A15Rik/Srebf1/Prkab2/Ndufa3/Jun/Ndufa5/Sdhc/Ndufa9/Ndufb11/Ndufs7/Mlx/Nr1h3/Sdhb/Uqcrc2/mt-Cytb/Sdha/Ndufa6/Pik3cd/Rxra/Uqcrb/*  *Pik3r3/Tnf/Cox8a/Ndufs8/Uqcrc1/Ndufs1/Ndufa12-ps/Gpr68/Uqcrfs1/Insr/Cox7c/*  *Ndufv1/Cox6c/Akt2/Cyc1/Ndufa4/Ndufa10/Cox5a/Ndufs2/Ndufb7/Ndufv2/Gm12338/*  *Ndufs3/Prkag2/Prkaa1/Bax/Gm2962/Ndufb8/Ndufb3/Bid/Ppara/Cox4i1/Cdc42/Uqcr11/Bcl2l11/Ndufa1/Cox5b/Cox7b/Gm4459/Ndufab1-ps/Ndufa13/Gm20257/Sdhd/*  *Ndufa11/Cox5b-ps/Uqcrq/Mlxip/Il1b/Ndufc1/Uqcr10/Gm16418/Akt3/Ndufb4/Casp3/*  *Ndufab1/Cox6a1/Xbp1/Ndufs5/Ndufa2/Ndufb6/Ndufc2/Ndufs6b/Ndufs6/Cox6b1/Gm16089/Gm8437/Cox7a1/Cox4i2/Gm10053/Pik3r2/Gm28437/Ndufb2/mt-Co1/Ndufs4/*  *Cycs/Eif2ak3/Prkaa2/Socs3/Cox7a2/Fos/Ndufa7/Gm15694/mt-Co3/Gm12251/*  *Mapk11/Gm28661/Pik3r1/Map3k11/Prkab1/ENSMUSG00000120425/Gm6969/*  *Ndufa8/Lepr/Gm6444/Gm29216/Cox6a2* |
| mmu04060 | Cytokine-cytokine receptor interaction | *Cxcl14/Eda2r/Bmp8b/Tnfrsf10b/Tgfb3/Cx3cr1/Tgfb2/Cx3cl1/Tgfbr2/Csf2ra/Il17re/Clcf1/Lifr/Il1rap/Ghr/Ifnlr1/Csf2rb/Ccr2/Tnfrsf19/Ltb/Osmr/Lif/Inhbb/Bmp10/Ccl22/Ccr7/Eda/Tnfrsf12a/Gdf10/Cxcl5/Il10rb/Il17d/Ccr1/Csf2rb2/Cxcr4/Gdf6/Tnfrsf1a/Xcr1/Tnfrsf11a/Inhbc/Tnfrsf13b/Acvrl1/Ackr3/Ccl27a/Ifngr1/Csf3r/Ccl20/Cxcl16/Tnfrsf11b/Il18/Il6ra/Gm20319/Bmp6/Il3ra/Cxcl10/Il22ra1/Ifnar2/Ccr5/Ngf/Il21r/Gdf3/Il10ra/Il1rl2/Il33/Csf1r/Cd40/Inhba/Ccl9/Tnf/Il7/Pf4/Tnfsf13b/Cxcr3/Il36g/Il2rg/Il11ra1/Il4ra/Acvr2b/Il15ra/Thpo/Ifngr2/Il17rc/Il27ra/Ccr10/Inha/Prlr/Cxcl1/Epor/Il12b/Il23a/Tnfsf14/Relt/Il17rb/Il1r1/Tnfsf13/Ccl7/Acvr1b/Tnfsf8/Gdf11/Il1b/Il11/Ccl2/Il1r2/Bmp3/Cxcr1/Cntfr/Tnfrsf9/Acvr2a/Tnfsf10/Il4/Cxcr6/Stard5/Osm/Tnfsf9/Csf1/Cxcl2/Il17ra/Cxcl17/Bmp2/Il7r/Tnfrsf8/Ctf1/Acvr1c/Cxcl9/Bmp8a/Tnfrsf4/Tgfbr1/Ccr8/Cxcr2/Il2ra/Gdf7/Tnfsf12/Tnfsf11/Ccr3/Ccl6/Ccl12/Gm41609/Ccl8/Tnfrsf13c/Ccl4/Crlf2/Cxcl12/Cxcr5/Edar/Il9r/Tnfrsf21/Il2rb/Cers1/Ccr4/Il13ra1/Lepr/Ifnar1/Tnfrsf18* |
| mmu04814 | Motor proteins | *Myo7b/Kifc1/Dynlt1f/Kif20b/Kif12/Dync2li1/Klc3/Kif22/Kif2c/Tuba8/Kif1a/Kif18a/Cenpe/Dnah1/Kif20a/Kifc5b/Kifc3/Kif11/Tubb6/Kif18b/Myo5c/Bicdl2/Tuba1a/Kif4/Myo1f/Myh14/Myh10/Myl12a/Kif9/Myo15b/Myl12b/Myo18b/Myh9/Tpm4/Acta1/Dnah12/Dynlt1a/Kif15/Gm5526/Myo1c/Myo9b/Myo5a/Tubg1/Tubb2a/Tuba1b/Myo1g/Kif1c/Tpm3/Kif26b/Tubb4b/Dynlt1-ps1/Kif5c/Dnah9/Tubb2b/Kif24/Dnah7b/Myo16/Dync1li2/Kif14/Dynll2/Tube1/Kif21b/Kif3b/Dync1i2/Kif3a/Myo5b/Tubb3/Dnal1/Dnah10/Tubb4a/Dnai1/Tubg2/Kif23/Actc1/Kif27/Kifap3/Tuba4a/Acta2/Tubb5/Dnah5/Myo3b/Kif7/Gm4961/Dnai2/Dnah6/Tuba1c/Gm3756/Tubb4b-ps1/Myo1b/Kifc2/Klc2/Myo7a/Myo10/Kif6/Dnah17/Dctn1/Tpm2/Dync1li1/Tnnc1/Actr1a/Myh7b/Klc1/Kif3c/Myo19/Dynlrb1/Kif26a/Myl6b/Dnah11/Dync1h1/Kif17/Dnah2/Myh13/Tuba3a/Myh3/Dnah7a/Dnali1/Dynll1/Dnai4/Dynlt1c/Dnah8/Klc4/Myo1a/Tnni2/Dync2h1/Gm14150/Capzb/Bicdl1/Hook3/Myh15/Kif19a/Myl9/Kif5b/4930447K03Rik/Tnnt1/Kif2a/Myo15/Tpm3-rs7/Tuba5-ps/Tnni3/Dnal4/Gm49450/Dynlrb2/Dynlt3/Actr10/*  *Myo6/Mylpf/Kif21a/Myh11* |
| mmu00380 | Tryptophan metabolism | *Aldh8a1/Afmid/Cyp1b1/Inmt/Haao/Aldh9a1/Maob/Dhtkd1/Gcdh/Cyp1a1/Kyat3/Acat3/Cat/Aox2/Ido2/Aoc1/Acat1/Gm39213/Aldh1b1/Dld/Kyat1/Acmsd/Acat2/Aadat/Kynu/4930438A08Rik/Hadha/Tdo2/Echs1/Hadh/Aldh2/Aox3/Cyp1a2/2900009J06Rik/Maoa/Aox4/Aldh7a1/Kmo* |
| mmu05140 | Leishmaniasis | *Tgfb3/Tgfb2/Mapk13/Cybb/H2-DMb1/H2-Eb1/H2-Aa/Tab1/H2-Ab1/Tlr2/Itgam/Ncf1/*  *Ifngr1/Marcksl1/H2-DMb2/H2-DMa/Mapk3/Itgb2/Jun/Cyba/Ncf4/Ncf2/Eef1a2/H2-Oa/*  *Tnf/Fcgr3/Fcgr1/Ifngr2/Prkcb/H2-Ob/Stat1/H2-Eb2/Il12b/Eef1a1/Irak1/Itga4/Map3k7/*  *Il1b/Tlr4/Gm5869/Nos2/Myd88/Il4/Gm9512/Gm13456/Fcgr4/Mapk1/Fos/Gm6789/Eef1a1-ps1/Mapk11/Cr1l/Jak1/Itgb1/Gm5822/Gm6170/Ptgs2/Irak4/Traf6/Gm6548* |
| mmu05146 | Amoebiasis | *Lamc3/Tgfb3/Lamc2/Serpinb6a/Tgfb2/Lama5/Col1a1/Col3a1/Col1a2/Col4a6/Lamb2/Col4a2/Col4a4/Col4a1/C8g/Lama2/Cd14/Serpinb6b/Tlr2/Adcy1/Itgam/Col4a3/Cd1d1/Col4a5/Lama4/Rab7b/Gna15/Rab5b/Itgb2/Arg1/Actn1/Lamc1/Lamb1/Pik3cd/Plcb4/Pik3r3/Tnf/Plcb2/Arg2/Lamb3/Serpinb9e/Serpinb9/Vcl/Lama3/Gna14/Prkcb/Serpinb9b/C8b/Cxcl1/Il12b/Prkcg/Il1r1/Il1b/Prkaca/Tlr4/Il1r2/Casp3/Nos2/Rab5c/Plcb3/Cxcl2/Muc2/Pik3r2/Serpinb9f/Lama1/Prkacb/Gm13991/Hspb1/Pik3r1/Fn1/Gna11* |
| mmu05016 | Huntington disease | *Gpx2/Apaf1/Tgm2/Klc3/Tuba8/Dnah1/Gpx3/Cacna1b/Cox6b2/Sod2/Slc25a4/Tubb6/Tuba1a/Gm8566/Polr2i/Dlg4/Gria3/Sod1/mt-Nd3/Dnah12/Gpx7/Tfam/Gm10925/*  *Ndufb5/Tubb2a/Tuba1b/Ndufb10/Ndufv3/Atp5a1/Ndufb9/Gm10250/4930481A15Rik/Creb5/Trp53/Atp5h/Tubb4b/Atp5g1/Psmc5/Ndufa3/Kif5c/Dnah9/Tubb2b/Dnah7b/Ift57/Ndufa5/Sdhc/Ndufa9/Atp5j/mt-Nd1/Gm10039/Ndufb11/Ndufs7/Atp5c1/Creb3l1/*  *Sdhb/Gm5406/Uqcrc2/mt-Cytb/Sdha/Tubb3/Dnal1/Ndufa6/Vdac1/Plcb4/Uqcrb/*  *Dnah10/Tubb4a/Cox8a/Plcb2/Dnai1/Hap1/Ndufs8/Uqcrc1/Cltc/Ndufs1/Ndufa12-ps/Gpr68/Uqcrfs1/Tuba4a/Tubb5/Cox7c/Ndufv1/Cox6c/Dnah5/Atp5d/Cyc1/Ndufa4/mt-Nd2/mt-Nd4/Ndufa10/Dnai2/Cox5a/Psmd12/Adrm1/Kcnj10/Ndufs2/Dnah6/*  *Tuba1c/Wipi2/Ndufb7/Ndufv2/Gm12338/Gm3756/Ndufs3/Tubb4b-ps1/Bax/Gm2962/*  *Psmd6/Atp5o/Ulk2/Ndufb8/Ndufb3/Klc2/Cox4i1/Creb3l4/Atp5e/Psmc1/Polr2d/Atp5pb/Uqcr11/Ndufa1/Cox5b/Cox7b/Dnah17/Gm4459/Psmd3/Hip1/Ndufab1-ps/Ndufa13/*  *E330011O21Rik/Psmb2/Gm20257/Sdhd/Ndufa11/Cltb/Vdac2/Atp5g2/Cox5b-ps/*  *Uqcrq/Psmc6/Atp5g3/Dctn1/Psmc2/Atp5b/Ndufc1/Uqcr10/Atg2a/Vdac3/Gm16418/Ppargc1a/Psmd4/Gpx1/Atg101/Ndufb4/Actr1a/Gm12337/Psmb6/Casp3/Ndufab1/Cox6a1/Klc1/Vdac3-ps1/Ndufs5/Taf4b/Ndufa2/Ndufb6/Grin2b/Gm6467/Atp5pb-ps/*  *Ndufc2/Psma4/Plcb3/Ndufs6b/Ndufs6/Psmb3/Psma3/Gpx8/Cox6b1/Polr2j/Psmb1/Slc1a2/Ap2m1/Gm16089/Sem1/Ppif/Dnah11/Gm8437/Dnah2/Gpx6/Cox7a1/Cox4i2/Gm10053/Tuba3a/Gm28437/Ndufb2/Gm14794/Gm10175/Dnah7a/mt-Co1/Psma7/*  *Dnali1/Grin1/Ndufs4/Gm13835/Cycs/Polr2a/Ap2b1/Htt/Dnah8/Cox7a2/Klc4/Gm14150/Gm4950/Polr2h/Creb3l2/Psmc3/Ndufa7/Gm7591/Psmd8/Psmd9/Kif5b/4930447K03Rik/Gm15694/Psma2/mt-Co3/Gm12251/Gm28661/Polr2k/Gria4/Gm13339/Polr2f/*  *A730017L22Rik/Psma6/Psmb4/Tuba5-ps/Psma5/ENSMUSG00000120425/Atg13/*  *Map3k10/Dnal4/Creb3l3/Gm5436/Gm11966/Gm49450/Gria1/Ulk1/Gm6969/Ndufa8/Psmd2/Ap2s1/Actr10/Gm44040/Slc25a5/Gm6444/Hdac1-ps/Gm29216/Cox6a2/*  *Ap2m1-ps/Psma5-ps* |
| mmu04714 | Thermogenesis | *Bmp8b/Adcy7/Acsl1/Cox6b2/Mapk13/Adcy8/Rptoros/Adcy2/Gm26588/Klb/Cnr1/mt-Nd3/Prdm16/Adcy3/Mgll/Adcy1/Ndufaf4/Gm10925/Ndufb5/Acsl5/Cpt1c/Ndufb10/Fgfr1/Ndufv3/Atp5a1/Ndufb9/Gm10250/Ndufaf1/4930481A15Rik/Creb5/Atp5h/Kdm1a/Atp5g1/Prkab2/Ndufa3/Acsl4/Adcy6/Ndufa5/Sdhc/Ndufa9/Atp5j/mt-Nd1/Gm10039/*  *Slc25a29/Ndufb11/Ndufs7/Atp5c1/Creb3l1/Sdhb/Cpt2/Uqcrc2/mt-Cytb/Sdha/Ndufa6/*  *Uqcrb/Cox8a/Smarcd2/Ndufs8/Uqcrc1/Ndufs1/Ndufa12-ps/Gpr68/Rps6ka3/Uqcrfs1/*  *Cox7c/Ndufv1/Cox6c/Atp5d/Cyc1/Ndufa4/mt-Nd2/mt-Nd4/Ndufa10/Cox5a/Ndufaf6/*  *Ndufs2/Lipe/Ndufaf8/Ndufb7/Ndufv2/Hras/Gm12338/Ndufaf2/Ndufs3/Prkag2/Prkaa1/Gm2962/Atp5k/Atp5o/Atp5l/Ndufb8/Nras/Ndufb3/Cox4i1/Ndufaf3/Creb3l4/Atp5e/Rps6kb2/Atp5pb/Uqcr11/Ndufa1/Cox5b/Cox7b/Gm4459/Ndufab1-ps/Coa5/Mlst8/*  *Ndufa13/Gm10221/Sdhd/Ndufa11/Atp5g2/Cox5b-ps/Uqcrq/Atp5g3/Prkaca/Atp5b/*  *Ndufc1/Uqcr10/Npr1/Gm16418/Cox10/Ppargc1a/Cox16/Ndufb4/Gm12337/Ndufab1/Cox6a1/ENSMUSG00000120971/Smarcc2/Map2k3/Ndufs5/Ndufa2/Prkg2/Ndufb6/Rps6ka1/Atp5pb-ps/Acsl3/Ndufc2/Smarcc1/Ndufs6b/Ndufs6/Cox6b1/Gm16089/*  *Gm8437/Prkg1/Slc25a20/Cox7a1/Cox4i2/Gm28437/Ndufb2/Gm14794/Gm10175/mt-Co1/Rps6kb1/Ndufs4/Sirt6/Prkaa2/Bmp8a/Cox7a2/Prkacb/Creb3l2/Ndufa7/Gm15694/mt-Co3/Gm12251/Mapk11/Gm28661/Coa7/Adcy5/Adcy9/Gm13339/Cox11/Actl6b/*  *Prkab1/Coa3/ENSMUSG00000120425/Creb3l3/Gm5436/Smarcd1/Gm11966/Fgf21/Cox18/Gm6969/Zfp516/Ndufa8/Kdm3b/Smarca4/Gm6444/Gm29216/Cox6a2* |
| mmu00071 | Fatty acid degradation | *Acsl1/Cyp4a31/Aldh9a1/Gcdh/Acadl/Acat3/Acsl5/Cpt1c/Acaa2/Eci1/Acat1/Gm39213/Aldh1b1/Acaa1b/Acadsb/Acsl4/Acat2/Cpt2/Adh4/Hadhb-ps/Hadha/Acox1/Adh7/*  *Acadvl/Echs1/Hadhb/Adh1/Hadh/Adh5/Acsbg1/Cyp4a10/Aldh2/Acadm/Acads/Acsl3/Eci3/Gm16559/Adh6-ps1/Cyp4a14/Aldh7a1/Acaa1a/Acox3* |
| mmu04666 | Fc gamma R-mediated phagocytosis | *Pak1/Sphk1/Marcks/Arf6/Pld1/Gab2/Limk1/Plcg2/Ncf1/Marcksl1/Asap3/Ptprc/Inpp5d/Mapk3/Gsn/Vasp/Was/Pik3cd/Pik3r3/Fcgr3/Arpc1b/Fcgr1/Pla2g4a/Vav1/Syk/Asap2/Akt2/Limk2/Hck/A830052D11Rik/Crk/Prkcb/Rac2/Plpp2/Cdc42/Rps6kb2/Prkcg/Myo10/Scin/Plpp1/Wasf3/Cfl2/Prkcd/Actr3b/Asap1/Pla2g6/Arpc2/Akt3/Fcgr2b/Actr3/Dnm2/Zfp996/Gm5637/Plpp3/Pld2/Inppl1/Vav3/Wasf2/Fcgr4/Pik3r2/Rps6kb1/Actr2/Mapk1/Plcg1/Actr3-ps/Pik3r1/Crkl/Dock1/Prkce/Arpc1a/Pla2g4d/Arpc5l* |
| mmu04640 | Hematopoietic cell lineage | *Cd24a/Csf2ra/Itga3/Cd34/Flt3/Itga2/H2-DMb1/Cd14/H2-Eb1/H2-Aa/H2-Ab1/Cd44/*  *Cd33/Cd59b/Itgam/Cd9/Cd1d1/Cd59a/Csf3r/H2-DMb2/Il6ra/Il3ra/H2-DMa/Cr2/Kitl/*  *Csf1r/Siglech/H2-Oa/Tnf/Fcgr1/Il7/Il11ra1/Il4ra/Mme/Thpo/H2-Ob/Itga6/Epor/Gypa/*  *H2-Eb2/Anpep/Itga4/Il1r1/Il1b/Il11/Il1r2/Il4/Tfrc/Cd8a/Cd3d/Cd5/Cd22/Csf1/*  *Gm16897/Il7r/Cd36/Cd8b1/Il2ra/Cr1l/Cd3e/Il9r/Cd55/Gp9* |
