# Supplementary material for: Inactivation of Atp7b Copper Transporter in Intestinal Epithelial Cells Is Associated with Altered Lipid Processing and Cell Growth Machinery Independent from Hepatic Copper Accumulation and Severity of Liver Histology
Source: Am J Pathol. 2025 Oct 16;196(2):407–27. doi: 10.1016/j.ajpath.2025.09.015 (PMC12881291; doi:10.1016/j.ajpath.2025.09.015)
Supplement: Supplemental Table S9 [file mmc17.docx]

**Supplemental Table S9. RNA-Seq top 20 KEGG pathways and associated differentially expressed genes in liver of 30-week *Atp7b*^ΔIEC^ mice (KEGG:** [**https://www.kegg.jp**](https://www.kegg.jp/)**).**

| **KEGG ID** | **Pathway Description** | **Gene Name** |
| --- | --- | --- |
| mmu00140 | Steroid hormone biosynthesis | *Ugt2b37/Cyp2d38-ps/Sult1e1/Ugt1a7c/Gm7652/Cyp2d37-ps/Cyp2b9/*  *Gm21049/Cyp2d35-ps/Cyp2d13* |
| mmu00980 | Metabolism of xenobiotics by cytochrome P450 | *Ugt2b37/Cyp2s1/Ugt1a7c/Gsto2/Gm7652/Gm21049/Gm3776/Mgst3* |
| mmu00982 | Drug metabolism - cytochrome P450 | *Ugt2b37/Ugt1a7c/Gsto2/Gm7652/Gm21049/Gm3776/Mgst3* |
| mmu05150 | Staphylococcus aureus infection | *Itgb2l/Krt18/Krt19/Cfh/Defb1/Camp* |
| mmu04621 | NOD-like receptor signaling pathway | *Cxcl1/Vdac2/Tbk1/Xiap/Ikbkg/Ripk1/Oas1g/Camp/Nek7/Pkn2/Itpr2* |
| mmu00860 | Porphyrin metabolism | *Ugt2b37/Cox10/Ugt1a7c/Gm7652/Gm21049* |
| mmu00053 | Ascorbate and aldarate metabolism | *Ugt2b37/Ugt1a7c/Gm7652/Gm21049* |
| mmu05204 | Chemical carcinogenesis - DNA adducts | *Ugt2b37/Ugt1a7c/Gsto2/Gm7652/Gm21049/Gm3776/Mgst3* |
| mmu04976 | Bile secretion | *Ugt2b37/Slc51b/Slc5a1/Ugt1a7c/Gm7652/Slc10a2/Gm21049* |
| mmu00830 | Retinol metabolism | *Ugt2b37/Cyp2s1/Retsat/Ugt1a7c/Gm7652/Cyp2b9/Gm21049* |
| mmu04657 | IL-17 signaling pathway | *Cxcl1/Fos/Tbk1/Lcn2/Ikbkg/S100a9* |
| mmu05207 | Chemical carcinogenesis - receptor activation | *Ugt2b37/Fos/Xiap/Ugt1a7c/Gsto2/Gm7652/Cyp2b9/Gm21049/Kpnb1/Gm3776/Mgst3* |
| mmu03440 | Homologous recombination | *Eme1/Pold2/Rad51b/Rad52* |
| mmu00983 | Drug metabolism - other enzymes | *Ugt2b37/Ugt1a7c/Gsto2/Gm7652/Gm21049/Gm3776/Mgst3* |
| mmu00040 | Pentose and glucuronate interconversions | *Ugt2b37/Ugt1a7c/Gm7652/Gm21049* |
| mmu04610 | Complement and coagulation cascades | *Gm16548/Itgb2l/Cfh/B430119L08Rik/Cpb2/Pros1* |
| mmu03450 | Non-homologous end-joining | *Dclre1c/Xrcc4* |
| mmu00730 | Thiamine metabolism | *Alpl/Gm14388* |
| mmu04936 | Alcoholic liver disease | *Cxcl1/Tbk1/Srebf1/Prkab1/Gm6439/Ikbkg/Ripk1* |
| mmu04622 | RIG-I-like receptor signaling pathway | *Tbk1/Ikbkg/Ripk1/Isg15* |
