# Supplementary material for: Inactivation of Atp7b Copper Transporter in Intestinal Epithelial Cells Is Associated with Altered Lipid Processing and Cell Growth Machinery Independent from Hepatic Copper Accumulation and Severity of Liver Histology
Source: Am J Pathol. 2025 Oct 16;196(2):407–27. doi: 10.1016/j.ajpath.2025.09.015 (PMC12881291; doi:10.1016/j.ajpath.2025.09.015)
Supplement: Supplemental Table S10 [file mmc18.docx]

**Supplemental Table S10. RNA-Seq top 20 Reactome pathways and associated differentially expressed genes in IECs of 16-week *Atp7b*^-/-^ mice (Reactome:** [**https://reactome.org/**](https://reactome.org/)**).**

| **Reactome ID** | **Pathway Description** | **Gene Name** |
| --- | --- | --- |
| R-MMU-8957322 | Metabolism of steroids | *Lgmn/Mvk/Akr1b7/Idi1/Akr1c19/Hsd3b3/Msmo1/Osbpl3/Acox2/Osbpl1a/Fabp6/Srd5a3/Srd5a2/Mbtps1/Slc10a2/Akr1c13/Cyp51/Hsd3b2/Akr1c12/Hmgcr/Hsd17b7/Slc10a1/Lss/Mvd/Dhcr24/Osbpl7/Nsdhl/Sqle* |
| R-MMU-191273 | Cholesterol biosynthesis | *Mvk/Idi1/Msmo1/Cyp51/Hmgcr/Hsd17b7/Lss/Mvd/Dhcr24/Nsdhl/Sqle* |
| R-MMU-70263 | Gluconeogenesis | *Aldoc/Aldoa/Pgk1/Slc37a2/Pcx/Got1/Pck2/Slc25a1/Pck1* |
| R-MMU-211859 | Biological oxidations | *Ces2a/Gsta1/Cyp3a13/Gm3776/Cyp3a11/Gm10639/Ces1d/Tbxas1/Cyp4f40/Cyp3a25/Cyp4b1/Por/Gclc/Ugt1a9/Gstm2/Ugp2/Ugt2b34/Adh1/Cyp2c55/Cyp2c66/Ces2c/Cyp51/Fmo1/Cyp2j6/Paox/Aldh1b1/Aoc1/Gsta2/Hsp90ab1/Sult1c2/Cyp4a10/Cyb5r3/Slc35d1/As3mt/Abhd10* |
| R-MMU-194068 | Bile acid and bile salt metabolism | *Akr1c19/Osbpl3/Acox2/Osbpl1a/Fabp6/Slc10a2/Akr1c13/Akr1c12/Slc10a1/Osbpl7* |
| R-MMU-2168880 | Scavenging of heme from plasma | *Apol10a/Apoa1/Apol7c/Apol11b/Apol8/Ambp/Apol10b* |
| R-MMU-211945 | Phase I - Functionalization of compounds | *Ces2a/Cyp3a13/Cyp3a11/Ces1d/Tbxas1/Cyp4f40/Cyp3a25/Cyp4b1/Por/Adh1/Cyp2c55/Cyp2c66/Ces2c/Cyp51/Fmo1/Cyp2j6/Paox/Aldh1b1/Aoc1/Hsp90ab1/Cyp4a10/Cyb5r3* |
| R-MMU-2132295 | MHC class II antigen presentation | *Ctse/Lgmn/Canx/Kif20a/Sh3gl2/Ctso/Osbpl1a/Racgap1/Kif2c/Kif23/Ctsl/Tuba3a/Ctss/Sar1b/Cenpe/Kif22/Dync1h1/Dnm3/Dnm2* |
| R-MMU-141424 | Amplification of signal from the kinetochores | *Cenpm/Nup85/Bub1b/Kntc1/Ndc80/Cdc20/Kif2c/Incenp/Cdca8/Cenpe/Spc24/Cenph/Plk1/Nuf2/Cenpa/Dync1h1/Nup133* |
| R-MMU-141444 | Amplification of signal from unattached kinetochores via a MAD2 inhibitory signal | *Cenpm/Nup85/Bub1b/Kntc1/Ndc80/Cdc20/Kif2c/Incenp/Cdca8/Cenpe/Spc24/Cenph/Plk1/Nuf2/Cenpa/Dync1h1/Nup133* |
| R-MMU-2024096 | HS-GAG degradation | *Hgsnat/Glb1/Glb1l/Sdc4/Sdc2/Gpc4* |
| R-MMU-196854 | Metabolism of vitamins and cofactors | *Cyb5a/Akr1b7/Nampt/Ptgs2/Akr1c19/Idh1/Pank3/Pcx/Apoa1/Slc2a1/Bco1/Slc5a6/Nmrk1/Ppcdc/Akr1c13/Akr1c12/Cyb5r3/Slc19a1/Sdc4/Sdc2/Slc23a1/Mthfd2/Gpc4/Nudt12/Gphn/Slc25a19* |
| R-MMU-2173782 | Binding and Uptake of Ligands by Scavenger Receptors | *Apol10a/Apoa1/Apol7c/Apol11b/Apol8/Cd36/Masp1/Ambp/Apol10b* |
| R-MMU-68877 | Mitotic Prometaphase | *Cenpm/Nup85/Bub1b/Kntc1/Ndc80/Ncaph/Cdc20/Haus8/Ncapd2/Kif2c/Tuba3a/Incenp/Cdca8/Cenpe/Spc24/Tubgcp3/Cenph/Cep250/Nek2/Plk1/Nuf2/Cenpa/Smc2/Cep131/Cdk5rap2/Dync1h1/Sfi1/Nup133* |
| R-MMU-2162123 | Synthesis of Prostaglandins (PG) and Thromboxanes (TX) | *Tbxas1/Ptgs2/Akr1c19/Akr1c13/Akr1c12* |
| R-MMU-193048 | Androgen biosynthesis | *Hsd3b3/Srd5a3/Srd5a2/Hsd3b2* |
| R-MMU-844456 | The NLRP3 inflammasome | *Nlrp3/Hsp90ab1/Pycard/Txnip* |
| R-MMU-70326 | Glucose metabolism | *Pfkl/Aldoc/Pfkfb3/Aldoa/Pgk1/Slc37a2/Pcx/Got1/Pck2/Nup85/Slc25a1/Ndc1/Pck1/Nup133* |
| R-MMU-975634 | Retinoid metabolism and transport | *Akr1b7/Akr1c19/Apoa1/Bco1/Akr1c13/Akr1c12/Sdc4/Sdc2/Gpc4* |
| R-MMU-8847993 | ERBB2 Activates PTK6 Signaling | *Erbb2/Hbegf/Btc/Erbb3* |
