# Supplementary material for: Inactivation of Atp7b Copper Transporter in Intestinal Epithelial Cells Is Associated with Altered Lipid Processing and Cell Growth Machinery Independent from Hepatic Copper Accumulation and Severity of Liver Histology
Source: Am J Pathol. 2025 Oct 16;196(2):407–27. doi: 10.1016/j.ajpath.2025.09.015 (PMC12881291; doi:10.1016/j.ajpath.2025.09.015)
Supplement: Supplemental Table S11 [file mmc19.docx]

**Supplemental Table S11. RNA-Seq top 20 Reactome pathways and associated differentially expressed genes in IECs of 16-week *Atp7b*^ΔIEC^ mice (Reactome:** [**https://reactome.org/**](https://reactome.org/)**).**

| **Reactome ID** | **Pathway Description** | **Gene Name** |
| --- | --- | --- |
| R-MMU-211945 | Phase I - Functionalization of compounds | *Cyp3a16/Cyp2c55/Ces1c/Cyp39a1/Cyp2a5/Cyp1b1/Ces1b* |
| R-MMU-211859 | Biological oxidations | *Cyp3a16/Cyp2c55/Ces1c/Gstm1/Slc26a1/Cyp39a1/Cyp2a5/Cyp1b1/Ces1b* |
| R-MMU-211897 | Cytochrome P450 - arranged by substrate type | *Cyp3a16/Cyp2c55/Cyp39a1/Cyp2a5/Cyp1b1* |
| R-MMU-203615 | eNOS activation | *Hsp90aa1/Ddah1* |
| R-MMU-211981 | Xenobiotics | *Cyp3a16/Cyp2c55/Cyp2a5* |
| R-MMU-392517 | Rap1 signalling | *Rasgrp2/Rap1gap* |
| R-MMU-5250913 | Positive epigenetic regulation of rRNA expression | *Hist1h2ao/Polr1b/Cd3eap/Mybbp1a* |
| R-MMU-5250924 | B-WICH complex positively regulates rRNA expression | *Hist1h2ao/Polr1b/Cd3eap/Mybbp1a* |
| R-MMU-202131 | Metabolism of nitric oxide | *Hsp90aa1/Ddah1* |
| R-MMU-203765 | eNOS activation and regulation | *Hsp90aa1/Ddah1* |
| R-MMU-193775 | Synthesis of bile acids and bile salts via 24-hydroxycholesterol | *Slc27a2/Cyp39a1* |
| R-MMU-8953897 | Cellular responses to external stimuli | *Mt1/Pot1b/Hmga1b/Hsph1/Hist1h2ao/Hsp90aa1/Stip1/Gsr/Hspa4l/Mapkapk5* |
| R-MMU-2262752 | Cellular responses to stress | *Pot1b/Hmga1b/Hsph1/Hist1h2ao/Hsp90aa1/Stip1/Gsr/Hspa4l/Mapkapk5* |
| R-MMU-212165 | Epigenetic regulation of gene expression | *Hist1h2ao/Polr1b/Cd3eap/Mybbp1a* |
| R-MMU-211976 | Endogenous sterols | *Cyp39a1/Cyp1b1* |
| R-MMU-5423646 | Aflatoxin activation and detoxification | *Cyp3a16/Cyp2a5* |
| R-MMU-5669034 | TNFs bind their physiological receptors | *Edaradd/Tnfrsf18* |
| R-MMU-73777 | RNA Polymerase I Chain Elongation | *Polr1b/Cd3eap* |
| R-MMU-2559586 | DNA Damage/Telomere Stress Induced Senescence | *Pot1b/Hmga1b/Hist1h2ao* |
| R-MMU-73772 | RNA Polymerase I Promoter Escape | *Polr1b/Cd3eap* |
