# Supplementary material for: Inactivation of Atp7b Copper Transporter in Intestinal Epithelial Cells Is Associated with Altered Lipid Processing and Cell Growth Machinery Independent from Hepatic Copper Accumulation and Severity of Liver Histology
Source: Am J Pathol. 2025 Oct 16;196(2):407–27. doi: 10.1016/j.ajpath.2025.09.015 (PMC12881291; doi:10.1016/j.ajpath.2025.09.015)
Supplement: Supplemental Table S12 [file mmc20.docx]

**Supplemental Table S12. RNA-Seq top 20 Reactome pathways and associated differentially expressed genes in IECs of 30-week *Atp7b*^-/-^ mice (Reactome:** [**https://reactome.org/**](https://reactome.org/)**).**

| Reactome ID | Pathway Description | Gene Name |
| --- | --- | --- |
| R-MMU-191273 | Cholesterol biosynthesis | *Mvk/Hmgcs1/Fdft1/Tm7sf2/Mvd/Nsdhl/Sqle/Hsd17b7/Cyp51/Pmvk/Fdps/Hmgcr/Msmo1* |
| R-MMU-3371497 | HSP90 chaperone cycle for steroid hormone receptors (SHR) | *Dync1h1/Stip1/Hsp90aa1/Hspa1b/Hspa8/Hspa1a/Dctn6/Tuba1a/Dynll1/Fkbp4/Tubb2b/Hsp90ab1/Tubb2a/Dctn2* |
| R-MMU-6811436 | COPI-independent Golgi-to-ER retrograde traffic | *Dync1h1/Dctn6/Tuba1a/Dynll1/Pafah1b3/Tubb2b/Tubb2a/Rab6a/Dctn2/Pafah1b2/Pla2g4a/Rab18/Pafah1b1* |
| R-MMU-2132295 | MHC class II antigen presentation | *Dync1h1/Lgmn/Dnm1/Kif3a/Dctn6/Ctsa/Tuba1a/Dynll1/Ctss/Sec24b/H2-Ob/Tubb2b/H2-Oa/Tubb2a/Kif4/Dctn2/Ap1m1/Klc2/Kif11* |
| R-MMU-8957322 | Metabolism of steroids | *Mvk/Nr1h4/Lgmn/Hmgcs1/Fdft1/Tm7sf2/Mvd/Nsdhl/Slc10a2/Sqle/Hsd17b7/Cyp51/Srebf2/Cyp27b1/Pmvk/Fdps/Stard4/Hmgcr/Msmo1* |
| R-MMU-1679131 | Trafficking and processing of endosomal TLR | *Lgmn/Tlr7/Hsp90b1/Ctss/Tlr9* |
| R-MMU-8964038 | LDL clearance | *Ldlr/Pcsk9/Ldlrap1/Apob/Soat1/Npc1* |
| R-MMU-6798695 | Neutrophil degranulation | *H2-Q10/Aldoc/Tom1/Hbb-bs/Dync1h1/Hbb-bt/Hsp90aa1/Hspa1b/Fuca1/Hspa8/Dynlt1c/Gsdmd/Slc11a1/S100a11/Cybb/Atp8b4/Neu1/Hspa1a/Dsp/Ctsa/Ncstn/Ctsz/Sirpa/Dynll1/Fcgr4/Ctss/Magt1/Erp44/Mospd2/Serpina1e/Abca13/Grn/Gsn/Hsp90ab1/Pnp/Hebp2/Ceacam10/Rab6a/Cd44/Serpinb1a/Acaa1a/Snap25/Ap1m1/Cfp/Mvp/Diaph1/Stom/Pgm1/Pafah1b2/Rab18/Gaa/Mlec/Trappc1/Psmd11/Man2b1/Mettl7a1/Cd300a* |
| R-MMU-1237044 | Erythrocytes take up carbon dioxide and release oxygen | *Hbb-bs/Hbb-bt/Hba-a1/Hba-a2/Slc4a1* |
| R-MMU-1480926 | O2/CO2 exchange in erythrocytes | *Hbb-bs/Hbb-bt/Hba-a1/Hba-a2/Slc4a1* |
| R-MMU-168898 | Toll-Like Receptors Cascades | *Saa1/Nfkb2/Ager/Lgmn/Dnm1/Tlr7/Ly86/Birc3/Hsp90b1/Ctss/Chuk/Apob/S100a1/Uba52/Ly96/Tlr9/Rps6ka2/Ppp2ca/Peli1/Mapk11/Tnip2* |
| R-MMU-2173782 | Binding and Uptake of Ligands by Scavenger Receptors | *Saa1/Hbb-bs/Hbb-bt/Apoe/Hba-a1/Hba-a2/Apol9b/Hsp90b1/Apob* |
| R-MMU-2028269 | Signaling by Hippo | *Wwtr1/Amotl2/Stk3/Amot/Lats1/Mob1b* |
| R-MMU-2470946 | Cohesin Loading onto Chromatin | *Pds5b/Nipbl/Pds5a/Wapl* |
| R-MMU-422475 | Axon guidance | *Egfr/Hsp90aa1/Pik3r3/Col9a3/Mapk13/Nrcam/Arhgef12/Dnm1/Epha4/Vegfa/Tuba1a/Ncstn/Itsn1/Rras/Evl/Plxna1/Pip5k1c/Sptb/Col4a1/Tubb2b/Hsp90ab1/Tubb2a/Kif4/Uba52/Shc1/Frs2/Slit3/Arhgef28/Yes1/Nck1/Mapk11/Sptbn4/Fgfr1/Sema6d* |
| R-MMU-8856688 | Golgi-to-ER retrograde transport | *Dync1h1/Kif3a/Dctn6/Tuba1a/Dynll1/Kif27/Pafah1b3/Tubb2b/Tubb2a/Kif4/Use1/Rab6a/Dctn2/Pafah1b2/Pla2g4a/Rab18/Klc2/Pafah1b1/Kif11* |
| R-MMU-3371571 | HSF1-dependent transactivation | *Hsp90aa1/Hspa1b/Hspa8/Camk2g/Hspa1a/Hsp90ab1* |
| R-MMU-168643 | Nucleotide-binding domain, leucine rich repeat containing receptor (NLR) signaling pathways | *Mapk13/Sugt1/Birc3/Casp4/Chuk/Hsp90ab1/Uba52/Nlrp3/Mapk11* |
| R-MMU-2500257 | Resolution of Sister Chromatid Cohesion | *Dync1h1/Nup98/Clip1/Pds5b/Tuba1a/Dynll1/Pds5a/Ska2/Tubb2b/Gm5601/Tubb2a/Rps27rt/Taok1/Nup160/Ppp2ca/Clasp2/Wapl/Pafah1b1* |
| R-MMU-163210 | Formation of ATP by chemiosmotic coupling | *ATP8/Atp5k/Atp5j2/ATP6/Atp5f1* |
