# Supplementary material for: Inactivation of Atp7b Copper Transporter in Intestinal Epithelial Cells Is Associated with Altered Lipid Processing and Cell Growth Machinery Independent from Hepatic Copper Accumulation and Severity of Liver Histology
Source: Am J Pathol. 2025 Oct 16;196(2):407–27. doi: 10.1016/j.ajpath.2025.09.015 (PMC12881291; doi:10.1016/j.ajpath.2025.09.015)
Supplement: Supplemental Table S13 [file mmc21.docx]

**Supplemental Table S13. RNA-Seq top 20 Reactome pathways and associated differentially expressed genes in IECs of 30-week *Atp7b*^ΔIEC^ mice (Reactome:** [**https://reactome.org/**](https://reactome.org/)**).**

| **Reactome ID** | **Pathway Description** | **Gene Name** |
| --- | --- | --- |
| R-MMU-1428517 | The citric acid (TCA) cycle and respiratory electron transport | *Ndufv1/Slc16a8/Sdhb/Atp5o/Ndufs8/Atp5b/Ndufa8/Atp5j2/Ndufb8/Atp5k/Ndufs7/Ndufc1/Atp5e/Ndufb6/Atp5g3/Ndufb10/Atp5a1/Ndufs5/Ppard/Aco2/ND3/Ndufv3/Sdhc/Ndufa3/Atp5h/Sdhd/Ndufb9* |
| R-MMU-163200 | Respiratory electron transport, ATP synthesis by chemiosmotic coupling, and heat production by uncoupling proteins. | *Ndufv1/Atp5o/Ndufs8/Atp5b/Ndufa8/Atp5j2/Ndufb8/Atp5k/Ndufs7/Ndufc1/Atp5e/Ndufb6/Atp5g3/Ndufb10/Atp5a1/Ndufs5/ND3/Ndufv3/Ndufa3/Atp5h/Ndufb9* |
| R-MMU-1236978 | Cross-presentation of soluble exogenous antigens (endosomes) | *Psmb10/Psma2/Psmb4/Psmc3/Psma5/Psmb9/Psme2b/Psmd7/Psme1/Psmc5/Psmb8/Psmb5/Mrc2/Psmc2* |
| R-MMU-450531 | Regulation of mRNA stability by proteins that bind AU-rich elements | *Xpo1/Psmb10/Khsrp/Psma2/Psmb4/Psmc3/Uba52/Psma5/Psmb9/Psme2b/Psmd7/Psme1/Exosc3/Psmc5/Psmb8/Psmb5/Psmc2/Akt1* |
| R-MMU-69229 | Ubiquitin-dependent degradation of Cyclin D1 | *Psmb10/Psma2/Psmb4/Psmc3/Uba52/Psma5/Psmb9/Psme2b/Psmd7/Psme1/Psmc5/Psmb8/Psmb5/Psmc2* |
| R-MMU-75815 | Ubiquitin-dependent degradation of Cyclin D | *Psmb10/Psma2/Psmb4/Psmc3/Uba52/Psma5/Psmb9/Psme2b/Psmd7/Psme1/Psmc5/Psmb8/Psmb5/Psmc2* |
| R-MMU-68827 | CDT1 association with the CDC6:ORC:origin complex | *Psmb10/Psma2/Psmb4/Psmc3/Uba52/Psma5/Psmb9/Psme2b/Psmd7/Psme1/Psmc5/Mcm8/Psmb8/Psmb5/Psmc2* |
| R-MMU-349425 | Autodegradation of the E3 ubiquitin ligase COP1 | *Psmb10/Psma2/Psmb4/Psmc3/Uba52/Psma5/Psmb9/Psme2b/Psmd7/Psme1/Psmc5/Psmb8/Psmb5/Psmc2* |
| R-MMU-69601 | Ubiquitin Mediated Degradation of Phosphorylated Cdc25A | *Psmb10/Psma2/Psmb4/Psmc3/Uba52/Psma5/Psmb9/Psme2b/Psmd7/Psme1/Psmc5/Psmb8/Psmb5/Psmc2* |
| R-MMU-69610 | p53-Independent DNA Damage Response | *Psmb10/Psma2/Psmb4/Psmc3/Uba52/Psma5/Psmb9/Psme2b/Psmd7/Psme1/Psmc5/Psmb8/Psmb5/Psmc2* |
| R-MMU-69613 | p53-Independent G1/S DNA damage checkpoint | *Psmb10/Psma2/Psmb4/Psmc3/Uba52/Psma5/Psmb9/Psme2b/Psmd7/Psme1/Psmc5/Psmb8/Psmb5/Psmc2* |
| R-MMU-8939902 | Regulation of RUNX2 expression and activity | *Psmb10/Psma2/Psmb4/Psmc3/Uba52/Psma5/Psmb9/Psme2b/Psmd7/Psme1/Psmc5/Psmb8/Psmb5/Psmc2* |
| R-MMU-187577 | SCF(Skp2)-mediated degradation of p27/p21 | *Psmb10/Psma2/Psmb4/Psmc3/Uba52/Psma5/Psmb9/Psme2b/Psmd7/Psme1/Psmc5/Psmb8/Psmb5/Ptk6/Psmc2* |
| R-MMU-8941858 | Regulation of RUNX3 expression and activity | *Psmb10/Psma2/Psmb4/Psmc3/Uba52/Psma5/Psmb9/Psme2b/Psmd7/Psme1/Psmc5/Psmb8/Psmb5/Psmc2* |
| R-MMU-69202 | Cyclin E associated events during G1/S transition | *Psmb10/Psma2/Psmb4/Psmc3/Uba52/Psma5/Psmb9/Psme2b/Psmd7/Psme1/Psmc5/Psmb8/Psmb5/Ptk6/Psmc2/Akt1* |
| R-MMU-5358346 | Hedgehog ligand biogenesis | *Psmb10/Disp2/Psma2/Psmb4/Psmc3/Uba52/Psma5/Psmb9/Psme2b/Psmd7/Psme1/Psmc5/Psmb8/Psmb5/Psmc2* |
| R-MMU-174113 | SCF-beta-TrCP mediated degradation of Emi1 | *Psmb10/Psma2/Psmb4/Psmc3/Uba52/Psma5/Psmb9/Psme2b/Psmd7/Psme1/Psmc5/Psmb8/Psmb5/Psmc2* |
| R-MMU-450408 | AUF1 (hnRNP D0) binds and destabilizes mRNA | *Psmb10/Psma2/Psmb4/Psmc3/Uba52/Psma5/Psmb9/Psme2b/Psmd7/Psme1/Psmc5/Psmb8/Psmb5/Psmc2* |
| R-MMU-4641257 | Degradation of AXIN | *Psmb10/Psma2/Psmb4/Psmc3/Uba52/Psma5/Psmb9/Psme2b/Psmd7/Psme1/Psmc5/Psmb8/Psmb5/Psmc2* |
| R-MMU-8854050 | FBXL7 down-regulates AURKA during mitotic entry and in early mitosis | *Psmb10/Psma2/Psmb4/Psmc3/Uba52/Psma5/Psmb9/Psme2b/Psmd7/Psme1/Psmc5/Psmb8/Psmb5/Psmc2* |
